# Phylotranscriptomics Resolves the Phylogeny of Pooideae and Uncovers Factors for Their Adaptive Evolution

**DOI:** 10.1093/molbev/msac026

**Published:** 2022-02-03

**Authors:** Lin Zhang, Xinxin Zhu, Yiyong Zhao, Jing Guo, Taikui Zhang, Weichen Huang, Jie Huang, Yi Hu, Chien-Hsun Huang, Hong Ma

**Affiliations:** 1 State Key Laboratory of Genetic Engineering and Ministry of Education Key Laboratory of Biodiversity Sciences and Ecological Engineering, Institute of Plant Biology, Institute of Biodiversity Sciences, School of Life Sciences, Fudan University, Shanghai, China; 2 College of Life Sciences, Xinyang Normal University, Xinyang, China; 3 Department of Biology, Huck Institutes of Life Sciences, The Pennsylvania State University, University Park, PA, USA

**Keywords:** Pooideae, nuclear phylogeny, character evolution, gene duplication, diversification, cold adaptive evolution

## Abstract

Adaptation to cool climates has occurred several times in different angiosperm groups. Among them, Pooideae, the largest grass subfamily with ∼3,900 species including wheat and barley, have successfully occupied many temperate regions and play a prominent role in temperate ecosystems. To investigate possible factors contributing to Pooideae adaptive evolution to cooling climates, we performed phylogenetic reconstruction using five gene sets (with 1,234 nuclear genes and their subsets) from 157 transcriptomes/genomes representing all 15 tribes and 24 of 26 subtribes. Our phylogeny supports the monophyly of all tribes (except Diarrheneae) and all subtribes with at least two species, with strongly supported resolution of their relationships. Molecular dating suggests that Pooideae originated in the late Cretaceous, with subsequent divergences under cooling conditions first among many tribes from the early middle to late Eocene and again among genera in the middle Miocene and later periods. We identified a cluster of gene duplications (CGD5) shared by the core Pooideae (with 80% Pooideae species) near the Eocene–Oligocene transition, coinciding with the transition from closed to open habitat and an upshift of diversification rate. Molecular evolutionary analyses homologs of *CBF* for cold resistance uncovered tandem duplications during the core Pooideae history, dramatically increasing their copy number and possibly promoting adaptation to cold habitats. Moreover, duplication of *AP1*/*FUL*-like genes before the Pooideae origin might have facilitated the regulation of the vernalization pathway under cold environments. These and other results provide new insights into factors that likely have contributed to the successful adaptation of Pooideae members to temperate regions.

## Introduction

The success of plants depends on their adaptation to changing environments, such as the global cooling climate from the mid-Eocene (∼46 Million years ago, [Ma]) to late Oligocene (∼27 Ma), which included the contraction of tropics and expansion of temperate regions (Zachos et al. 2001; Eldrett et al. 2009). In response to such cooling climates, some angiosperm families (such as Saxifragaceae and Brassicaceae) have evolved the ability to adapt to seasonal climates with cold periods and are now regarded as temperate lineages (Deng et al. 2015; Huang, Sun, et al. 2016). Although such adaptation to cooling climates can be found in a number of lineages, further diversification of the corresponding taxon groups is a relatively rare accomplishment (Donoghue 2008; Watcharamongkol et al. 2018), as supported by the dramatically lower biodiversity in the temperate zone than those in the tropics (Mittelbach et al. 2007) and large-scale extinctions associated with cooling periods (Ivany et al. 2000). Furthermore, seasonal low temperature remains a major challenge nowadays even for temperate biomes. Hence, how plants have evolved the ability to thrive in temperate regions is of great interest and significance.

The grasses constitute the fifth largest angiosperm family, Poaceae, with 12 subfamilies, 768 genera and ∼11,500 species (Soreng et al. 2017). They are ecologically dominant in grasslands and bamboo forests, accounting for ∼40% of Earth’s terrestrial areas (Gibson 2008; Soreng et al. 2017; Hodkinson 2018). Among grass subfamilies, Pooideae, also called temperate grasses or cool-season grasses, are the largest subfamily with ∼3,900 species (34% of Poaceae species) (Soreng et al. 2017), similar in size to moderately large families such as Brassicaceae (∼3,700 species, with *Arabidopsis* and cabbage), Rosaceae (∼3,000 species, rose, apple, and peach) and Apiaceae (∼3,700 species, carrot and celery), and larger than 96% of angiosperm families (Christenhusz and Byng 2016). Pooideae contain important crops like wheat (*Triticum aestivum*), barley (*Hordeum vulgare*), and oat (*Avena sativa*), as well as pasture resources like ryegrass (*Lolium perenne*) and orchardgrass (*Dactylis glomerata*) (Hodkinson 2018). Unlike other large grass subfamilies, Pooideae are ecologically dominant in temperate regions (predominately in the northern hemisphere), with some in subtropics, tropical highlands, even polar regions (Alberdi et al. 2002; Brysting et al. 2004; Namaganda and Lye 2008). Their habitats range from relatively closed forest margin and understory (Renvoize 1985; Pennington et al. 2004) to open grasslands including steppe, tundra, and alpine meadow (Kellogg 2015), demonstrating successful adaptation to cool and arid environments (Blattner 2006), temperate wetland (Neill 1993; Lucassen et al. 2000), deserts (Caldwell et al. 1981; Yu et al. 2004), and saline–alkaline areas (Zhang, Liu, et al. 2020).

The estimation of the dates of origin and diversification of major clades (sublineages) of Pooideae can provide clues about evolutionary adaptation to temperate regions (Pimentel et al. 2017); however, previous molecular inferences yielded a wide range of estimated ages for the origin of Pooideae from the Cretaceous–Palaeocene boundary to the mid-Eocene (∼66–46 Ma) (Prasad et al. 2011; Christin et al. 2014; Schubert et al. 2019). Nevertheless, regardless of the estimated time of the Pooideae origin, Pooideae lineages have likely experienced global cooling periods since the Eocene and diversified to a great extent in particularly large groups such as Poeae, and further become dominant in temperate region nowadays (Spriggs et al. 2014; Pimentel et al. 2017; Schubert et al. 2019). Furthermore, the distribution of species richness in Pooideae varies dramatically at the tribe level (Soreng et al. 2017), with a single tribe Poeae having three-fifths of genera (121 of 202) and species (∼2,562 of 3,900 spp.). Poeae and three other tribes Bromeae (∼165 spp.), Littledaleeae (∼4 spp.), and Triticeae (∼501 spp.) together are referred to as the “core Pooideae” (Soreng and Davis 1998), including >80% of Pooideae species diversity. Among the remaining 11 tribes, three have >100 species: Stipeae (∼527 spp.), Bromeae (165 spp.), and Meliceae (158 spp.), whereas others have <30 species (Duthieeae, Diarrheneae, and Brachyelytreae), or only one species (Ampelodesmeae, Brylkinieae, Phaenospermateae, Nardeae, and Lygeeae). Hence, greater sampling is needed to gain further understanding of divergence times and diversification in Pooideae.

Temperate grasses have also evolved diverse morphologies under a variety of environments, including different inflorescences (panicle, raceme, or spike) and variation in flowers (florets) number within a relatively small and compact “branch,” called spikelet, of the inflorescence (Kellogg 2015). Each floret includes bracts (usually two: lemma and palea), lodicules (2–3), stamens, and the ovary. It is possible that some of the diverse morphologies might be associated with adaptive evolution; for example, the Triticeae species (such as wheat and barley) with spike inflorescences are often distributed in regions with arid and cold environments, whereas Meliceae species (such as *Glyceria*) with inflorescence of the panicle type are found in wetlands with relative warm climates. Hence, analysis of evolutionary histories of these traits can help understand adaptive evolution of Pooideae; however, these have not been extensively investigated across Pooideae. Moreover, molecular studies of adaptation to cold temperatures in Pooideae cereals (e.g., wheat and barley) and relatives (e.g., *Brachypodium*) showed that *CBF/DREB1* and *COR* related genes can mediate reduction of cellular chilling/freezing damage. In addition, cold-dependent flowering (vernalization) is important for adaptation to cold climates and involves functions of *VRN1*, *VRN2*, and *VRN3* and other genes (Yan et al. 2003, 2004, 2006; Sandve and Fjellheim 2010; Zhao et al. 2015; Xu and Chong 2018). These findings suggest that genetic innovation for responses of cold might have contributed to the adaptation of Pooideae lineages to temperate regions (Zhong et al. 2018), but the evolutionary history of traits and genes related to cold adaptation in Pooideae at the tribe/subtribe level has not been examined.

Gene duplication (GD) is a major process supporting functional innovations and evolutionary adaptation to environmental opportunities (Crow et al. 2006; Innan and Kondrashov 2010; Panchy et al. 2016) and can be due to whole-genome duplication (WGD), tandem duplication, and transposon-mediated duplication (Panchy et al. 2016). WGD produces massive raw materials available for subsequent genetic variation (Van de Peer et al. 2017), is very common during angiosperm evolution (Jiao et al. 2011; Huang, Zhang, et al. 2016; Ren et al. 2018; Leebens-Mack et al. 2019; Guo et al. 2020; Zhang, Zhang, et al. 2020; Zhao et al. 2021), and has been associated with increased adaptive potential and organismal diversification (Arnold 1992; Fawcett et al. 2009; Tank et al. 2015; Landis et al. 2018; Wu et al. 2020). Tandem duplication can result from unequal crossing-over between similar gene copies that are positioned closely on a chromosome (Zhang 2003). Such tandemly duplicate copies are widespread in plants, contribute to gene family expansion (Cannon et al. 2004), and can exhibit variation in expression and function among copies (Xu et al. 2020). In addition, transposon-mediated duplication and retroduplication have been found in grasses including rice (Jiang et al. 2004; Wang et al. 2006) and maize (Du et al. 2009). In a previous study, Sandve and Fjellheim (2010) proposed that duplications of cold related genes have played a role in Pooideae adaptation to cool climates during the global cooling period (∼33 Ma). However, the number and positions of such GDs in the Pooideae phylogeny remain largely unclear.

Macroevolutionary analyses of adaptation, divergence time, morphological transitions, and gene families in Pooideae depend on a robust Pooideae phylogeny with well-resolved relationships as mentioned previously (Tank et al. 2015; Smith, Brown, Yang, et al. 2018; Wang et al. 2019). The molecular phylogeny of grasses has greatly improved in recent decades (GPWG 2001; Schneider et al. 2011; GPWGII 2012; Kellogg 2015; Soreng et al. 2017; Saarela et al. 2018). For example, an integrated analysis of molecular and morphological data of 62 taxa supported classifying Pooideae into 13 tribes (GPWG 2001) and a subsequent study with greater sampling supported the same 13 tribes and divided the largest tribe, Poeae, into Poeae 1 and Poeae 2, each with multiple subtribes (GPWGII 2012) (supplementary fig. S2, Supplementary Material online). More recent reports recognized ten Pooideae tribes and 15 Poeae subtribes (Kellogg 2015), 14 tribes and 21 subtribes (Soreng et al. 2015), or 15 tribes and 26 subtribes in Poeae (Soreng et al. 2017) (supplementary figs. S1 and S2, Supplementary Material online); the increases in numbers of tribes and subtribes in the latter two studies reflect differences in relationships for some genera and have relied largely on phylogenetic analyses using two plastid genes (*matK* and *ndhF*) from 448 grasses and other phylogenetic and morphological information (Soreng et al. 2015, 2017). The studies have divided Poeae into the two clades as reported previously (Soreng et al. 1990, 2017), namely Poeae chloroplast group 1 (PCG1) and Poeae chloroplast group 2 (PCG2) (supplementary fig. S2, Supplementary Material online).

However, many of the relationships among Pooideae tribes and subtribes remain unclear or inconsistent (supplementary figs. S1 and S2, Supplementary Material online), including the relationships among the five early divergent tribes (Duthieeae, Brylkinieae, Meliceae, Phaenospermateae, and Stipeae) (supplementary fig. S1, Supplementary Material online) (Schneider et al. 2009, 2011; GPWGII 2012; Soreng et al. 2015, 2017). In addition to differences in the number of Poeae subtribes (supplementary fig. S2, Supplementary Material online) (Soreng et al. 2007, 2015, 2017; Kellogg 2015), the PCG1 and PCG2 groups of Poeae subtribes from plastid sequence analyses are not supported by nuclear phylogenetic analyses, with some members of PCG2 nested within PCG1 as well as the paraphyly of other PCG2 subtribes (Quintanar et al. 2007; Schneider et al. 2009; Saarela et al. 2017). To clarify the macroevolutionary history of Pooideae at the tribe and subtribe levels, both taxon sampling representing tribes and subtribes and sufficient phylogenetically informative markers are needed; the latter can be achieved by using a large number of nuclear genes from next-generation sequencing (Li et al. 2003; Ebersberger et al. 2009; Zimmer and Wen 2015). Several nuclear phylogenomic studies have shown their unprecedented potential to uncover well-resolved species relationships (Duarte et al. 2010; Zeng et al. 2014; Huang, Sun, et al. 2016; Xiang et al. 2017; Qi et al. 2018; Cai et al. 2019; Leebens-Mack et al. 2019).

Here, we used 157 Pooideae data sets representing all tribes and 92% of Poeae subtribes to identify putative orthologous genes for the reconstruction of Pooideae phylogeny. We further conducted molecular clock analysis using the nuclear Pooideae phylogeny and historical temperature changes as references. Moreover, ancestral character analyses and GD detection provide clues to the possible factors of adaptive evolution of temperate grasses.

## Results

### Taxon Sampling and Transcriptome Sequencing

We sampled a total of 157 species of Pooideae to represent all 15 tribes, and 24 of 26 subtribes of Poeae (supplementary table S1, Supplementary Material online), following the Pooideae classification (such as supertribes, tribes, and subtribes) of Soreng et al. (2017) unless otherwise specified. For the three largest tribes, the sampled taxa cover ∼92% of the Poeae subtribes (the largest tribe with 121 genera; two unsampled subtribes together have only six species) and nearly half of genera of each of the tribes Stipeae (13 of 28 genera) and Triticeae (13 of 27 genera). For 12 other tribes that have one to eight genera (such as Brachyelytreae [1], Meliceae [7], and Duthieeae [8]), one to three genera were sampled (supplementary table S2, Supplementary Material online). In addition, we sampled the species *Avenula pubescens* with unclear position in Poeae (supplementary table S1, Supplementary Material online). Two species from subfamilies Oryzoideae and Bambusoideae were used as outgroups in phylogenetic reconstruction; in addition, a total of 36 outgroup species were used for molecular dating and WGD inference (supplementary table S1, Supplementary Material online). The transcriptomes of 148 Pooideae and 13 outgroups are newly generated and have an average of 72,138 unigenes and an average N50 of 923 bp per species (supplementary table S3, Supplementary Material online).

### A Well-Resolved Subfamily-Wide Pooideae Phylogeny

**Fig. 1.**
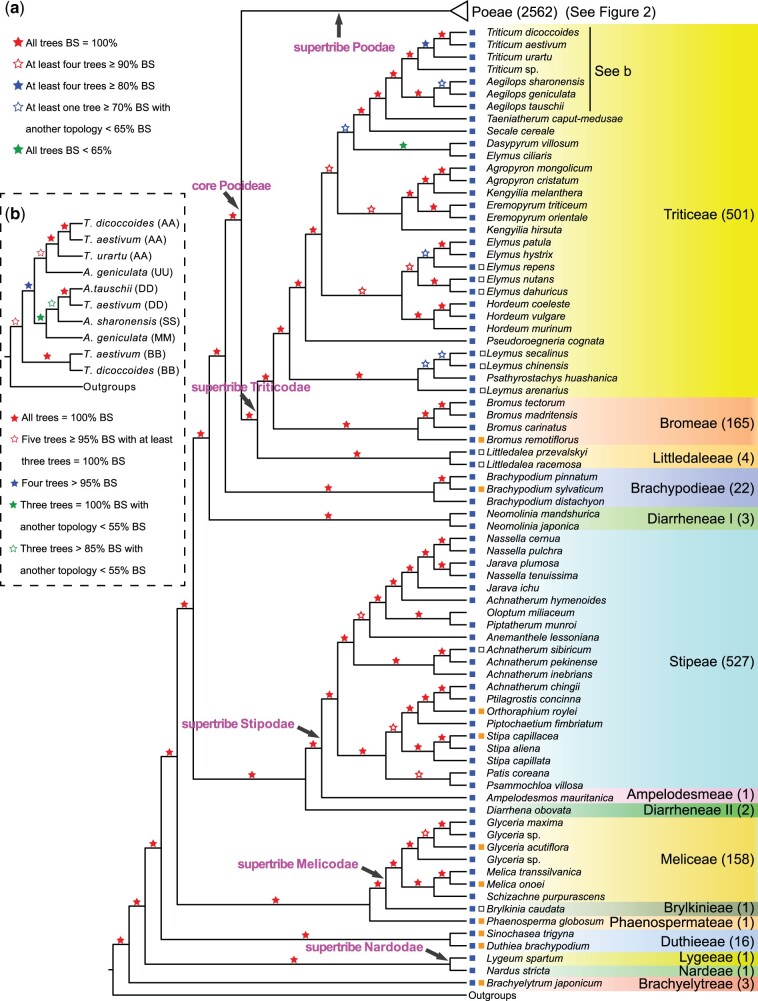
A summarized phylogeny of Pooideae from five trees inferred by ASTRAL. (*a*) The five trees are retrieved from 1,234 OGs, 914 OGs, 763 OGs, 512 OGs, and 373 OGs as described in Materials and Methods. Five levels of BS values are denoted with red (solid or hollow), blue (solid or hollow), and green (solid) stars, respectively. Species belonging to the same tribe are marked with the same background color. The number of species in each tribe is shown in the parentheses beside. Squares on the left of species indicate their climate distribution, temperate (blue), subtropical/tropical (orange), and cold (hollow). Supertribes (pink) defined by Soreng et al. (2017) are indicated on corresponding branches with arrows. (*b*) Relationships of *Triticum*–*Aegilops* complex from five trees (369 OGs, 261 OGs, 158 OGs, 124 OGs, and 81 OGs) inferred by ASTRAL. Five levels of BS values are denoted with red (solid or hollow), blue (solid), and green (solid or hollow), respectively.

**Fig. 2.**
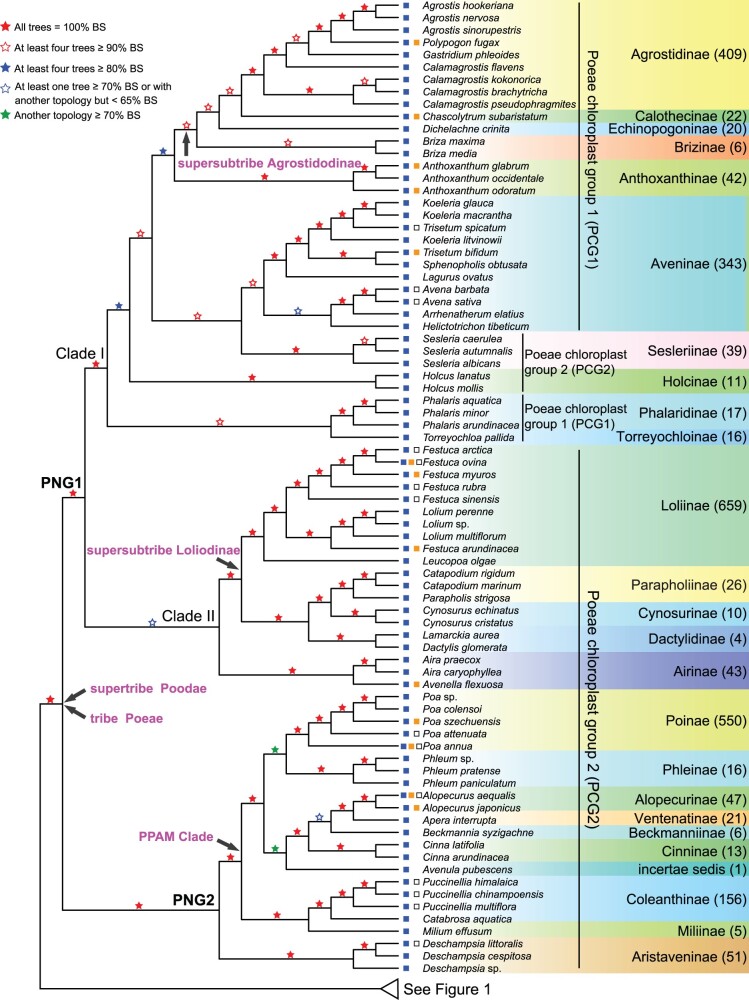
A summarized phylogeny of Pooideae from five trees (Poeae part) inferred by ASTRAL. The five trees and relevant marks are basically the same as figure 1. Four levels of BS values are denoted with red (solid or hollow) and blue (solid or hollow) stars, respectively. Green stars indicate the presence of alternative topologies (see supplementary figs. S5–S9, Supplementary Material online, for more details) among the five trees. The number of species in each subtribe is shown in the parentheses beside. Squares on the left of species indicate their climate distribution, temperate (blue), subtropical/tropical (orange), and cold (hollow). Two major clades defined here, Poeae nuclear group 1 (PNG1) and Poeae nuclear group 2 (PNG2), are marked besides corresponding branches. Supertribe, tribe, and supersubtribes, and PPAM clade defined by Soreng et al. (2017) are indicated on corresponding branches with arrows. Species belonging to Poeae chloroplast group 1 and Poeae chloroplast group 2 defined by Soreng et al. (2017) are marked with vertical bar.

To obtain a robust phylogeny of Pooideae, we selected five gene sets (1,234 orthologous groups [OGs], 914 OGs, 763 OGs, 512 OGs, and 373 OGs) through successive screening by alignment length, species coverage as well as potentially biased signal like long-branch attraction and saturation (supplementary figs. S3 and S4, Supplementary Material online). Then, we reconstructed five trees by a coalescent method, one from each gene set (supplementary figs. S5–S9, Supplementary Material online), generating a highly consistent and robust phylogeny of Pooideae (figs. 1 and 2). The monophyly of Pooideae receives 100% bootstrap (BS) support and is consistent with previous reports (GPWGII 2012; Saarela et al. 2015). All tribes and subtribes with at least two species (eight tribes and 18 subtribes) are also monophyletic with 100% BS except Diarrheneae, which is split into two nonsister lineages (Diarrheneae I and Diarrheneae II; fig. 1 and supplementary figs. S5–S9, Supplementary Material online). Diarrheneae I (*Neomolinia*, distributed in eastern Asia) is sister to Brachypodieae plus the core Pooideae, whereas Diarrheneae II (*Diarrhena*, native in North America) is sister to Stipodae (Stipeae and Ampelodesmeae). The relationships among all tribes are consistent and received 100% BS in all five trees, and those among subtribes of Poeae are also strongly supported, with ≥80% BS in 83% nodes in at least four trees (except Cinninae and *Avenula pubescens*; see below and supplementary note, Supplementary Material online, for details).

#### Relationships at the Tribe Level

In our Pooideae phylogeny, the first two divergent lineages are, respectively, Brachyelytreae and the supertribe Nardodae with the monotypic tribes Lygeae and Nardeae. These placements receive 100% BS in all five trees and agree with previous studies (fig. 1, supplementary figs. S1 and S5–S9, Supplementary Material online). The next divergent lineages include six tribes (Duthieeae, Phaenospermateae, Brylkinieae, Meliceae, Stipeae, and Ampelodesmeae), which previously had inconsistent relationships from analyses using plastid markers (supplementary fig. S1, Supplementary Material online). For example, GPWGII (2012) included five of the six tribes and showed that Ampelodesmeae is nested in Stipeae and the Stipeae/Ampelodesmeae clade is sister to other Pooideae tribes with weak support (43% BS; supplementary fig. S1*a*, Supplementary Material online); also Phaenospermateae and Duthieeae are sisters, with Meliceae being sister to a clade of Brachypodieae + the core Pooideae (GPWGII 2012). In Soreng et al. (2015, 2017) (supplementary fig. S1*b*, Supplementary Material online), the order of the six tribes is Phaenospermateae + Duthieeae (belonging to Phaenospermateae in Soreng et al. [2015]), Meliceae + Brylkinieae, and Stipeae + Ampelodesmeae (no support value). Saarela et al. (2018) sampled four of the six tribes and showed the order of divergences as Phaenospermateae, Meliceae, and Stipeae with Ampelodesmeae embedded, with moderate to high support (supplementary fig. S1*c*, Supplementary Material online). Here, in our phylogeny, the six tribes plus Diarrheneae I and Diarrheneae II are resolved with 100% BS in all trees for all nodes (fig. 1 and supplementary fig. S1*d*, Supplementary Material online). Duthieeae is sister to the other Pooideae tribes, whereas the other five tribes and Diarrheneae II are grouped into two clades: the first with Phaenospermateae and the supertribe Melicodae, and the other with Diarrheneae II and the supertribe Stipodae. In the remainder of Pooideae, Diarrheneae I and Brachypodieae are successive sisters of the core Pooideae. Within Triticeae, Littledaleeae is sister to Triticeae and Bromeae, consistent with previous plastid results (GPWGII 2012; Soreng et al. 2017) with an improvement in coverage and BS support.

The relationships of the Poeae subtribes (fig. 2 and supplementary figs. S5–S10, Supplementary Material online) and wheat relatives are described and discussed in the supplementary note, Supplementary Material online (see supplementary table S1, Supplementary Material online, for sampling and supplementary fig. S11, Supplementary Material online, for gene sets selection; see fig.1*b* and supplementary fig. S12, Supplementary Material online, for results).

### The Divergence Time Estimation for Temperate Grasses

**Fig. 3.**
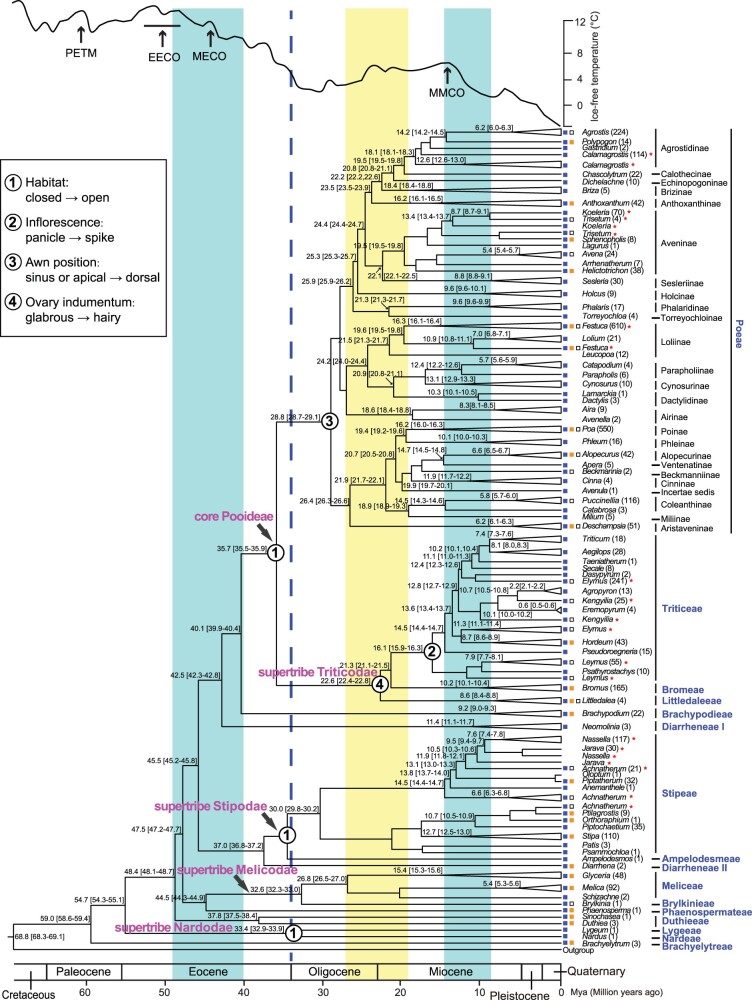
The chronogram and transitions of character state in Pooideae. The Pooideae phylogeny is shown with ages of most clades to the left of the corresponding nodes with mean age and 95% CI (in the square brackets). Names of genera (italic), subtribes (close to genera on the right, only in Poeae), and tribes (blue; on the far right) are shown to the right of branch tips. The number of species in each genus is shown in the parentheses. Squares on the left of genera indicate their climate distribution, temperate (blue), subtropical/tropical (orange), and cold (hollow), stars on the right of genera indicate nonmonophyletic genera. Geological timescale is shown below the time tree. The blue dotted line indicates the rapid reduction of global temperature at the Eocene–Oligocene boundary. The transition of character state is shown with a circle, and the number or letter within it represents the character corresponding to the illustration on the upper left. The blue vertical bands represent the concentrated periods of expansion of tribes and genera, respectively, which correspond to periods with relatively obvious temperature decline. The yellow vertical band represents the concentrated period of expansion of subtribes, with a period of temperate fluctuation after the first cooling period. The global climate curve over the last 65 My (modified from Zachos et al. [2008]) is shown at the top. Time periods of major paleoclimatic events are highlighted: PETM, the Paleocene–Eocene Thermal Maximum; EECO, the Early Eocene Climatic Optimum; MECO, the Mid-Eocene Climatic Optimum; MMCO, the Mid-Miocene Climatic Optimum.

To trace the Pooideae evolutionary history, we performed molecular dating using a penalized-likelihood method implemented in treePL (Smith and O’Meara 2012) with the concatenated data set of 373 OGs. Because of the uncertain placement of the fossil N13 (supplementary table S4, Supplementary Material online) (Prasad et al. 2011), we used three calibration strategies for the estimation with fossil N13 at two different calibration placements, or without the fossil. The estimated times of same node from three calibration strategies show relatively small differences (<3 My, see supplementary figs. S13 and S14 and table S5, Supplementary Material online, for details). For convenience, we describe the result of calibration 1 hereafter unless otherwise stated. As shown in figure 3 and supplementary figure S13, Supplementary Material online, the Pooideae stem lineage was dated to ∼68.8 Ma with a 95% confidence interval (CI) from 68.3 to 69.1; it took ∼20 My for the early Pooideae ancestors to diverge into four extant early divergent clades (namely Brachyelytreae, Nardodae, Duthieeae, and the remaining Pooideae). The molecular dating suggested three periods with concentrated divergences, respectively, of tribes, subtribes, and genera. From the early middle to late Eocene (∼48–39 Ma), several groups diverged within ∼8 My (∼8.8 My including CIs) (fig. 3, blue band on the left), including the MRCA of each of four tribes (Duthieeae: 48.4 Ma, CI: 48.1–48.7; Phaenospermateae: 44.5 Ma, CI: 44.3–44.9; Diarrheneae I: 42.5 Ma, CI: 42.3–42.8; and Brachypodieae: 40.1 Ma, CI: 39.9–40.4), one supertribe (Melicodae: 44.5 Ma, CI: 44.3–44.9), and two other major lineages (Diarrheneae II + Stipodae: 45.5 Ma, CI: 45.2–45.8, and the core Pooideae: 40.1 Ma, CI: 39.9–40.4). Subsequently, the MRCA of each of several large tribes diverged close to the E–O transition, including Meliceae (stem age of 32.6 Ma, CI: 32.3–33.0), Stipeae (34.0 Ma, CI: 34.0–34.0), and Poeae (35.7 Ma, CI: 35.5–35.9). In the late Oligocene to early Miocene (∼26–18 Ma), several groups diverged within ∼7 My (∼7.5 My including CIs) (fig. 3, yellow band), including three tribes of the core Pooideae (Triticeae, Bromeae, and Littledaleeae), and 18 subtribes of Poeae, including three with forage grasses (Dactylidinae: 20.9 Ma, CI: 20.8–21.1; Loliinae: 24.2 Ma, CI: 24.0–24.4; and Poinae: 19.4 Ma, CI: 19.2–19.6) and the economically valuable Aveninae (22.3 Ma, CI: 22.1–22.5). Moreover, most genera (especially those in large tribes Poeae, Triticeae, and Stipeae) diverged within ∼6 My (∼6.7 including CIs) from the middle to late Miocene (fig. 3, blue band on the right). Generally, the ages in this analysis are older than those in previous studies (Vicentini et al. 2008; Bouchenak-Khelladi et al. 2010) but generally similar to recent estimates (Gallaher et al. 2019; Schubert et al. 2019). The inclusion of the new fossils discovered in recent years, such as the fossil of the Poaceae ancestor with an age of >98 Ma (Shi et al. 2012; Wu et al. 2018) might have accounted for the older ages of Pooideae, as suggested by the dating analysis without this fossil (supplementary table S5, Supplementary Material online). Additional information from dating analyses using treePL with different calibration sites (after the removal of one calibration at a time; for details, see Materials and Methods section in the supplementary note, Supplementary Material online) and analyses using the BEAST method are described in the supplementary note, Supplementary Material online (supplementary tables S5 and S6, Supplementary Material online). In general, the results after removal of specific fossil calibrations are quite similar to the those from the treePL analyses using three calibration strategies. In addition, the estimates ages from BEAST (supplementary fig. S15, Supplementary Material online) are generally close to those from treePL analyses, but with larger CI ranges from the BEAST analysis, for relatively late-divergent tribes since the middle to late Eocene, including those of Brachypodieae (43.4 Ma, CI: 39.3–47.1 from BEAST vs. 40.1 Ma, CI: 39.9–40.4 from treePL), Poeae (32.8 Ma, CI: 30.7–34.92 vs. 28.8 Ma, CI: 28.7–29.1), and subtribes such as Loliinae (24.1 Ma, CI: 21.9–26.92 vs. 24.2 Ma, CI: 24.0–24.4), Aveninae (21.3 Ma, CI: 17.9–24.02 vs. 22.3 Ma, CI: 22.1–22.5). On the other hand, the estimated ages of some early divergent lineages showed greater differences, including those for the MRCAs of, respectively, Pooideae (85.6 Ma, CI: 81.5–89.6 from BEAST vs. 68.8 Ma, CI: 68.3–69.1 from treePL), Duthieeae (70.4 Ma, CI: 67.9–73.3 vs. 48.4 Ma, CI: 48.1–48.7), Phaenospermateae + Melicodae (68.7 Ma, CI: 65.0–71.7 vs. 44.5 Ma, CI: 44.3–44.9) (supplementary table S5 and figs. S13 and S14, Supplementary Material online). The greater CI ranges from BEAST analysis also suggest that the divergence times for some tribes (such as Diarrheneae I and Diarrheneae II; supplementary fig. S15, Supplementary Material online) could be older and outside the period of cooling temperature (∼48 to ∼33 Ma, from the early middle to late Eocene). The greater uncertainties in the dating results as suggested by larger CI ranges from BEAST, especially those for early divergences, allow for the possibility of older origins of the Pooideae lineages and could weaken their proposed links to paleoclimates.

### Transitions in Pooideae of Characters Related to the Adaptation to Cool Climates

Using our highly resolved nuclear phylogeny with a timescale (calibration 1) as a reference, we reconstructed the evolutionary histories of six characters with coding information at the genus level (fig. 3 and supplementary figs. S16–S21 and table S7, Supplementary Material online) based on the ML method in the corHMM package (Beaulieu et al. 2013) with the selection of the best-fitting model (supplementary fig. S22, Supplementary Material online).

Environmental factors are external conditions for plants growth and can affect their distribution, diversity, and extinction. Among the six characters, three of them (climate distribution, habitat, and adaptation to water availability) are related to ecological factors. Our results suggest that the MRCA of Pooideae was already distributed in temperate regions (supplementary fig. S16, Supplementary Material online), and the dispersals of Pooideae members to warm (subtropical or tropical) or cold regions might have been more recent events. Our results also suggest that, during much of the global cooling period before the E–O transition, the MRCAs of Pooideae and some of the early divergent lineages might have occupied closed habitats (supplementary fig. S17, Supplementary Material online). Members of three Pooideae lineages transitioned parallelly to open habitats: the MRCA of supertribe Stipodae, the MRCA of the core Pooideae, and the MRCA of Nardeae + Lygeeae (fig. 3 and supplementary fig. S17, Supplementary Material online). Water limitation has been common in temperate regions and the adaptation to different water availability is crucial for the success of plant species. We found that the Pooideae ancestor was likely a mesophyte requiring moderate amounts of water, and that this state has been maintained in most lineages (supplementary fig. S18, Supplementary Material online). Furthermore, there were shifts from mesophyte to xerophyte (requiring little water), including one at the MRCA of Lygeae + Nardeae and the other one at MRCA of most Triticeae genera (at least 11 genera here) including the *Triticum*–*Aegilops* complex, *Secale* and *Hordeum* (supplementary fig. S18, Supplementary Material online).

For the remaining three traits related to spike structure, our results showed that the Pooideae ancestor was likely with a panicle, an apical/sinus awn, and a glabrous ovary (fig. 3 and supplementary figs. S19–S21, Supplementary Material online). These traits then experienced transitions to another state at the MRCA of Triticeae (panicle to spike, supplementary fig. S19, Supplementary Material online), Poeae (sinus/apical to dorsal awn, supplementary fig. S20, Supplementary Material online), and the supertribe Triticodae (glabrous to hairy, supplementary fig. S21, Supplementary Material online), respectively.

In conclusion, these six characters have maintained the ancestral state in most lineages, indicating these traits have experienced largely conservative evolution across Pooideae. Some of the traits have had transitions at the MRCA of lineages, during the global cooling period close to the E–O transition (habitat in supplementary fig. S17, Supplementary Material online) and/or associated with large groups (inflorescence in supplementary fig. S19, Supplementary Material online, awn location in supplementary fig. S20, Supplementary Material online, and ovary indumentum in supplementary fig. S21, Supplementary Material online). These changes in trait states might have contributed to the adaptation of Pooideae to cooling climates and contributed to the formation of large groups. For example, a dorsal awn could have promoted adherence of the spikelet to passing animals, which might have been important for seed dispersal in the relatively open habitats of temperate grasslands; in addition, a fur-like hairy ovary indumentum might have protected the ovary from the damage of low temperatures. Compared with previous studies, some results of our ancestral character reconstruction are consistent, but others are different: the origin of the Pooideae ancestor in temperate region with moderate requirement of water (mesophyte) is generally consistent with the results of Edwards and Smith (2010) and Watcharamongkol et al. (2018). The former inferred that the mean annual temperature was 13–16 °C, and the mean annual precipitation was 1,000–1,500 mm. The latter further inferred a freezing condition in temperate regions. On the other hand, the proposed closed habitat of the Pooideae ancestor is in line with the result of Bouchenak-Khelladi et al. (2010) but not with that of Gallaher et al. (2019).

### Diversification Rate Analyses

**Fig. 4.**
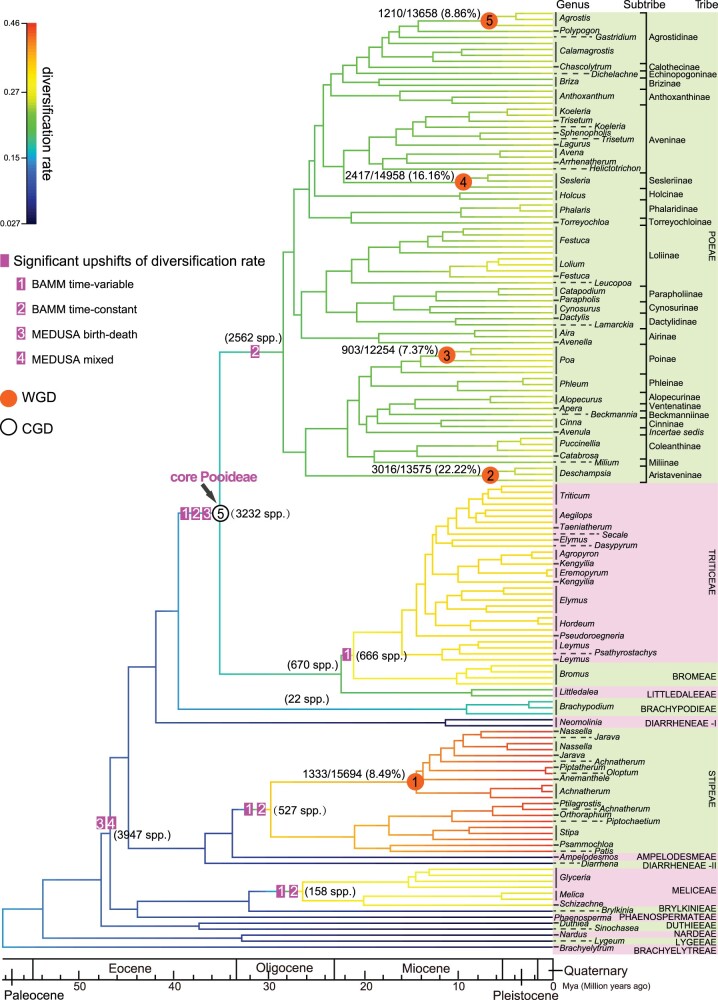
Phylogenetic position of gene/genome duplications and significant upshifts of diversification rate in Pooideae. Pink rectangles are species diversification rate upshifts estimated by BAMM and MEDUSA method, numbers within rectangle represent different models. The number of species in each clade is shown in the parentheses beside each upshift of diversification rate. The branch colors depict the net-diversification rate (in species/million years) obtained from BAMM, corresponding to the color bar on the left. The orange circles denote genome duplications identified from this study (WGD1–WGD5) with the number and percentage of GDs adjacent to each circle. The hollow circle represents a GD burst of the core Pooideae (CGD5). Genera (italic), subtribes, and tribes (uppercase) are marked on the right. The geological timescale is shown at the bottom of the tree.

To examine the history of diversity changes during Pooideae evolution, we used BAMM (Rabosky 2014; Rabosky et al. 2014) and MEDUSA (Alfaro et al. 2009) to infer the shifts of diversification rate. Here, we focused on the shift above the tribe and subtribe levels, rather than at the genus level, because the taxon sample here includes all tribes and >92% (24/26) of subtribes but just over 40% of genera (81/202) (supplementary tables S2 and S8, Supplementary Material online). As shown in figure 4, six upshifts of diversification rate were detected in Pooideae from the two methods. The BAMM analyses detected five upshifts, including one shift close to the MRCA of the core Pooideae (with ∼3/5 of genera and species of Pooideae), and four others at the MRCA of Meliceae, Stipeae, Triticeae plus Bromeae, and Poeae, respectively. Two of the tribes (Poeae and Triticeae) are the largest tribes among the core Pooideae, whereas two other tribes (Meliceae and Stipeae) are the largest outside the core Pooideae. MEDUSA revealed two upshifts: one near the MRCA of the core Pooideae, the same as one of BAMM results, and the other one at the MRCA of Phaenospermateae and Poeae with all Pooideae (including all groups affected by the five upshifts detected by the BAMM analyses), except the four small early divergent tribes. In addition, the increase in diversification rate seems to be linked to the species richness of large tribes or other large groups; this has also been noted in previous studies (Spriggs et al. 2014; Pimentel et al. 2017). For more detailed comparison between this and previous studies, see the supplementary note, Supplementary Material online.

### Gene Duplications and Evidence for Potential Genome Duplication Events in Pooideae

To unravel the evolutionary history of GD across Pooideae and to identify phylogenetically clustered GDs, in part as signals for potential WGD, we applied gene-tree and species-tree (supplementary fig. S23, Supplementary Material online) reconciliation as described before (Yang et al. 2015, 2018) (see details in Materials and Methods and supplementary note, Supplementary Material online). GDs can result from different processes, including WGD, which would lead to a cluster of GDs mapped to a specific node on the species phylogeny. To be conservative in using GD burst as support for candidate WGD, we employed the following two criteria: 1) GD numbers ≥600, GD ratio ≥6% of relevant gene trees; 2) GDs of the ABAB type (i.e., each subclade from the GD contains the species in both two descendent lineages [A and B] from the node) ≥30% (see Materials and Methods). The analyses resulted in 13 GD clusters (supplementary fig. S24 and table S9, Supplementary Material online), including one (CGD1) at the MRCA of Pooideae and those (CGD2–CGD4) at the MRCA of each of three large subclades, three (CGD5–CGD7) at the MRCA of the core Pooideae or associated with the large tribes Poeae and Triticeae, and six others shared by smaller clades in Stipeae, Triciceae, and Poeae.

To test further whether the GD clusters (CGDs) detected here represent signals for WGD, we analyzed genomic synteny (spatial analysis of gene order) (Vision et al. 2000) using relevant genomes and distribution synonymous substitution rate (Ks) for paralogs (Blanc and Wolfe 2004), as previously performed for other WGDs (Vanneste et al. 2013; Jiao et al. 2014; Yuan et al. 2018). Seven of the 13 CGDs (CGD1–CGD7) did not exhibit syntenic signals among affected genomes analyses (supplementary table S9, Supplementary Material online), suggesting that these CGDs were not from WGD. Furthermore, examination of paralogs in sequenced genomes supports the idea that some of these GDs might have resulted from tandem duplication or TE-mediated duplication (supplementary tables S9 and S10, Supplementary Material online). It is worth noting that one CGD (CGD8) was mapped to the MRCA of *Triticum* and *Aegilops*; this is consistent with the hybridization events proposed in previous studies (Marcussen et al. 2014; Li et al. 2015; Glémin et al. 2019) and discussed in the phylogeny part presented in the supplementary note, Supplementary Material online. The remaining five CGDs are further supported by Ks analyses with a peak between 0.13 and 0.14 (supplementary fig. S25, Supplementary Material online), although the corresponding taxa lack sequenced genomes for synteny analyses. These five CGDs support five candidate WGDs (including possible allopolyploidizations following hybridization) in Pooideae (fig. 4 and supplementary figs. S24 and S25, Supplementary Material online), including one shared by six sampled genera of Stipeae (WGD1), and four within Poeae genera: *Deschampsia* (WGD2), *Poa* (WGD3), *Sesleria* (WGD4), and *Agrostis* (WGD5).

For CGD5 (4,527 GDs) at the MRCA of the core Pooideae, which account for >80% of extant Pooideae species richness (fig. 4, hollow circle 5), 385 GDs have paralogs from sequenced genomes, including 152 GDs (39%) and 116 GDs (30%) that might have been generated by tandem duplication and TE-mediated duplication, respectively (supplementary table S9, Supplementary Material online). To obtain clues about possible functions to which the gene duplicates have contributed, we examined the gene ontology (GO) categories of duplicated genes that were detected here for CGD5 and the proposed WGDs. The results show enrichment in secondary metabolic process, pollen–pistil interaction, cell death, and organism process associated with CGD5 and the putative WGDs (supplementary figs. S26 and S27, Supplementary Material online). Several terms associated with reproductive development (such as reproductive system development) are uniquely enriched in the CGD5 paralogs (supplementary fig. S26, Supplementary Material online), suggesting that such functions might have been enhanced in the ancestral core Pooideae. In addition, functions related to signaling pathways are consistently enriched in paralogs from CGD5 and WGDs (supplementary fig. S27, Supplementary Material online), consistent with the previous reports that genes involved in signaling pathway are more likely to be retained after duplication (Freeling 2009; McGrath et al. 2014).

The CGD5 node (fig. 4) is also associated with an upshift in diversification rate and represents >80% species richness of Pooideae (the core Pooideae); thus, we examined the putative functions of duplicates from CGD5 in more detail, with top 24 gene families with most copies listed in supplementary table S11, Supplementary Material online. The annotated functions of these duplicated genes include responses to abiotic stress and biotic stress, and spikelet development. For example, several CGD duplicates encode homologs of the rice OsWAK11 receptor-like protein kinase, which regulates response to toxic heavy metals and inhibits copper uptake (Xia et al. 2018). Among homologs of the CGD5 paralogs are *AtPARK13*, *AtCBF2*, and *CYP71A25*, which are associated with heat and/or cold responses (Basak et al. 2014; Tao et al. 2017; Lin et al. 2019). The CGD paralogs also include homologs of *CRK7* for protection against ozone (O_3_) damage (Idanheimo et al. 2014), of *DCL2* that participates in antiviral silencing response to turnip crinkle virus (Xie et al. 2004), of *AtZAR1* and *UGT76B1* that prevent damage by pathogenic bacteria (Baudin et al. 2017; Holmes et al. 2021), of *MPL1* that acts against green peach aphid (Louis et al. 2010), of *OsMADS1* and *ENL1* involved in regulating spikelet morphogenesis (Hara et al. 2015; Zhang et al. 2018), and of *UBC26* and *AtCBF2* related to abscisic acid (ABA) pathways (Yu et al. 2016; Lin et al. 2019; Fernandez et al. 2020). The increased copy number of these genes due to CGD5 suggests that enhanced stress responses might have contributed to adaptive evolution and expansion of Poeae and Triticodae.

### Statistical Tests for the Correlation between GDs, Character Transitions, and Diversification Rates

To investigate the association between different types of changes from this study, we performed two statistical tests, which have been used in several previous studies (Allio et al. 2021; Lai et al. 2021; Orr et al. 2021). We used the MuSSE test (Multiple State Speciation and Extinction) (Maddison et al. 2007) to investigate whether duplications have had an impact on diversification rates. The GDs were analyzed in three different combinations: five WGDs, eight CGDs, or five WGDs plus eight CGDs, with the selection of the best-fitting model (supplementary fig. S28 and table S12, Supplementary Material online). Overall, the more a lineage has experienced duplications, the higher the net diversification rates (=speciation rate minus extinction rate) (supplementary fig. S28*c*, *f*, and *i*, Supplementary Material online). When considering the five WGDs, the higher net rate has probably resulted from a lower extinction rate (supplementary fig. S28*b*, Supplementary Material online), whereas the addition of CGDs seem to evoke higher speciation rates (supplementary fig. S28*d* and *g*, Supplementary Material online). We also performed a statistical test for the correlation between two of the three types of features (i.e., diversification rate shifts vs. trait transition; trait transition vs. GDs; GDs vs. diversification rate shifts) following Tank et al. (2015) (also see Landis et al. [2018] and Huang, Qi, et al. [2020]) (supplementary fig. S29, Supplementary Material online). As a result (supplementary fig. S29, Supplementary Material online), strong evidence for the correlation (*P* = 0.001–0.01) was identified between any two of the three types of features (supplementary fig. S29*a*–*c*, Supplementary Material online). Separate analyses of correlations between the transition of a specific morphological trait with the diversification rate shifts or GDs indicated moderate evidence between diversification rate shifts and transitions of awn position (*P* = 0.019) (supplementary fig. S29*d*, Supplementary Material online) and also between GDs and transitions of awn position (*P* = 0.042) or inflorescence (*P* = 0.041) (supplementary fig. S29*h* and *j*, Supplementary Material online). These results provide support for the hypothesis that GDs (including CGD and WGD), at least in part, have had a positive impact on functional innovation and diversification, thereby enhancing the fitness and adaptation of Pooideae (see more details in the second paragraph of Discussion).

### Molecular Evolution of *AP1*/*FUL*-Like Subfamily of MADS-Box Genes

One of the characteristics of the temperate climate is seasonal change, especially the prolonged low temperature through the winter. For temperate cereals and other temperate plants, the treatment with long exposure to low (winter-like) temperature is called vernalization, which leads to the flowering transition from vegetative growth to reproduction. Vernalization is required for flowering of temperate grasses and others similarly adapted to regions with cold winters, but not for flowering in (sub)tropical grasses such as rice; hence vernalization-dependent flowering was thought to be a newly evolved process in temperate lineages (Li et al. 2016; McKeown et al. 2016). Members of the *APETALA1/FRUITFULL-*like (*AP1*/*FUL-*like) subfamily of MADS-box genes play roles in response to vernalization in cereals (such as *VRN1* in wheat) (Yan et al. 2003, 2004; Trevaskis 2010; Kippes et al. 2015) and in inflorescence and floral meristem identities in rice (*FUL1*/*OsMADS14*, *FUL2*/*OsMADS15*, *FUL3*/*OsMADS18*, and *FUL4*/*OsMADS20*) (Wu et al. 2017). Duplications of *AP1*/*FUL-*like genes in Poaceae were previously reported (Preston and Kellogg 2006; Wu et al. 2017), but the phylogenetic positions of duplications are largely unclear because of limited taxon sampling.

**Fig. 5.**
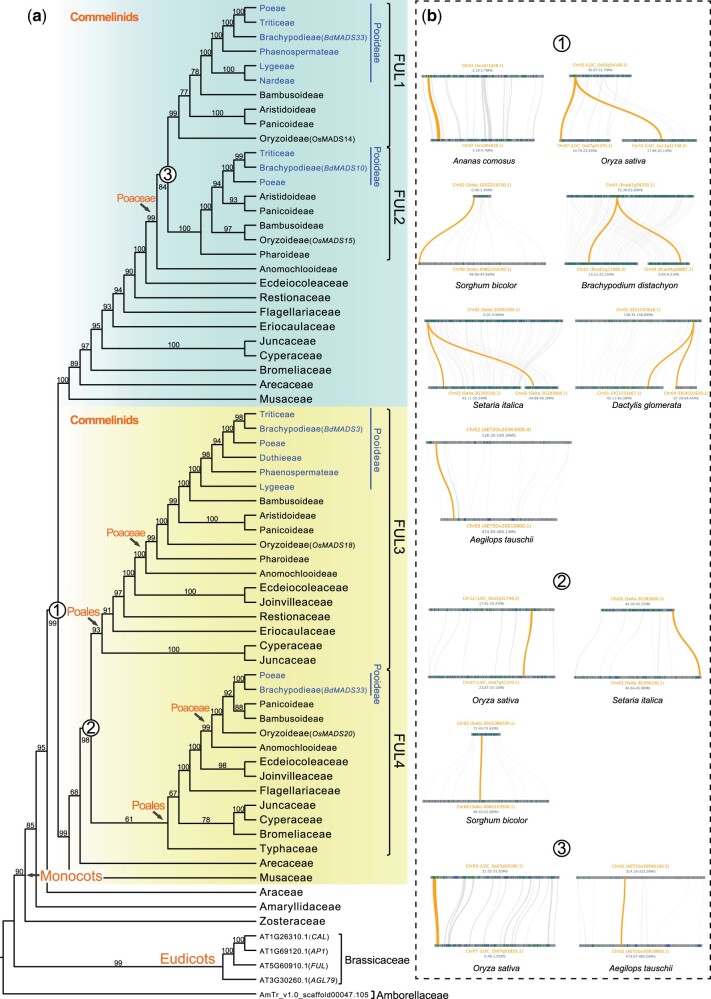
Molecular evolution of *APL/FUL*-like genes. (*a*) The phylogenetic tree of *AP1*/*FUL*-like genes. Hollow circles show duplication events. BS values are presented on the branches. Clades of Pooideae are colored with blue. Genes from *Arabidopsis thaliana*, *Oryza sativa*, and *Brachypodium distachyon* are marked in the parentheses. (*b*) The location of syntenic blocks of *AP1*/*FUL* paralogs (yellow) from genomes is presented with gene ID in the parentheses.

Here, to examine the evolution of *AP1*/*FUL*-like subfamily in grasses, especially in Pooideae, and to position the GD event(s), we selected 26 Poaceae, 15 other Poales, and seven outgroups (supplementary table S13, Supplementary Material online) to reconstruct the gene family tree. The results here (fig. 5*a* and supplementary fig. S30, Supplementary Material online) indicate four grass-specific clades of *AP1*/*FUL*-like genes, consistent with the previously defined *FUL1*–*FUL4* clades (Wu et al. 2017). Further analysis of the genomic collinearity in grass genomes indicates that the duplicated paralogs are mostly located in syntenic blocks (fig. 5*b*), suggesting that the four *FUL* clades are a result of genome duplications. The grass family was reported to experience three polyploidizations referred to as tau (*τ*) and sigma (*σ*) at the MRCA of commelinid monocots and Poales, respectively, and rho (*ρ*) associated with the early Poaceae history (Paterson et al. 2004, 2009; D’Hont et al. 2012; Jiao et al. 2014; Ming et al. 2015). Here, we propose that the *τ*, *σ*, and *ρ* WGDs together generated the four *FUL* clades: 1) the oldest duplication leading to *FUL1*–*FUL2* (blue) and *FUL3*–*FUL4* (yellow) occurred at the MRCA of commelinids including Poales, Zingiberales, and Arecales (circle 1, fig. 5*a*), corresponding to the position of *τ*; 2) the event giving rise to *FUL3* and *FUL4* is across all members of Poales (circle 2) coincides with *σ*; and 3) the duplication generating *FUL1* and *FUL2* (circle 3) affects most Poaceae subfamilies, except the early divergent subfamily Anomochlooideae, very close to *ρ*, whose precise position is still unclear.

Previous studies in several temperate grasses (e.g., *Brachypodium distachyon*, wheat, barley) found that *FUL1*, *FUL2*, and *FUL3* homologs are key regulators of response to vernalization (Ream et al. 2012; Li et al. 2016; McKeown et al. 2016). Here, we found that both *FUL1* and *FUL3* homologs have been retained in most Pooideae lineages, especially in the representatives from the early divergent tribes (Nardeae, Lygeeae, Duthieeae, and Phaenospermateae); these tribes were estimated to diverge during the cooling period in the Eocene (fig. 3), suggesting the possibility that the early Pooideae might already have had the potential to adapt to seasonal climate by vernalization. However, *FUL4* homologs were only found in Poeae members (transcriptome data) and *B. distachyon* but not in other Pooideae. The expression levels of the *FUL4* ortholog in the rice (*OsMADS20*) and *B. distachyon* (*BdMADS31*) are very low (Li et al. 2016; Wu et al. 2017), suggesting that the nondetection of *FUL4* homologs in our transcriptome data sets might be due to their low expression levels. However, the absence of *FUL4* homologs even in genome sequences here suggests that *FUL4* might not be required for vernalization and might have undergone pseudogenization, consistent with the previous result that the FUL-like motif was lost in the *B. distachyon FUL4* homolog (Li et al. 2016).

### Multiple Tandem Duplications of *CBF* Genes at Different Stages of Pooideae Evolution

C-repeat binding factors (CBF) are transcription factors that belong to the APETALA2/Ethylene Responsive Factor (AP2/ERF) protein superfamily. CBF can bind to the C-repeat/dehydration responsive (CRT/DRE) element of Cold-regulated (COR) and Dehydrin (DHN) genes and enhance the tolerance to low temperature, drought, and salt stresses (Stockinger et al. 1997; Haake et al. 2002; Nakashima et al. 2009). The numbers of *CBF* genes vary greatly across different lineages: from six in *Arabidopsis thaliana* and ten in rice (Li et al. 2020) to 20 in barley (Skinner et al. 2005) and 37 in wheat (Guo et al. 2019). The copy number variation of *CBF* in barley population was found to associate with the level of freezing tolerance (Knox et al. 2010; Würschum et al. 2017). In addition, Sandve and Fjellheim (2010) suggested that the amplifications of *CBF* and *FT* genes might have played a role in helping the core Pooideae ancestor adapt to the super-cooling climate during the E–O transition, although this proposal was based on an analysis using only eight core Pooideae species. The recent advances in genome sequencing of Pooideae (Huang et al. 2020; Wang et al. 2020; Zhang, Liu, et al. 2020) provide a great opportunity to discern the evolutionary pattern of *CBF* copy number across temperate grasses, as clues to the molecular basis of adaptation to cold climates.

**Fig. 6.**
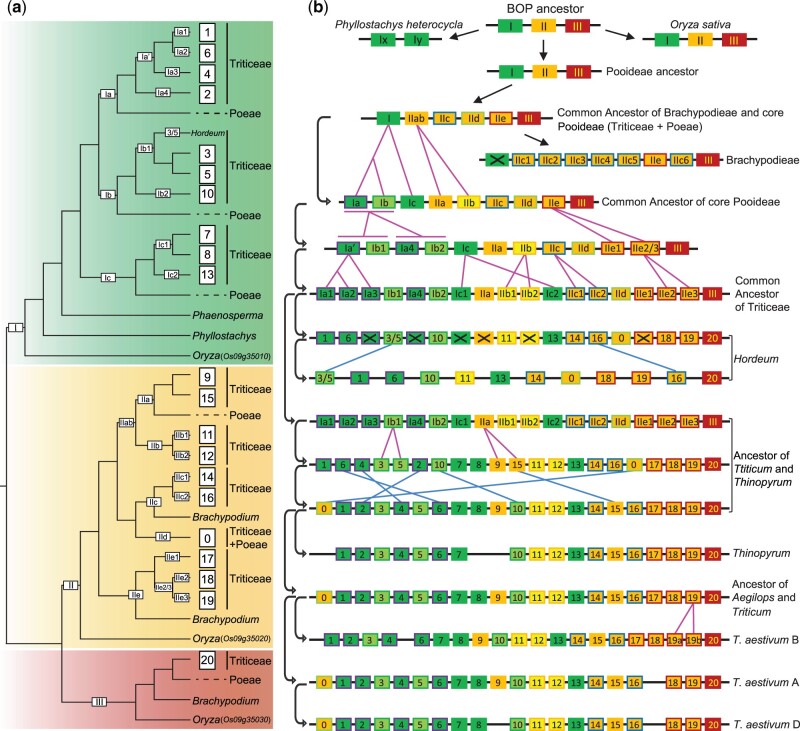
Molecular evolution of *CBF* genes. (*a*) A summarized gene tree of *CBF* genes. Three duplicate clades from Bambusoideae, Oryzoideae, and Pooideae (BOP) are colored with green, yellow, and red, respectively. Arabic numerals on the branch indicate GD, and the box with the numbers 0–20 represents a clade (orthologs group) that derived from the last GD. Genes from the *Oryza sativa* are indicated in the parentheses. (*b*) An evolutionary trajectory of *CBF* genes. Three duplicates from BOP corresponding to (*a*) are indicated with green, yellow, and red boxes, respectively. Box with numbers or Arabic numerals are consistent with (*a*), and the box with X indicates gene loss. Blue and pink lines indicate gene rearrangement and GD, respectively. The curve connects the ancestor and descendant (arrow side) of *CBF*.

Here, we performed phylogenetic analysis of *CBF2A*, which promotes COR transcription and enhances freezing tolerance (Jeknic et al. 2014), and its homologs from 15 grass species and two outgroups (supplementary table S14, Supplementary Material online). The resulting gene tree (fig. 6 and supplementary fig. S31, Supplementary Material online) includes three well-resolved clades (termed as clade I, II, and III, respectively) each containing genes of Oryzoideae and Pooideae members, suggesting two GDs at the MRCA of the BOP subfamilies: Bambusoideae, Oryzoideae, and Pooideae. Intriguingly, further GDs occurred several times at the MRCA of the core Pooideae + Brachypodieae, the core Pooideae, or Triticeae, respectively (fig. 6 and supplementary fig. S31, Supplementary Material online). For example, clade I contains three subclades (Ia, Ib, and Ic) with members of Poeae and Triticeae, whereas clade II has four subclades (IIab, IIc, IId, and IIe) that likely originated at the MRCA of Brachypodieae and the core Pooideae. In short, this expansion generated high copy numbers of *CBF* in the representatives of Triticeae (i.e., *T.aestivum* [A subgenome: 20 copies; B subgenome: 19 copies; D subgenome: 19 copies], *T. dicoccoides* [A: 14; B: 6], *Aegilops tauschii* [17], and *Thinopyrum elongatum* [18]), Poeae (*Dactylis glomerata* [18], *Puccinellia tenuiflora* [6]), and Brachypodieae (*B.distachyon* [8]). In contrast, the tropical/subtropical rice and bamboo and their relatives have low copy numbers of *CBF* genes, suggesting that increased *CBF* copy numbers might be associated with the plant distribution to regions of temperate climates, as proposed before for temperate cereals (Knox et al. 2010).

To gain further insights into the evolution of *CBF* genes in Triticeae, we examined the chromosomal location of *CBF* duplicates and found that they are located in a gene cluster on the same chromosome, with fewer than three non-*CBF* genes between any two closest *CBF* genes (supplementary fig. S32, Supplementary Material online). Specifically, *CBF* genes in Triticeae, Poeae, and Brachypodieae genomes are in tandem arrays on chromosome 5, 3, and 4, respectively. Even the relatively few rice *CBF* genes also show a tandem arrangement on chromosome 9. The genomic positions and the phylogeny (fig. 6*a* and supplementary fig. S32, Supplementary Material online) both support the view that tandem duplication has been the main mechanism for generating multiple copies of *CBF* genes in Pooideae grasses, with duplications at different stages of the Pooideae history (see fig. 6*b* for an illustration of the model with an emphasis for genes in Triticeaee; the genes marked with green, yellow, and red colors matching those in the *CBF* phylogeny are shown in supplementary fig. S31, Supplementary Material online, right). In the model (fig. 6*b*), the BOP ancestor had three CBFs (represented by I, II, and III), which are maintained in rice; in addition, *CBF*-I was duplicated (*CBF*-Ix and *CBF*-Iy) but *CBF*-II and *CBF*-III were likely lost in *Phyllostachys heterocycla* (Bambusoideae). Furthermore, *CBF*-II experienced significant expansion in Pooideae (fig. 6 and supplementary fig. S31, Supplementary Material online), with some lineage-specific duplication of *CBF*-III (supplementary fig. S31, Supplementary Material online), resulting in at least six copies (including four *CBF*-II copies) in the ancestor of Brachypodieae and the core Pooideae. Further duplications of *CBF*-IIc in Brachypodieae increased *CBF*-IIc to six copies, with maintenance of *CBF*-IIe and *CBF*-III and loss of *CBF*-I, *CBF*-IIab, and *CBF*-IId. In the ancestor of the core Pooideae, *CBF*-I and *CBF*-II copy numbers increased to three and five copies, respectively. The expansion of *CBF* genes continued in Triticeae, ultimately resulting in 18 copies in the Triticeae ancestor and ∼20 copies in each of the three wheat subgenomes and related species, such as *T. aestivum*, *T. dicoccoides*, *Aegilops tauschii*, and *Thinopyrumelongatum* (fig. 6*b* and supplementary fig. S31, Supplementary Material online).

Following duplication, functionally redundant genes are often lost (Lynch and Conery 2000). The analyses here revealed that *CBF* duplicates have experienced lineage-specific losses. For example, barley likely lost six copies (boxes with X in fig. 6*b*) from the ancestor of Triticeae, whereas wheat (including three subgenomes) and its relatives show different patterns of gene loss (gaps in gene arrays corresponding to missing numbers) across species/chromosomes, suggesting differential evolution of *CBF* in Pooideae especially within Triticeae. The complex *CBF* evolutionary pattern is also reflected by multiple gene rearrangements in Triticeae (blue lines in fig. 6*b*). Altogether, *CBF* genes in temperate grasses have gone through stepwise expansion along with different levels of gene loss and rearrangement, possibly implying that an integrated regulatory network in response to chilling has evolved progressively during Pooideae history from a more limited ancestral regulatory machinery in the BOP ancestor.

## Discussion

Here, we used over 1,200 nuclear genes from 157 transcriptomes to reconstruct a highly resolved Pooideae phylogeny, with strongly supported monophyly for 14 of 15 tribes and all subtribes with two or more sampled taxa, but polyphyly of Diarrheneae. Furthermore, using the well-resolved phylogenetic relationships among Pooideae lineages, we estimated their origins and divergence times, reconstructed the evolutionary history of several traits, investigated diversification dynamics, detected numerous duplications, and analyzed molecular evolution of *FUL* and *CBF* homologs, providing clues to the success of Pooideae in adaptation to temperate environments.

### Pooideae Nuclear Phylogeny Reveals Deep Cytonuclear Discordances and Suggests Possible Hybridization

In previous plastid phylogenies, the relationships were uncertain for several early divergent Pooideae tribes (e.g., Duthieeae, Phaenospermateae, Stipeae, and Meliceae) and for Poeae subtribes (Schneider et al. 2009, 2011; GPWGII 2012; Soreng et al. 2017). The well-resolved phylogeny here using many more nuclear gene than possible with plastid genes reveals the order for the early divergent tribes (fig. 1, supplementary figs. S1 and S5–S9, Supplementary Material online) and groups the Poeae subtribes into two new clades: Poeae nuclear group 1 (PNG1) and Poeae nuclear group 2 (PNG2) (fig. 2 and supplementary figs. S2 and S5–S9, Supplementary Material online) instead of the two recognized from plastid evidence previously. PNG1 includes all eight subtribes of PCG1 plus seven subtribes of PCG2, whereas PNG2 contains the remaining nine subtribes of PCG2. The discordances between nuclear and plastid phylogenies imply a complex history across Pooideae, with possible reticulation events and/or incomplete lineage sorting, as described/discussed below and in the supplementary note, Supplementary Material online.

Diarrheneae has five species in two genera with distribution in eastern Asia (*Neomolinia*, three species) and North America (*Diarrhena*, two species) (Soreng et al. 2015, 2017); this classification was further supported by morphological and molecular evidence (Tzvelev 1989; Schneider et al. 2011). In a study of Hochbach et al. (2015), phylogenetic analyses of 53 Pooideae (including both *Diarrhena* species and one *Neomolinia* species) with one plastid and four nuclear sequences showed that the monophyly of the Diarrheneae tribe was supported by the sisterhood of the two genera with 63–100% BS from two nuclear sequences (*PhyB*, *Topo6* exon 17–19), whereas the results using the plastid and two other nuclear sequences (*Acc1*, *Topo6* exon 8–13) failed to group *Diarrhena* and *Neomolinia* together (Hochbach et al. 2015). In this study, *Diarrhena* is maximally supported as sister to Stipeae + Ampelodesmeae, whereas *Neomolinia* is sister Brachypodieae + core Pooideae. We further examined all 1,234 nuclear gene trees and found the polyphyly of Diarrheneae in 71% of trees. Therefore, a taxonomic revision of Diarrheneae would be plausible. The discordance between nuclear and plastid possibly implies that the ancestor of *Diarrhena* might have involved an ancient hybridization between *Neomolinia* and the MRCA of the supertribe Stipodae; this hypothesis is consistent with the observation that a large fraction of nuclear genes supports a close relationship to Stipodae, whereas in plastid phylogenies and those of a small portion of nuclear genes *Diarrhena* is close to *Neomolinia*. Another possible explanation for the discordance between some of the gene phylogenies and the species topology is incomplete lineage sorting. In addition, the placement here of Phaenospermateae as sister to the supertribe Melicodae (Meliceae + Brylkinieae) with 100% BS in all five trees is in agreement with previous analyses using nuclear sequences (79–91% BS) (Hochbach et al. 2015). However, in plastid studies Phaenospermateae was consistently grouped with Duthieeae with 75–95% BS (Schneider et al. 2009; Hochbach et al. 2015).

Previous phylogenetic analyses of subtribes within Poeae using plastid sequences often resulted in two clades, PCG1 and PCG2, but with sparse sampling at the subtribe level. On the other hand, analyses using nuclear sequences revealed that some subtribes of PCG2 (usually Airinae, Holcinae, Sesleriinae, Scolochoinae) were nested in PCG1 (Schneider et al. 2009; Tkach et al. 2020), but with uncertain relationships. Here, our phylogenetic trees consistently resolved the subtribes into two new clades (PNG1–PNG2, fig. 2 and supplementary figs. S2 and S5–S9, Supplementary Material online), with the subtribes Holcinae and Sesleriinae being nested among the subtribes of the previously defined PCG1, in partial agreement to the previous results from nuclear sequences. An examination of the concordance numbers of the 1,234 single genes trees to the alternative hypotheses ([PNG1 and PNG2] or [PCG1 and PCG2]) of the Poeae phylogeny shows higher fractions with concordance to the PNG1+PNG2 topology (supplementary fig. S33, Supplementary Material online, for details, see the supplementary note, Supplementary Material online). In addition, we evaluated the branch support by ASTRAL-pro with the function of “newick annotations” (Zhang, Scornavacca, et al. 2020), with estimation for both the quartet support (QS) and local posterior probability (LPP) for each branch. The QS represents the percentage of quartets in gene trees that agree with branch topology, whereas the LPP is computed using a transformation of the QS, with a probability value of 0.33 considered sufficient support for a branch and higher score indicating more consistent single gene trees. Our analyses of the single trees of five OGs uncovered scores of LPP close to the maximum values, and the scores of QS >0.5 (supplementary figs. S34–S43, Supplementary Material online, for details, see the supplementary note, Supplementary Material online), supporting the topology of PNG1 and PNG2 clades. The phylogeny reconstructed by multicopy genes also indicated high LPP for the PNG1 and PNG2 clades of Poeae (supplementary figs. S44–S47, Supplementary Material online, for details, see the supplementary note, Supplementary Material online). These results all support the nuclear two-clade topology over the chloroplast two-clade topology.

### GD via Different Mechanisms Likely Contributed to Pooideae Adaptation to Open Habitat

**Fig. 7.**
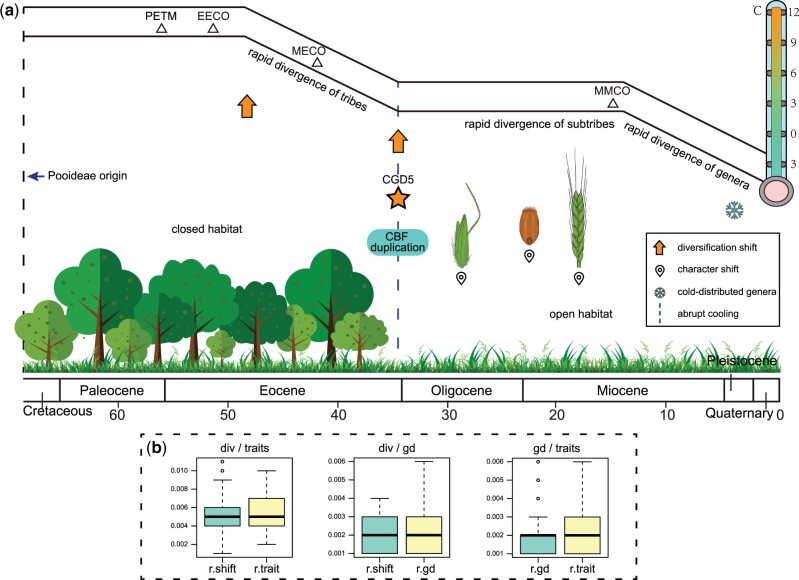
A brief illustration of proposed Pooideae evolutionary history. (*a*) A simplified temperature curve corresponding to the geological timescale adopted from Zachos et al. (2008) is shown at the top with major paleoclimate events marked by the triangles. PETM, the Paleocene–Eocene Thermal Maximum; EECO, the Early Eocene Climatic Optimum; MECO, the Mid-Eocene Climatic Optimum; MMCO, the Mid-Miocene Climatic Optimum. Three concentrated periods of expansion at the levels of, respectively, tribe, subtribe, and genus are marked beside the simplified temperature curve (corresponding to fig. 3). The rapidly cooling event (blue dotted line) at the E–O transition associated with the upshift of diversification rate (yellow arrow), CGD5 (star), duplication of *CBF*, and the transition from closed to open habitat at the MRCA of the core Pooideae are displayed. Character state shifts of inflorescence (at the MRCA of Triticeae, fig. 3 and supplementary fig. S19, Supplementary Material online), awn location (at the MRCA of Poeae, fig. 3 and supplementary fig. S20, Supplementary Material online), and ovary indumentum (at the MRCA of Triticeae+Bromeae+Littledaleeae, fig. 3 and supplementary fig. S21, Supplementary Material online) are also illustrated. Recent origins of cold-distributed genera are indicated with a snowflake logo according to figure 3 (hollow square beside the genera). (*b*) Statistical tests for the correlation of GDs, diversification upshifts, and transition of traits. The two features in each analysis are noted at top of each panel. Boxplots show the quantiles of *P* values from 100 repeats (see Materials and Methods). r.shift, analysis compared with the null hypothesis using randomly placed nodes of rate shift; r.trait, analysis compared with the null hypothesis using randomly placed nodes of trait transition; r.gd, analysis compared with the null hypothesis using randomly placed GD nodes (see supplementary fig. S29, Supplementary Material online, for more details).

Among the CGDs identified here, CGD5 (circle five, fig. 4) at the MRCA of the core Pooideae includes over 4,500 GDs and coincides with a transition from a closed habitat to an open one (figs. 3 and 7), with subsequent changes in characters (figs. 3 and 7). Among the duplicated genes in CGD5, members of gene families involved in stress response (e.g., *CBF*, *OsWAK11*, *CYP7, DCL2, MPL1*) and reproductive development (e.g., *OsMADS1*, *ENL1*) were particularly retained (supplementary table S11, Supplementary Material online). Specifically, molecular phylogenetic analyses of *CBF* homologs (fig. 6 and supplementary fig. S31, Supplementary Material online) detected three duplications at this node. Innovations in the architecture of spikelet, including dorsal awns (at the MRCA of Poeae), hairy ovaries (at the MRCA of Triticodae), and inflorescence (at the MRCA of Triticeae) (figs. 3 and 7; supplementary figs. S19–S21, Supplementary Material online) occurred after CGD5. The awn increases the chance that the spikelet is caught on the fur of passing animals (Elbaum et al. 2007), hence facilitating long-distance seed dispersal; the awn can also enhance the penetration of spikelet/seed into the soil (Elbaum et al. 2007), potentially contributing to drought tolerance (Peleg et al. 2010). The transition of the awn location from sinus or apical to dorsal might have led to higher tolerance to environmental stresses associated with open habitat, such as drought, and also promoted seed dispersal. Another change involves the ovary indumentum, from glabrous to hairy. The innovation with hairs might be associated with the resistance to water deficit and insect resistance at the MRCA of Triticodae. These changes (including molecular and phenotypic characters) could have been helpful for plant adaptation to open habitat. The correlation between GD and trait transition is statistically supported (fig. 7 and supplementary fig. S29, Supplementary Material online), implying that GDs might have played a role in character transitions. Therefore, the increased genetic materials and associated variation due to the CGD5 might have led to molecular changes such as greater gene functions for cold-response and key shifts in morphology (fig. 7*b*), and the physiological and developmental innovations then contributed to plant adaptation to new or stressful environments. In addition, it is possible that other factor(s) might have driven both the retention of duplicated genes and morphological changes. Moreover, the open habitat might have subsequently facilitated the radiation and expansion of Pooideae (fig. 7*b*), such as the rapid radiation of subtribes in the core Pooideae in the late Oligocene to early Miocene (fig. 3 and supplementary figs. S13 and S14 and table S5, Supplementary Material online). This scenario explains, at least in part, how the Pooideae “migrated” out of the canopy environment of forests to open habitats and became a highly successful lineage in the temperate flora.

Several studies suggested that GDs, especially those of WGD, have an impact on diversification (Tank et al. 2015; Landis et al. 2018). Here, we used two different methods (MuSSE and the test proposed by Tank et al. 2015) to test this hypothesis. The results support the idea that the lineages that have experienced more duplications or retained more gene duplicates often exhibit higher diversification rates (supplementary fig. S28, Supplementary Material online). Other alternative hypotheses are still possible; for example, some lineages might have been through a period of small population sizes, possibly due to relatively high diversification rates, and experienced fixation of some gene duplicates that are not necessarily functionally related to diversification. In addition, the correlation between GDs and diversification rate upshifts is statistically supported (fig. 7*b* and supplementary fig. S29, Supplementary Material online). For instance, over 4,500 GDs of CGD5 that is linked to the large clade of the core Pooideae (Poeae + Triticodae) with over 80% of Pooideae species. Not surprisingly, we detected a significant upshift of diversification at the same node/stem (figs. 4 and 7). In addition, we found evidence for WGDs associated with several genera (figs. 3 and 4) including the large genera *Poa* (∼550 spp.) and *Agrostis* (∼224 spp.), implying that WGD might have also contributed to species richness of groups with relatively recent origins. From these results, we propose that GDs (including CGD and WGD), at least in part, have had a positive impact on functional innovation and diversification (fig. 7*b*), thereby enhancing the adaptation of Pooideae, although other hypotheses are also possible.

### Climate Change Might Have Been a Driver for the Adaptive Evolution in Pooideae

Climate has had profound impacts on the origin, distribution, diversity, and even extinction of organisms (Jacobs et al. 1999; Hooker et al. 2004; Svenning et al. 2015). Since the Cretaceous, the Earth has experienced several climate upheavals including the hot periods of the Paleocene–Eocene Thermal Maximum (PETM), the Early Eocene Climatic Optimum (EECO), and the Mid-Eocene Climatic Optimum (MECO), as well as the abrupt cooling during the E–O transition (Zachos et al. 2008); especially, the E–O transition was the beginning of the last Cenozoic ice age (Kvasov and Verbitsky 1981; Goldner et al. 2014) and was suggested to have greatly affected the origin and expansion of temperate biomes (Favre et al. 2016; Meseguer et al. 2018).

Pooideae have been a prominent part of temperate ecosystems and their diversification was suggested to be driven by paleoclimate under global cooling (Schubert et al. 2019). Our results suggest that the cooling climates have been correlated with GD, diversification, and radiation across Pooideae. In addition, the origin of Pooideae and those of early divergent lineages were in closed habitats (supplementary fig. S17, Supplementary Material online) from the late Cretaceous to the late Eocene (∼68–35 Ma), with relatively warm climates including the PETM and EECO and a long subsequent cooling period (figs. 3 and 7). After the EECO, there was an overall cooling trend except the abrupt warming at the MECO, with further sudden reduction of the global temperature at the E–O transition (Zachos et al. 2008). The global cooling led to the contraction of tropical forests to low latitudes (Prothero 1994; Donoghue and Edwards 2014), resulting in many new/open habitats. The changed climate and newly available habitats presented both challenges and opportunities for Pooideae plants; indeed, Pooideae experienced successive divergences of early divergent lineages (tribes) (figs. 3 and 7) and an upshift of diversification rate (figs. 3 and 7) during this cooling period, suggesting that the changing climate might have been a contributing factor to Pooideae divergence. This is also close to the period when GDs (CGD5) and the transition to open habitat occurred in the ancestor of the core Pooideae (figs. 3, 4, and 7). The CGD5 duplicates included *CBF* genes (figs. 6 and 7; supplementary fig. S31 and table S11, Supplementary Material online) and the increased *CBF* copies possibly enhanced the ability of Pooideae species to tolerate freezing during global cooling especially the E–O transition in open habitats. At the same time, some genes associated with adaptation to seasonal cooling might have already existed in the ancestor of Pooideae, as is the case of the vernalization-related *AP1*/*FUL*-like genes, which were duplicated due to WGD shared by all grasses and have been retained in the early divergent tribes (fig. 5 and supplementary fig. S30, Supplementary Material online). Another example is the *CBF* copies that were already present at the MRCA of Brachypodieae and the core Pooideae (fig. 6 and supplementary fig. S31, Supplementary Material online). Hence, early Pooideae might already have the potential to adapt to the temperate regions before the E–O transition, which in turn could explain the survival of early Pooideae during gradual temperature decline from the late Eocene to the E–O transition.

In the late Oligocene to early Miocene, the frequent continental plate movement and orogeny continued to create new habitats, and also resulted in seasonal climates and increased aridity. This period also witnessed the radiation of most Poeae subtribes (figs. 3 and 7; supplementary figs. S13–S15 and table S5, Supplementary Material online) and divergence of other species-rich tribes (e.g., Triticeae and Stipeae). After the Mid Miocene Climatic Optimum (MMCO), there was another rapid cooling period to a lower temperature than ever before; this was also a period of multiple divergence events for many Pooideae genera (figs. 3 and 7; supplementary figs. S13–S15 and table S5, Supplementary Material online). Furthermore, the WGD events within genera (WGD2–WGD5, fig. 4) were estimated to have occurred during this aridity/cooling period, suggesting that both the WGD and climate might have accelerated speciation and radiation in Pooideae. In addition, recent dramatic cooling since ∼7 Ma corresponded to the appearance of species adapted to cold habitats in multiple genera (fig. 7 and supplementary figs. S13–S15 and table S5, Supplementary Material online), suggesting that cooling/cold climates might again have been an important external factor for species evolution and dispersal. Notably, the dating results can be impacted by the use of different gene sequences, phylogenies, estimation methods, fossils, and their calibrations; therefore, we need to be cautious about the ages of divergences of Pooideae lineages and the related hypothesis for links to global temperature changes. Even though the dating results using treePL here were quite consistent among the analyses here, it is possible that substantially different ages for the Pooideae lineages might be obtained using other methods and/or gene sequences, leading to alternative explanation for Pooideae cold adaptation.

The coincidence of expansion of Pooideae with cooling climate has also been suggested in previous studies (Jacobs et al. 1999; Strömberg 2011; Schubert et al. 2019). Our results here further demonstrate that in the history of Pooideae, environmental changes/stresses (such as climate change or a new type of habitat) might magnify the advantages provided by gene/genome duplications, which increased genetic materials and functional divergence and supported innovation of key characters as suggested previously (Soltis and Soltis 2016; Van de Peer et al. 2017). The combination of internal genetic events (gene/genome duplication) and external environmental stimuli (climate change) resulted in the radiation of Pooideae into multiple successful lineages with wide expansion to new territories in temperate regions.

## Materials and Methods

More detailed methods are described in the supplementary note, Supplementary Material online.

### Taxon Sampling, Sequencing, and Assembly

To reconstruct a phylogenetic tree for Pooideae, we strived to sample taxa represent all 15 tribes and nearly all subtribes (24 of 26) in Poeae, the largest Pooideae tribe. Overall, a total of 157 species were sampled in Pooideae as ingroups, and 38 other species as outgroups across Poales, Zingiberales, Arecales, Asparagales, and eudicots (Ranunculales, Brassicales, and Vitales) (supplementary table S1, Supplementary Material online). Among the sampled species, 161 are newly sequenced (supplementary table S3, Supplementary Material online) for transcriptome sequencing. Raw reads were assembled by Trinity v2.9 (Grabherr et al. 2011) with default settings. TransDecoder v3.0 (Haas et al. 2013) was used to predict candidate coding regions, and then redundant sequences were removed by CDHIT v4.6 (Fu et al. 2012) with identity threshold of 0.98. Raw reads of public data were downloaded (see the supplementary note and table S1, Supplementary Material online) and processed in the same way as the newly sequenced data sets. The raw reads of the species we generated in this study can be accessed on NCBI (https://www.ncbi.nlm.nih.gov/) and NGDC (https://ngdc.cncb.ac.cn/) (see supplementary table S3, Supplementary Material online, for accession numbers). The assembled transcriptomes can be downloaded from Mendeley Data (https://data.mendeley.com/datasets/wp5p3fb9fx/draft?a=e768d2ff-4f9c-4368-9ca5-90d891d86dde; last accessed January 3, 2022).

### Candidate Ortholog Identification and Gene Set Filtering

Orthologous groups were generated from gene sequences of ten representative Poaceae species (see the supplementary note, Supplementary Material online) by OrthoMCL v1.4 (Li et al. 2003). The 1,234 OGs with low-copy nuclear genes were retained as seeds and their sequences were used to search for homologous sequences in all species by HaMStR v13.2 (Ebersberger et al. 2009) with a cutoff e-value of 10^−20^. The resulting nucleotide sequences were aligned with MUSCLE v3.8 (Edgar 2004) with default settings, and poorly aligned regions were trimmed by trimAl v1.4 (Capella-Gutiérrez et al. 2009).

Considering that missing data, short sequences, insufficient informative site, and other factors might result in biased inference, we further selected five subsets of the 1,234 OGs by successively reducing number of genes using the following criteria (see supplementary fig. S3, Supplementary Material online): 1) each OG contains gene(s) from at least one species in each tribes and each of the Poeae subtribes, resulting in 914 OGs; 2) OG with sequences having alignment length ≥450 bp and taxon coverage ≥70%, leading to 763 OGs; 3) OG with sequences having alignment length ≥600 bp and taxon coverage ≥85%, retaining 512 OGs; 4) removal of sequences with misleading signals (such as long-branch attraction and saturation) as suggested by TreSpEx v1.1 (Struck 2014) (supplementary fig. S4, Supplementary Material online), resulting in 373 OGs. The five gene sets (1,234 OGs, 914 OGs, 763 OGs, 512 OGs, and 373 OGs) were all used for phylogenetic analyses. The alignments and trees of each 1,234 OGs can be downloaded from Mendeley Data (https://data.mendeley.com/datasets/wp5p3fb9fx/draft?a=e768d2ff-4f9c-4368-9ca5-90d891d86dde; last accessed January 3, 2022).

### Phylogenetic Analysis

We used the coalescent method to reconstruct the phylogeny of Pooideae. For the low-copy genes, the phylogeny of each of the 1,234 OGs was reconstructed by RAxML v7.2 (Stamatakis 2006) with 100 replicates under GTRGAMMA model. Then, ASTRAL v5.6 (Mirarab et al. 2014) was used to reconstruct Pooideae phylogeny for each of the five OG sets with the 100 replicates from RAxML to obtain the BS values of all nodes. OGs with multiple copies in single species (from the WGD analysis) were further filtered into 802, 480, and 181 OGs (see the supplementary note, Supplementary Material online), which were used to reconstruct species trees by using ASTRAL-Pro (Zhang, Scornavacca, et al. 2020). The phylogenetic reconstruction method for wheat relatives is described in the supplementary note, Supplementary Material online.

### Statistic Tests of Topological Reliability

To investigate the possibility of different topologies of Poeae, we further used ASTRAL-Pro (Zhang, Scornavacca, et al. 2020) to annotate our three-clade Pooideae phylogeny by evaluating its supporting values of LPP (-t 4) and QS (-t 8) for different topologies (the main, the first, and the second alternatives). Besides, the concordance of 1,234 single gene trees onto the different topologies of the Poeae were counted by PhyParts (Smith et al. 2015).

### Dating Divergence Times with Fossil Calibrations

For molecular clock estimates, the input species tree with branch lengths was generated by RAxML v7.2 using the concatenation of 373 OGs from 190 species (supplementary table S1, Supplementary Material online) while fixing the topology as shown in supplementary figure S13, Supplementary Material online. We used a penalized likelihood method in treePL (Smith and O’Meara 2012) to estimate the divergence times based on the ML tree as a phylogenetic reference. Three different calibration strategies were employed (see the supplementary note, Supplementary Material online). We first ran a preliminary analysis with the prime option to determine the best optimization parameters (opt, optad, and optcvad). Then, we performed cross-validation to determine the best smoothing value. Lastly, we performed the formal analysis with the parameters above with the fossil constraints to obtain the time tree of Pooideae. For the sensitivity test of the calibration fossils, we removed each of 15 calibrations one at a time and estimated the age with the same procedure by treePL. The average age of each node from all of the sensitivity tests was compared with above results from the three calibrations (supplementary table S5, Supplementary Material online). For the BEAST analysis (Drummond et al. 2012), considering the computing time, we selected the top 30 (supplementary table S6, Supplementary Material online) of 373 OGs suggested by clock-likeness methods (Smith, Brown, and Walker 2018) as input sequences. The detailed method of the BEAST analysis is described in the supplementary note, Supplementary Material online.

### Ancestral State Reconstruction of Characters

We selected six characters to reconstruct the ancestral states of Pooideae. Information of character states was retrieved from The Grass Genera of the World, Flora of China, and Scientific Database of China Plant Species (for more details, see the supplementary note, Supplementary Material online). The states of characters are listed in supplementary table S7, Supplementary Material online. We used the rayDISC function in the corHMM package (Beaulieu et al. 2013) in R for ancestral character reconstruction based on the ML method. Ancestral states at internal nodes were estimated by marginal probabilities. We performed the analysis with nine different models and root.p arguments, and selected the best-fitting results with the lowest AIC(c) scores and the highest weight (supplementary fig. S22, Supplementary Material online) as suggested by the author of the program.

### Diversification Analysis

BAMM (Bayesian analysis of macroevolutionary mixtures) (Rabosky et al. 2014) and MEDUSA (Modeling Evolutionary Diversification Using Stepwise AIC) (Alfaro et al. 2009) are two widely used programs to detect variation in rates across phylogeny, even though the former has received criticism (Moore et al. 2016). Here, we used the two programs to estimate diversification dynamics based on the time tree in this study with sampling fraction information (supplementary table S8, Supplementary Material online). We calculated the sampling fraction at the tribe level based on species information of Soreng et al. (2017). The detailed description of the implementation BAMM and MEDUSA is described in the supplementary note, Supplementary Material online.

### Gene Tree Mapping, Synonymous Substitution Rate (Ks) Estimation, and Synteny Analysis

To identify GD clusters as possible evidence for WGDs in Pooideae, a total of 164 data sets including eight outgroups were used to assign genes to gene families essentially following previous methods (Yang et al. 2015, 2018) (see the supplementary note, Supplementary Material online, for details). Finally, 45,722 of aligned homolog group were used to reconstruct a final ML gene tree by RAxML v7.2 (Stamatakis 2006) with GTRCAT model. We performed tree reconciliation analysis by comparing gene trees with a reference species tree (supplementary fig. S23, Supplementary Material online) as described in previous studies (Huang, Zhang, et al. 2016; Xiang et al. 2017; Ren et al. 2018; Leebens-Mack et al. 2019). The GD was counted at each of the MRCA node by Tree2GD (https://github.com/Dee-chen/Tree2gd; last accessed July 25, 2021) with ≥50% BS of the divergence of two subclades. The synonymous substitution per site (Ks) for paralogs in a specific taxon was calculated as described previously (Leebens-Mack et al. 2019; Zhang, Zhang, et al. 2020) (see the supplementary note, Supplementary Material online). Moreover, gene collinearity in assembled genome was analyzed by MCScanX (Wang et al. 2012), then the intersection between syntenic gene pairs and duplicates derived from the phylogenetic method of GD cluster is used as evidence of genome doubling.

### Annotation of Expanded Gene Families and GO Enrichment Analysis

Among genes that experienced GD during Pooideae evolution, we selected the genes (gene families when considering homologs from multiple species) with species coverage ≥20 species and ≥5 species in both of the duplicated subclades, to represent genes that have expanded in a wide range of species. Then, the gene sequence(s) from any of the three Pooideae genomes (*T. aestivum*, *Hordeum vulgare*, or *B. distachyon*) were used to identify homologs in *Oryza sativa* or *Arabidopsis thailiana*, with their annotated functions listed in supplementary table S11, Supplementary Material online. We further carried out GO enrichment analysis using AnnotationHub (Morgan 2019) and clusterProfiler (Yu et al. 2012) packages of R to find the enriched functional categories for duplicates of all candidate WGDs and CGD5.

### Molecular Evolution of *AP1/FUL* and *CBF* Homologs

To reconstruct the evolutionary history of *AP1*/*FUL* homologs, we selected a total of 46 species (supplementary table S13, Supplementary Material online). We retrieved candidate *AP1*/*FUL* homologs of all representatives by using HMMER v3.1 (Mistry et al. 2013) with the entry domain (PF01486.17) and default settings. To identify *CBF* homologs, with are members of a subclade of the AP2/ERF superfamily containing the AP2 domain, we performed a BLASTP search (e-value cut off = 10^−5^) to favor genes that are more closely related to *CBF*. The treatments of the retrieved protein sequences are described in the supplementary note, Supplementary Material online. In addition, we used MCScanX (Wang et al. 2012) and location of gene on the chromosome to confirm the type of duplication.

### MuSSE Analysis for GD-Related Diversification Rates

To investigate potential association between the GDs (CGD and WGD) and diversification rate, we performed MuSSE analysis (Multiple State Speciation and Extinction) (Maddison et al. 2007) to investigate the effect of times of duplication on diversification rate in the resolved phylogeny (FitzJohn et al. 2009). The R package of diversitree v0.9.11 (FitzJohn 2012) was used for the analysis. The numbers of times each species has experienced GDs detected in this study are used as traits. Transition of character states were constrained to change stepwise as 1<>2<>3<>4–8. We selected the best-fitting model from four different models (i.e., free lambda and mu, fixed lambda, fixed mu, and fixed lambda and mu for each state) by the lowest AIC value and the highest AIC weight (supplementary table S12, Supplementary Material online). Then we ran the MCMC chain for 5,000 generations and sampled every 100 generations with an exponential prior as 1/(2r), where r was the lambda of the species with the original state.

### Correlations between Diversification Rate Upshifts, Trait Transitions, and GDs (CGD and WGD)

A statistical test for the correlation of diversification rate shifts, trait transition, and GDs (CGD and WGD) was performed following the method of Tank et al. (2015) (also see Landis et al. [2018]) as the coincidence between two of the three features (diversification rate shifts vs. trait transition, trait transition vs. GDs, and GDs vs. diversification rate shifts). For example, for association between GDs and diversification rate shifts, the number of coincidence of observed data (e.g., GDs and diversification rate upshifts observed in this study) were compared with two alternative null hypotheses: 1) coincidences between the observed upshifts and randomly distributed GDs (r.wgd); 2) coincidences between the observed GDs and randomly distributed upshifts of diversification rates (r.shift). Each test with 1,000 random distributions was repeated 100 times to produce the result of the distribution of *P* values.

## Supplementary Material

Supplementary data are available at *Molecular Biology and Evolution* online.

## Supplementary Material

msac026_Supplementary_Data
