# Supplementary material for: Phylotranscriptomics Resolves the Phylogeny of Pooideae and Uncovers Factors for Their Adaptive Evolution
Source: Mol Biol Evol. 2022 Feb 3;39(2):msac026. doi: 10.1093/molbev/msac026 (PMC8844509; doi:10.1093/molbev/msac026)
Supplement: msac026_Supplementary_Data [file msac026_supplementary_data.zip › Supplementary_note.docx]

**Phylotranscriptomics Resolves the Phylogeny of Pooideae and Uncovers Factors for Their Adaptive Evolution**

Lin Zhang^1^, Xinxin Zhu^2^, Yiyong Zhao^1^, Jing Guo^1^, Taikui Zhang^1^, Weichen Huang^3^, Jie Huang^1^, Yi Hu^3^, Chien-Hsun Huang^1*^, Hong Ma^3*^

^1^State Key Laboratory of Genetic Engineering and Ministry of Education Key Laboratory of Biodiversity Sciences and Ecological Engineering, Institute of Plant Biology, Institute of Biodiversity Sciences, School of Life Sciences, Fudan University, Shanghai 200433, China

^2^College of Life Sciences, Xinyang Normal University, Xinyang, 464000, China

^3^Department of Biology, the Huck Institutes of Life Sciences, the Pennsylvania State University, University Park, PA, USA

*Corresponding authors: Hong Ma (hxm16@psu.edu); Department of Biology, Huck Institutes of the Life Sciences, Pennsylvania State University, University Park, PA, 16802 USA. Chien-Hsun Huang, E-mail: huang_ch@fudan.edu.cn; School of Life Sciences, Fudan University, 2005 Songhu Road, Shanghai 200433, China.

**Table of Contents**

[**1. Relationships of Poeae Subtribes and Wheat Relatives** 2](#_Toc90803649)

[1.1 Relationships Among Poeae Subtribes 2](#_Toc90803650)

[1.2 Relationships Among Wheat Relatives (the *Triticum*-*Aegilops* Complex) 5](#_Toc90803651)

[**2. Molecular Dating Analyses with Different Calibration Sites and Methods** 6](#_Toc90803652)

[**3. A Comparison of Diversification Rate Shifts Proposed From Previous Reports and This Study** 8](#_Toc90803653)

[**4. Materials and Methods (Details)** 9](#_Toc90803654)

[4.1 Taxon Sampling, Sequencing, and Sequence Assembly 9](#_Toc90803655)

[4.2 Candidate Ortholog Identification and Gene Set Filtering 9](#_Toc90803656)

[4.3 Phylogenetic Analysis 10](#_Toc90803657)

[4.4 Molecular Dating of Divergence Times with Fossil Calibrations 11](#_Toc90803658)

[4.5 Ancestral State Reconstruction of Characters 12](#_Toc90803659)

[4.6 Diversification Analysis 13](#_Toc90803660)

[4.7 Gene Tree Mapping, Synonymous Substitution Rate (Ks) Estimation and Synteny Analysis 13](#_Toc90803661)

[4.8 Molecular Evolution of *AP1*/*FUL* and *CBF* Homologs 15](#_Toc90803662)

[**5. References** 16](#_Toc90803663)

**1. Relationships of Poeae Subtribes and Wheat Relatives**

## 1.1 Relationships Among Poeae Subtribes

Soreng et al. (2017) classified the largest Pooideae tribe Poeae into 26 subtribes, and here we resolve the relationships of all sampled 24 subtribes with moderate to strong support (80%-100% BS) for over 80% of nodes (fig. 2 and supplementary figs. S5-S9). Our nuclear phylogeny is different from the previous topology of two groups (PCG1 and PCG2) of Poeae subtribes from analyses using plastid markers (Soreng and Davis 1998; Soreng et al. 2017) (supplementary fig. S2), with two new clades, named here as Poeae nuclear group 1 (PNG1) and Poeae nuclear group 2 (PNG2) (fig. 2 and supplementary fig. S2d), with several differences in relationships among subtribes (supplementary fig. S2a-c). In our result, PNG1 include two clades: Clade I and Clade II. Clade I includes all eight subtribes of PCG1 plus Holcinae and Sesleriinae of PCG2, whereas five subtribes of PCG2 formed Clade II as sister to Clade I with 100% BS; the remaining nine subtribes of PCG2 for another clade, which is named PNG2 here and is sister to all other Poeae subtribes (fig. 2, supplementary figs. S2d and S5-S9). Relationships of subtribes in Clade I and Clade II are consistent in all five trees, and a highly supported topology is shown for PNG2, with alternative placement of Cinninae and *A. pubescens* in some trees (see below).

*Relationships Among PNG1*

Within PNG1, Clade I contains ten subtribes (eight of PCG1 and two from PCG2) with maximal support in all five trees. The first divergent lineage is Phalaridinae + Torreyochloinae with strong support (four trees ≥ 80% BS and two of them ≥ 90% BS); next Holcinae is sister to a clade with the other seven subtribes (four trees ≥ 80% BS and two of them with BS ≥ 90%). Then, a clade of Aveninae + Sesleriinae (four trees with BS ≥ 90%) is sister to a clade with the other five subtribes (≥ 95% BS). For the remainder (five subtribes) of Clade I, Anthoxanthinae is sister to the well-supported supersubtribe Agrostidodinae (four trees ≥ 80% BS and two of them with BS ≥ 90%), which was defined by Soreng et al. (2017) and received ≥ 90% BS in four trees here, with divergences of subtribes in the order of Brizinae, Echinopogoninae, Calothecinae and Agrostidinae with ≥ 90% BS in at least four trees.

In Clade II of PNG1, the grouping of the five subtribes is consistent in all five trees albeit with moderate support (one tree with 81% BS). In our result, Airinae is the first divergent lineage and sister to the supersubtribe Loliodinae with four subtribes. In Loliodinae, Loliinae and Dactylidinae are successive sisters to Parapholiinae + Cynosurinae, with maximal support in all five trees.

*Relationships Among PNG2*

PNG2 contains nine subtribes of PCG2 and *A. pubescens* (*incertae sedis*) with maximal support in all trees. Aristaveninae is sister to the PPAM clade (fig. 2) (Soreng et al. 2015) comprised of the remaining eight subtribes (100% BS), with Miliinae + Coleanthinae (100% BS) being the first to separate from others of the PPAM clade (100% BS). In addition, the remaining six subtribes form two clades, one including Poinae and Phleinae and the other containing the subtribes Cinninae, Beckmanniinae, Alopecurinae and Ventenatinae, with inconsistent placement for *A. pubescens* (fig. 2 and supplementary fig. S2d). *A. pubescens* was considered either related to Poinae or as an intergeneric hybrid (Soreng and Davis 2000). Recent plastid phylogenies placed it in PCG2 (Soreng et al. 2015) and further in the PPAM clade (Soreng et al. 2017), but without a more specific position. Here we report two alternative positions for *A. pubescens* (supplementary fig. S10): (i) sister to a clade of four subtribes (Cinninae, Beckmanniinae, Alopecurinae and Ventenatinae) in two trees with strong support (93% BS from 512 OGs and 84% BS from 373 OGs; supplementary figs. S8-S9); (ii) sister to Phleinae in three trees of using more OGs (1234 OGs, 914 OGs, and 763 OGs) but with weak support (< 60% BS; supplementary figs. S5-S7). Considering the former topology has a higher BS inferred from two gene sets (512 and 373 OGs), after the removal of biased signals, we used the topology (i) for further analyses. Among the four subtribes that are sister to *A. pubescens*, there is stronger support for Cinninae and Beckmanniinae being successive sisters to Alopecurinae + Ventenatinae.

We also used multi-copy genes to reconstruct the phylogeny by ASTRAL-pro (Zhang et al. 2020a). The results (supplementary figs. S45-S47) are almost the same as the topology using single-copy genes (figs 1 and 2), except for the position of five subtribes of Poeae (Airinae, Echinopogoninae, Anthoxanthinae, Holcinae and Beckmanniinae) and Diarrheneae II (supplementary figs. S44). In the multi-copy trees, Echinopogoninae is placed within the subtribe Aveninae (T2) while Anthoxanthinae is sister to the MRCA of Agrostidnae and Sesleriinae (T4). Holcinae is sister to the MRCA of Agrostidnae and Torreyochloinae (T6), and Beckmannlinae is sister to the MRCA of Alopecurinae and Cinninae (T8). Airinae is no longer within the Clade II but is sister to the Clade I and Loiodinae (T10), whereas Diarrheneae II is nested with the Stipeae (T12). Generally, bootstrap values of at least one node associated with the alternative placement in the multi-copy topologies are lower than the single-copy topologies (supplementary figs. S44b). These differences might be a result of more complex evolution of multi-copy genes, including the possibility of lineage-specific duplications and losses.

To further compare the previous Poeae topology (T1) with PCG1 and PCG2 using plastid sequences with the topology (T2) proposed in this study with PNG1 and PNG2, single-gene trees of the 1234 OGs were compared with the T1 and T2 topologies by using Phypart (supplementary fig. S33) (Smith et al. 2015). When a node of a gene tree includes the same group of species at a node on the compared topology, a concordance is counted on the node of the compared topology. We found that the numbers of the concordances for the MRCAs of PCG1 and PGC2 (T1), respectively, are 15 and 4, whereas the numbers for the MRCAs of PNG1 and PNC2 (T2), respectively, are 113 and 202. Furthermore, we used the function of “newick annotations” in ASTRAL-pro (Zhang et al. 2020a) to evaluate support for each branch in the topology with both quartet support (QS) and local posterior probability (LPP). This analysis provides an estimate of the amount of gene tree conflict concerning the branch. The QS represents the percentage of quartets in our gene trees that agree with branch, whereas the LPP is computed based on a transformation of the QS. If a branch in species tree is consistent with the quartet analysis, the probability of observing the species tree is expected to be at least 1/3; the higher the score, the more single gene trees supporting this topology. In addition, when evaluating the topology for each node in a given species tree, ASTRAL-pro also evaluate the other two alternative topologies at the same time. For example, if the topology of a node in the species tree is ((A,B),C), the other two alternatives are ((B,C),A) and ((A,C),B). We evaluated our species tree for the topology regarding Clade I and Clade II of PNG1 and PNG2 [T2 = ((Clade I, Clade II), PNG2)] with QS and LPP values calculated from the single gene trees of each of the five sets of OGs (supplementary figs. S34-S43). The values are 0.51/0.5/0.51/0.53/0.53 of QS and 1/1/0.53/1/1 of LPP for the five OG sets, respectively. Moreover, the values of the other two alternative topologies are 0.31/0.31/0.31/0.3/0.3 [for (Clade I, (Clade II, PNG2))] and 0.18/0.18/0.18/0.17/0.17 [for ((Clade I, PNG2), Clade II)] from QS and 0/0/0.3/0/0 and 0/0/0.1/0/0 of LPP in five OG sets, respectively. The much higher values of T2 (more than half of QS and maximum of LPP) than the other two alternatives indicate the reliability of the three-clade topology of the Poeae.

## 1.2 Relationships Among Wheat Relatives (the *Triticum*-*Aegilops* Complex)

Wheat was domesticated in the Fertile Crescent area, have shaped the early human civilization and contains three subgenomes (A, B and D) (Salamini et al. 2002; Marcussen et al. 2014). It was thought to have involved natural hybridization among species in *Triticum* and *Aegilops*, resulting in an intricate relationship of the *Triticum*-*Aegilops* complex, in the tribe Triticeae (Marcussen et al. 2014; Li, Liu, et al. 2015; Glémin et al. 2019). Marcussen et al. (2014) proposed that the D lineage (wheat subgenome) might have arisen through a homoploid hybrid between the A and B lineages. However, Glémin et al. (2019) argued that the origin of the D lineage was likely more complex than such a single hybridization event. To examine the relationships of the *Triticum*-*Aegilops* complex, including the three wheat subgenomes, in a bipartition fashion, further phylogenetic analyses were performed with 18 representatives from Triticeae (supplementary table S1) using the aforementioned 1234 OGs. As the taxon sampling here is much reduced compared with that of the subfamily, we performed a second set filters of genes by length and missing data according to alignment, as well as the mean BS of gene tree (supplementary fig. S11) to obtain a new series of five OGs (369 OGs, 261 OGs, 158 OGs, 124 OGs and 81 OGs) to reconstruct the phylogeny of the *Triticum*-*Aegilops* complex, with the subgenomes separately represented (supplementary fig. S12). In addition to the hexaploid bread wheat, our sampling also included two tetraploids, *T. dicoccoides* (AABB) (Avni et al. 2017) and *Aegilops geniculata* (UUMM) (Li, Liu, et al. 2015).

The summarized phylogeny (fig. 1b) groups the A subgenomes of three *Triticum* species in one clade, and the B subgenomes of two *Triticum* species in another clade. Moreover, the M (*A*. *geniculata*) and S (*A*. *sharonensis*) subgenomes are successive sisters of the D subgenomes (*T. aestivum* and *A. tauschii*), whereas the U subgenome (*A. geniculata*) is sister to the A subgenomes. Our result (fig. 1) supports the hypothesis that A and D have a slightly closer relationship than B, in agreement with the topology of Glémin et al. (2019). Meanwhile, our results are in good agreement with Marcussen et al. (2014) and Glémin et al. (2019). When the gene trees of the 369 OGs were filtered with ≥ 60% BS and then examined for relationships among the A, B, D subgenomes, we found the numbers of gene trees with the three topologies ①((A, D), B)), ②((B, D), A) and ③((A, B), D) to be, respectively, 75, 52, and 33. The first two topologies together support the hypothesis that D originated from a hybridization between A and B as proposed by Marcussen et al. (2014), but the third one suggests that the D might have experienced more complex events as proposed by Glémin et al. (2019). As for the allotetraploid *A. geniculata*, it was reported to be widely-distributed in the Mediterranean (Arrigo et al. 2010) and showed a close relationship to S and D genomes from chloroplast markers (Li et al. 2015a). Our nuclear result shows that one subgenome of *A. geniculata* (UU) is close to the *Tricicum* A subgenomes, and the other (MM) is close to the D and S subgenomes, providing new evidence for frequent gene flow among members of the *Aegilops*-*Triticum* complex.

In conclusion, we present a robust nuclear phylogeny for Pooideae and provide a clear picture of relationships among tribes and subtribes of Pooideae and further phylogenetic understanding of wheat relatives. By our results, we find several cytonuclear discordances between plastid and nuclear phylogenies, possibly due to the nature of genetic inheritance, reticulation, hybridization and other events (see Discussion in the main text).

# 2. Molecular Dating Analyses with Different Calibration Sites and Methods

To further test the reliability of our molecular clock estimations, we removed the calibrations at a times, exception the secondary calibration N1 (assigned to the stem group of monocots) and inferred the estimation by treePL. The results (supplementary table S5) indicate that thirteen of the tests yielded similar results to those of treePL analyses using calibrations 1 to 3, except the test after the removal of N10 (assigned to crown group of Poaceae) that has a greater influence on the age. This result is similar with previous dating results: the use of the N10 (Schubert et al. 2019) are all earlier than those not used (Christin et al. 2014). However, when we averaged the age of each node in all tests, the age differences between our dating result and sensitivity tests at each MRCA node of major clades were less than 4 My (supplementary table S5), indicating the general reliability of our estimation.

In addition, the top 30 (supplementary table S6) of the 393 OGs suggested by the clock-likeness method of Smith et al. (2018) were used for the BEAST dating analysis (Drummond et al. 2012) with Calibration 1. The dating results of BEAST analysis (supplementary fig. S15 and supplementary table S5) are generally older than those using treePL at the subfamily level (such as stem group of Pooideae in 85.6 Mya, CI: 81.5-89.6, as compared to ~68.8 Mya with CI of 0.8 My from treePL), but are similar for many lineages within Pooideae from treePL (such as the age at the MRCA of core Pooideae at 37.9 Mya, CI: 34.7-41.3 from BEAST, vs 35.7 Mya, CI:35.5-35.9 from treePL). This could be partly due to the difference in estimation method (MCMC chains with Bayesian frameworks v.s. penalized likelihood with maximum likelihood frameworks). Because of the large supermatrix with hundreds of genes or tips is hard for the MCMC chains of BEAST analysis to converge with stability of the result within a reasonable amount of time; thus we selected the top 30 OGs as indicated by a clock-likeness method for the BEAST analysis. The smaller number of OGs that were used in the analysis probably also contributed to the greater CI ranges of the dating results from BEAST, although the older origins of the early-divergent Pooideae lineages also suggest the uncertainties in dating results (see next paragraph for more details of results from the BEAST analysis and comparison with treePL results).

The BEAST results (supplementary fig. S15 and supplementary table S5) estimated the age of the Pooideae stem lineage as ~85.6 Mya (CI: 81.5-89.6); four early divergent Pooideae clades had originated in the late Cretaceous, Brachyelytreae (79.3 Mya, CI: 75.4-82.6), Nardodae (72.8 Mya, CI: 70.0-76.7), Duthieeae (70.4 Mya, CI: 67.9-73.3) and the ancestor of the remaining Pooideae (68.7 Mya, CI: 65.0-71.7). Correspondingly, the treePL results estimated a late Cretaceous origin of Pooideae at ~68.8 Mya, CI: 68.3-69.1, and also subsequent divergences of Brachyelytreae (59.0 Mya, CI: 58.6-59.4), Nardodae (54.7 Mya, CI: 53.8-54.5), and the ancestor of the remaining Pooideae (45.5 Mya, CI: 45.2-45.8). The origins of four other major Pooideae clades were estimated by BEAST in the Paleocene: Phaenospermateae and Melicodae at 64.5 Mya (CI: 53.1-71.1) as compared to ~44.5 Mya with CI of 0.4 My from treePL, Diarrheneae II + Stipodae and Diarrheneae I + core Pooideae at 59.2 Mya from BEAST (CI: 65.0-71.7) vs 45.5 Mya, CI: 45.2-45.8 from treePL. In the Eocene, BEAST estimated origins for seven major clades: namely five tribes (such as Diarrheneae I, 48.2 Mya, CI: 40.7-56.3, Stipeae, 37.1 Mya, CI: 34.3-45.7, Brachypodieae, 43.4 Mya, CI: 39.3-47.1), one supertribe (Triticodae, 37.9 Mya, CI: 34.7-41.3) and Poeae 37.9 Mya, CI: 34.7-41.3, meanwhile, the results of treePL are generally similar to those from BEAST at these nodes with differences < 4 My except for Diarrheneae I with a difference of 10.6 My. Since the Oligocene according to BEAST, several subtribes/clades of Poeae were found to have split, including Airinae (29.2 Mya, CI: 25.7-32.0), Holcinae (26.1 Mya, CI: 23.7-28.4) and MRCA of Phalaridinae+Torreyochloinae (21.7, CI:15.4-25.7), also most stem group of subtribes and some genera have diverged from each other, such as Dactylidinae (20.6 Mya, CI: 17.7-23.4), Loliinae (24.1 Mya, CI: 17.5-21.8), Festuca (16.6 Mya, CI: 13.7-18.9), Poinae + Phleum (14.7 Mya, CI: 12.6-17.0), and Lolium (9.2 Mya, CI: 3.0-9.3). Again, the results of treePL are generally close to those from BEAST with differences < 5 My. In addition, the larger CI ranges of some nodes from BEAST raised possible alternative hypothesis than links to cooling paleoclimates; for example, the crown age of Duthieeae was estimated ~ 51.3 Mya with CI (34.2 to 69.1) from the late Cretaceous to E-O transition, while the global climate was still quite warm from ~69 to 48 Mya, before the global cooling period from the early-middle to late Eocene (~48-33 Mya). Similarly, the age (with the CI) of the MRCA of Diarrheneae II ＋ Stipodae (48.2 Mya, CI: 40.7-56.3) could be older than the aforementioned cooling period. In addition, the ages with their CI ranges of the MRCA of, respectively, Stipeae (31.9 Mya, CI: 23.7-41.9) and Melicodae (28.4 Mya, CI: 21.3-38.4) partially overlap with, yet could be younger than, the same cooling period. The generally older ages in the early-divergent Pooideae tribes or subclades and larger CI ranges from the BEAST analysis suggest uncertainties in molecular dating results and the need to be cautious in linking the divergences of these Pooideae lineages to specific geological times.

# 3. A Comparison of Diversification Rate Shifts Proposed From Previous Reports and This Study

The diversification rate of the Pooideae was also estimated in Spriggs et al. (2014) and Pimentel et al. (2017), which used different taxon ranks in their analyses. Specifically, Pimentel et al. (2014) and our study were conducted above the genus level, while the Spriggs et al. (2014) study was conducted at the genus level. Moreover, the phylogenies of Pooideae used as reference are also different among this study and those by Spriggs et al. (2014) and Pimentel et al. (2017); in particular, the relationships of early-divergent tribes (Phaenospermateae and Ampelodesmeae) and subtribes within Poeae were resolved in this study (figs. 1-2 and supplementary figs. S1-S2), but not in the previous studies. Comparing the results, some shifts of diversification rate are consistent, such as the shift close to the core Pooideae (Spriggs et al. 2014; Pimentel et al. 2017), and the shifts near nodes of early-divergent lineages (Stipeae + Meliceae in Pimentel et al. (2017)). However, several more recent shifts such as those associated with *Poa*, *Festuca* and *Stipa* proposed by Spriggs et al. (2014) were not detected in our analysis. These differences might be partly explained by the difference of sampling and Pooideae phylogeny among this and previous studies.

Our result also indicates that the accelerations in diversification rate are associated with the species richness of large tribes (Poeae, Stipeae and Meliceae) or other large clades (the core Pooideae, Triticeae + Bromeae, and the clade with Phaenospermateae and the core Pooideae), suggesting that upshift of diversification rate could have progressively increased the diversity of Pooideae at different times of evolutionary history. Similar conclusions have also been mentioned in previous studies ([Spriggs et al. 2014](#_ENREF_123); [Pimentel et al. 2017](#_ENREF_93)). Our result further reveals that subgroups of large and highly diversified group have dramatically different degrees of species richness (such as between Poeae/Triticeae and Littledaleeae among tribes of the core Pooideae), leading to the highly uneven distribution of biodiversity among Pooideae groups.

# 4. Materials and Methods (Details)

## 4.1 Taxon Sampling, Sequencing, and Sequence Assembly

(For ease of understanding the context, some of the information here is redundant with the main text)

To reconstruct a phylogenetic tree for Pooideae, we strived to sample taxa represent all 15 tribes and nearly all subtribes (24 of 26) in Poeae, the largest Pooideae tribe. In particular, the sampled taxa represent 13 and 14 genera, respectively, of two other large tribes, Stipeae (28 genera) and Triticeae (27 genera). Overall, a total of 157 species were sampled in Pooideae as ingroups, and 38 other species as outgroups across monocots (Poales, Zingiberales, Arecales, and Asparagales) and eudicots (Ranunculales, Brassicales and Vitales) (supplementary table S1). Among the sampled species, 161 are newly sequenced (supplementary table S3). For transcriptome sequencing, total RNA was extracted from fresh leaves (sometimes with inflorescence, often without flash freezing using liquid nitrogen) or seeds (soaked with water) by using the ZR Plant RNA MiniPrep kit (Zymo Research, Orange, CA). Paired-end RNA-seq was performed to generate reads of 2×150 bp using the Illumina technology with HiSeq3000. Raw reads were assembled into contigs using Trinity v2.9 (Grabherr et al. 2011) with default settings. TransDecoder v3.0 (Haas et al. 2013) was used to predict candidate coding regions, and then redundant sequences were removed by CDHIT v4.6 (Fu et al. 2012) with DNA sequence identity threshold of 98%. Public data were retrieved from several databases (supplementary table S1), including NCBI (https://www.ncbi.nlm.nih.gov), Ensembl (https://asia.ensembl.org/index.html), CoGe (https://genomevolution.org/coge/), Phytozome (http://phytozome.jgi.doe.gov/pz/portal.html), and other sources, including the OneKP Project (https://sites.google.com/a/ualberta.ca/onekp/home) (Leebens-Mack et al. 2019), Zeng et al. (2014), Peng et al. (2013) and datasets generated in our lab. Raw reads of public data were processed in the same way as the newly sequenced datasets.

## 4.2 Candidate Ortholog Identification and Gene Set Filtering

To generate “seeds” of candidate low-copy genes as query sequences, we selected ten species across Poaceae, including five in Pooideae to represent this largest subfamily (*Brachypodium distachyon*^g^*, Hordeum vulgare*^g^*, Stipa aliena*^t^*, Phaenosperma globosum*^t^, *Lygeum spartum*^t^; ^g^ or ^t^ denote genomes or transcriptomes, respectively); five species were chosen to represent other large subfamilies, *Phyllostachys heterocycla*^g^ (Bambusoideae)*, Oryza sativa*^g^ (Oryzoideae)*, Sorghum bicolor*^g^ and *Setaria italica*^g^ (Panicoideae)*, Eleusine coracana*^t^ (Chloridoideae). In addition to representing Pooideae and other large subfamilies, diploid genomic datasets were favored if they have relatively high quality, but the *Eleusine coracana* transcriptome was used because the only available Chloridoideae genome (*Eragrostis tef*) is an allotetraploid with a relatively low N50 of 85 Kb and a large number of scaffolds (14,000 for those ≥ 1,000 bp) (Cannarozzi et al. 2014). Orthologous groups (OGs) were generated from gene sequences of ten Poaceae representatives by OrthoMCL v1.4 (Li et al. 2003); among the OGs, each of the 1234 OGs with a total 10-12 genes and at least one gene from each of the ten species were retained as seeds of likely low-copy nuclear genes. We used the sequences of the 1234 seeds as queries to search for homologous sequences in all species using HaMStR v13.2 (Ebersberger et al. 2009) with a cutoff e-value of 10^-20^. The resulting nucleotide sequences were aligned with MUSCLE v3.8 (Edgar 2004) with default settings, and poorly aligned regions were trimmed using trimAl v1.4 (Capella-Gutiérrez et al. 2009).

Considering that missing data, short sequences, insufficient number of informative sites and other factors might result in biased inference, we further selected five subsets of the 1234 OGs by successively reducing number of genes using increasing number of the following criteria (also see supplementary fig. S3): (1) each OG contains gene(s) from at least one species in each tribes and each of the Poeae subtribes, resulting in 914 OGs; (2) those of the 914 OG with sequences having alignment length ≥ 450 base pairs (bp) and taxon coverage ≥ 70%, leading to 763 OGs; (3) those of the 763 OG with sequences having alignment length ≥ 600 bp and taxon coverage ≥ 85%, retaining 512 OGs; (4) among the 512 OGs, with removal of sequences with misleading signals (such as long-branch attraction and saturation, supplementary fig. S4) as suggested by TreSpEx v1.1 (Struck 2014), resulting in 373 OGs. The five gene sets (1234 OGs, 914 OGs, 763 OGs, 512 OGs and 373 OGs) were all used for phylogenetic analyses.

## 4.3 Phylogenetic Analysis

We used the coalescent method to reconstruct the phylogeny of Pooideae. Using the low-copy genes, the phylogeny of each of the 1234 OGs was reconstructed by RAxML v7.2 (Stamatakis 2006) with 100 replicates under the GTRGAMMA model. Then, ASTRAL v5.6 (Mirarab et al. 2014) was used to reconstruct the Pooideae phylogeny for each of the five OG sets and the bootstrap values for the coalescent trees were obtained using the combined results from the replicates for all gene trees (100 replicates for each gene tree) in the corresponding OG set (with the setting of -b parameter). Furthermore, multi-copy OGs were selected from gene families (45722 OGs) used in the WGD section (see below) using the following criteria. First, the number of sequences in each OG does not exceed 480 genes, resulting in 20004 OGs. Next, at least two gene copies are found in each of at least 70% of Pooideae species, with 802 OGs meeting this condition. If over 75% of species have two copies of each gene, 480 OGs remained. Then, when over 80% of species had two gene copies in each OG, there were 181 OGs. All OG sets were used to reconstruct the Pooideae phylogeny by ASTRAL-Pro (Zhang et al. 2020a).

In addition, to reconstruct the phylogenetic tree of wheat and its close relatives, we analyzed genes of each subgenomes of the hexaploidy wheat (A, B and D) and wild emmer wheat (A and D) separately. The allotetraploid *A. geniculate* lacks subgenome information, thus we identified genes belonging to putative subgenomes according to their phylogenetic positions on gene trees. These subgenomic datasets and diploid wheat relatives totaled 18 taxa. We used the same initial set of the 1234 OGs to select five new subsets (supplementary fig. S11), for phylogenetic reconstruction, using the same method as Pooideae phylogeny reconstruction.

## 4.4 Molecular Dating of Divergence Times with Fossil Calibrations

We used a penalized likelihood method to estimate the divergence times of Pooideae lineages by treePL (Smith and O’Meara 2012) based on the ML tree with 190 species (supplementary table S1). The input ML tree with branch lengths was generated by RAxML v7.2 using the concatenation of 373 OGs while fixing the topology as in supplementary fig. S13. As calibration points likely affect the result of molecular dating (Sauquet et al. 2011; Christin et al. 2014), we selected 15 fossils (set as minimum age constraints) and one secondary calibration (set as minimum and maximum age constraint) (supplementary table 4); these fossils have been widely used and were reevaluated recently (Iles et al. 2015; Magallón et al. 2015). The phytoliths found in dinosaur dung from the Late Cretaceous (Prasad et al. 2011) was controversial in calibration placement from previous studies. Thus, our dating analyses used three calibration strategies with exclusion (Calibration 1) or inclusion (Calibration 2 and 3) of this fossil (N13) at two different sites: either Oryzeae (Calibration 2) or Oryzoideae (Calibration 3), as suggested by a previous study (Prasad et al. 2011). We first ran a preliminary analysis with the prime option to determine the best optimization parameters (opt, optad, and optcvad). Then, we performed cross-validation to determine the best smoothing value. Lastly, we performed the formal analysis with the parameters above with the fossil constraints to obtain the time tree of Pooideae.

For the sensitivity test of the calibration fossils, we removed the calibrations at a time except the secondary calibration N1 and estimated the age with the same procedure by treePL. The average age of each node was compared with above results (supplementary table S5). For the BEAST analysis, considering the computing time, we selected the top 30 (supplementary table S6) of 373 OGs suggested by clock-likeness methods (Smith et al. 2018) as input sequences. We used the GTR+G model for site substitution, the birth-death (BD) model for the tree prior and uncorrelated relaxed lognormal model for the clock prior. For all calibrations, we used lognormal prior distribution with the mean of 0 and standard deviation of 0.5. The offset value (age) for each calibration is listed in supplementary table S4, whereas for the secondary calibration at root, the offset (age) was set as 131.6 to provide a central 95% probability range covering 132-134 Mya. We fixed the tree topology using our summary phylogeny (figs. 1-2), and performed two independent runs with 30,000,000 generations. The effective sample size (ESS) was evaluated using Tracer v.1.7 (Rambaut et al. 2018) and independent runs with the same settings (calibration sets) were combined using LogCombiner (Drummond et al. 2012; Bouckaert et al. 2014). The chronogram with mean ages and 95% highest posterior distribution (HPD) of each node was generated using TreeAnnotator (Bouckaert et al. 2014) with the first 20 percent trees discarded as burnin.

## 4.5 Ancestral State Reconstruction of Characters

We selected six characters to reconstruct the ancestral states of Pooideae. Information of character states was retrieved from The Grass Genera of the World (https://www.delta-intkey.com/grass/index.htm#), Flora of China (http://www.efloras.org/flora_page.aspx?flora_id=2), and Scientific Database of China Plant Species (http://db.kib.ac.cn/). The states of characters are listed in supplementary table S7. As the number of species in the phylogeny here is not sufficient for a proper ancestral character analysis at the species level, we have collapsed the tips to the genus level and used coding for either a single or multiple state(s) as appropriate for individual genus; the genera sampled here covered 40% of Pooideae genera, which represented 82% of Pooideae species. We adopted ML method in the corHMM R package (Beaulieu et al. 2013) to reconstruct the ancestral state of characters. Ancestral states at internal nodes were estimated by marginal probabilities. We performed analyses using nine different models and root.p arguments, and selected the best-fitting results with the lowest AIC(c) scores and the highest weight (supplementary fig. S22) as suggested by the author of the program.

## 4.6 Diversification Analysis

BAMM (Bayesian analysis of macroevolutionary mixtures) (Rabosky et al. 2014) and MEDUSA (Modeling Evolutionary Diversification Using Stepwise AIC) (Alfaro et al. 2009) are two widely-used programs to detect variation in diversification rates across a phylogeny, although the former has received some criticism (Moore et al. 2016). Here, we used the two programs to estimate diversification dynamics based on the time tree in this study with the sampling fraction information (supplementary table S8). Considering incomplete taxon sampling at the species level, we calculated the sampling fraction at the subtribe level based on species information of Soreng et al. (2017). In BAMM, we used setBAMMpriors function in BAMMtools package in R to obtain parameters of lambdaInitPrior, lambdaShiftPrior and muInitPrior. We performed analyses using time-constant or time-variable models and conducted two independent runs of 5,000,000 generations with each model with sampling every 1,000 generations. We used the package CODA of R to monitor MCMC convergence. The first 25% was discarded as burn-in. We analyzed results from BAMM using the package BAMMtools (Rabosky et al. 2014) of R. In MEDUSA, birth-death (BD) model with AICc (corrected AIC) criterion and mixed model (allowing the auto-detection to use BD or Yule model) with AICc were fitted to phylogeny.

## 4.7 Gene Tree Mapping, Synonymous Substitution Rate (Ks) Estimation and Synteny Analysis

To identify gene duplication clusters as possible evidence for WGDs in Pooideae, a total of 164 datasets including eight outgroups were used to assign genes to gene families essentially following previous methods (Yang et al. 2015; Yang et al. 2018). In detail, gene families across all species were identified through the all-by-all BLASTN with an e-value cut-off of 10^-5^. The sequence hits were retained when their identities to others were ≥ 50% and their lengths were ≥ 70% of the alignment; the remaining sequences were then further analyzed using the MCL (Markov clustering) software (Enright et al. 2002) and selected by having inflation value of 2.0 and e-value cutoff of 10^-30^. After removal of sequences < 120 bp, each of the clusters with at least 15 ingroup (Pooideae) sequences were aligned using MAFFT v7.407 (Katoh and Standley 2013). Following the removal of poorly aligned regions by Phyutility v2.2 (Smith and Dunn 2008) with “-clean 0.1”, a total of 43,085 preliminary gene trees were reconstructed using FastTree (Price et al. 2009). Terminal branches longer than 1.5 times of (absolute cutoff) or at least ten times longer than their sisters (relative cutoff) were then removed. To reduce putative cDNA isoforms from transcriptome datasets, among multiple sequences that formed a terminal clade of sequences from a single species, the sequence with the most unambiguous characters in the alignment was retained for gene tree construction. To prevent the excessive sequences in a cluster identified by MCL (Enright et al. 2002), the relatively long branch (> 2 times to its sister group) were used to divide the homologs in a cluster following the scripts of Yang et al. (2015, 2018), resulting in 3,911 subtrees representing additional homologous gene groups. Then, the sequences in each subtree were realigned, trimmed (with absolute cutoff and relative cutoff as mentioned above) and reconstructed a new gene tree. The processes were repeated until there were no relatively long branch tips in each gene tree. Finally, 45,722 of aligned homolog groups were used to reconstruct final ML gene trees by using RAxML v7.2 with GTRCAT model.

Afterwards, we performed tree reconciliation analysis by comparing gene trees with the reference (species) tree (supplementary fig. S23) as described in previous studies (Huang et al. 2016; Xiang et al. 2017; Ren et al. 2018; Leebens-Mack et al. 2019). The number of gene duplications was counted at each MRCA node by Tree2GD (https://github.com/Dee-chen/Tree2gd) with ≥ 50% bootstrap of the divergence of two subclades.

The synonymous substitution rate (Ks) for paralogs in a specific taxon was calculated as described previously (Leebens-Mack et al. 2019; Zhang et al. 2020b). Paralogs in a species were identified from all-by-all BLASTP using DIAMOND v0.9.21 (Buchfink et al. 2015) with e-value cut off of 10^-10^; then amino acid sequences with identity ≥ 50% and alignment length ≥ 100 amino acids were retained and were aligned by MAFFT v7.407 (Katoh and Standley 2013). The amino acid sequences in alignments were converted into nucleotide sequences using PAL2NAL (Suyama et al. 2006). The Ks value of each gene pair was estimated using the KaKs_calculator v2.0 (Zhang et al. 2006) with the maximum likelihood method. The distances from pairwise Ks values were clustered to construct a tentative tree (Tiley et al. 2018) and the Ks of each node in the tree (represents the duplication event) was calculated to reduce the effect of multi-copy genes on the Ks distribution (Maere et al. 2005; Vanneste et al. 2012; Tiley et al. 2018). Ks values > 2 were not used in subsequent analyses to avoid the effect of saturation. The number of peaks in Ks distribution was auto-optimized by mixtools v1.2.0 (Benaglia et al. 2009) and the peaks of Ks were also identified by a mixture model in mixtools v1.2.0 (Benaglia et al. 2009), with the median value of each peak calculated as support of WGD. Orthologous gene pairs were identified using DIAMOND v0.9.21 with the reciprocal best hit (RBH) between two species that sharing a common WGD and between one of these species and their outgroup as performed previously (Li et al. 2015b; Li and Barker 2020). The sequences were aligned for calculation of the Ks value following the same method as described above. The median Ks value of orthologs was retrieved and used as an estimate of the age of speciation event and then compared with the Ks value of putative WGD as mentioned above to assess whether the WGD occurred before or after the speciation event (Leebens-Mack et al. 2019; Li and Barker 2020).

Moreover, gene collinearity in assembled genome was analyzed by MCScanX (Wang et al. 2012), then the intersection between syntenic gene pairs and duplicates derived from the phylogenetic method of gene duplication cluster is used as evidence of genome doubling.

## 4.8 Molecular Evolution of *AP1*/*FUL* and *CBF* Homologs

To reconstruct the evolutionary history of *AP1*/*FUL* homologs, we selected a total of 46 species (24 transcriptomes, 22 genomes) including representatives of Poaceae subfamilies, other Poales families, other orders across monocots, eudicots, and the early-divergent *Amborella* (supplementary table S13). We retrieved candidate *AP1*/*FUL* homologs of all representatives by using HMMER v3.1 (Mistry et al. 2013) with the entry domain (PF01486.17) and default settings. To identify *CBF* homologs, which are members of a subclade of the AP2/ERF superfamily containing the AP2 domain, we performed a BLASTP search (e-value cut off =10^-5^) to favor genes that are more closely related to *CBF*. The retrieved protein sequences were aligned using MAFFT v7.407 (Katoh and Standley 2013) and then trimmed using trimAl v1.4 (Capella-Gutiérrez et al. 2009). Initial phylogenetic trees of putative *FUL* and *CBF* homologs were reconstructed using the ML method implemented in FastTree (Price et al. 2009). Next, the subclade containing members of *AP1*/*FUL*-like or *CBF2A* genes and members of their sister clades were extracted. Finally, the corresponding nucleotide sequences were used to reconstruct the phylogeny using IQ-TREE (Nguyen et al. 2015). In addition, we used MCScanX (Wang et al. 2012) and location of genes on the chromosome to confirm the type of duplication.

# 5. References

Alfaro ME, Santini F, Brock C, Alamillo H, Dornburg A, Rabosky DL, Carnevale G, Harmon LJ. 2009. Nine exceptional radiations plus high turnover explain species diversity in jawed vertebrates. *Proc Natl Acad Sci U S A.* 106(32):13410-13414.

Arrigo N, Felber F, Parisod C, Buerki S, Alvarez N, David J, Guadagnuolo R. 2010. Origin and expansion of the allotetraploid *Aegilops geniculata*, a wild relative of wheat. *New Phytol.* 187(4):1170-1180.

Avni R, Nave M, Barad O, Baruch K, Twardziok S, Gundlach H, Hale I, Mascher M, Spannagl M, Wiebe K, et al. 2017. Wild emmer genome architecture and diversity elucidate wheat evolution and domestication. *Science* 357:93-97.

Beaulieu JM, O'Meara BC, Donoghue MJ. 2013. Identifying hidden rate changes in the evolution of a binary morphological character: The evolution of plant habit in campanulid angiosperms. *Syst Biol.* 62(5):725-737.

Benaglia T, Chauveau D, Hunter D, Young D. 2009. mixtools: An R package for analyzing finite mixture models. *J. Stat. Softw.* 32(6):1-29.

Bouckaert R, Heled J, Kühnert D, Vaughan T, Wu C-H, Xie D, Suchard MA, Rambaut A, Drummond AJ. 2014. BEAST 2: A software platform for bayesian evolutionary analysis. *PLoS Comput. Biol.* 10(4):e1003537.

Buchfink B, Xie C, Huson DH. 2015. Fast and sensitive protein alignment using DIAMOND. *Nat Methods* 12(1):59-60.

Cannarozzi G, Plaza-Wüthrich S, Esfeld K, Larti S, Wilson YS, Girma D, de Castro E, Chanyalew S, Blösch R, Farinelli L, et al. 2014. Genome and transcriptome sequencing identifies breeding targets in the orphan crop tef (*Eragrostis tef*). *BMC Genom*. 15(1):581.

Capella-Gutiérrez S, Silla-Martínez JM, Gabaldón T. 2009. trimAl: A tool for automated alignment trimming in large-scale phylogenetic analyses. *Bioinformatics* 25(15):1972-1973.

Christin P-A, Spriggs E, Osborne CP, Strömberg CAE, Salamin N, Edwards EJ. 2014. Molecular dating, evolutionary rates, and the age of the grasses. *Syst Biol.* 63(2):153-165.

Drummond AJ, Suchard MA, Xie D, Rambaut A. 2012. Bayesian Phylogenetics with BEAUti and the BEAST 1.7. *Mol Biol Evol.* 29(8):1969-1973.

Ebersberger I, Strauss S, von Haeseler A. 2009. HaMStR: Profile hidden markov model based search for orthologs in ESTs. *BMC Evol Biol.* 9(1):157.

Edgar RC. 2004. MUSCLE: Multiple sequence alignment with high accuracy and high throughput. *Nucleic Acids Res.* 32(5):1792-1797.

Enright AJ, Van Dongen S, Ouzounis CA. 2002. An efficient algorithm for large-scale detection of protein families. *Nucleic Acids Res.* 30(7):1575-1584.

Fu L, Niu B, Zhu Z, Wu S, Li W. 2012. CD-HIT: Accelerated for clustering the next-generation sequencing data. *Bioinformatics* 28(23):3150-3152.

Glémin S, Scornavacca C, Dainat J, Burgarella C, Viader V, Ardisson M, Sarah G, Santoni S, David J, Ranwez V. 2019. Pervasive hybridizations in the history of wheat relatives. *Sci Adv.* 5(5):eaav9188.

Grabherr MG, Haas BJ, Yassour M, Levin JZ, Thompson DA, Amit I, Adiconis X, Fan L, Raychowdhury R, Zeng Q, et al. 2011. Full-length transcriptome assembly from RNA-Seq data without a reference genome. *Nat Biotechnol.* 29(7):644-652.

Haas BJ, Papanicolaou A, Yassour M, Grabherr M, Blood PD, Bowden J, Couger MB, Eccles D, Li B, Lieber M, et al. 2013. De novo transcript sequence reconstruction from RNA-Seq using the Trinity platform for reference generation and analysis. *Nat Protoc* 8(8):1494-1512.

Huang C-H, Zhang C, Liu M, Hu Y, Gao T, Qi J, Ma H. 2016. Multiple polyploidization events across Asteraceae with two nested events in the early history revealed by nuclear phylogenomics. *Mol Biol Evol.* 33(11):2820-2835.

Iles WJD, Smith SY, Gandolfo MA, Graham SW. 2015. Monocot fossils suitable for molecular dating analyses. *Bot J Linn Soc.* 178(3):346-374.

Katoh K, Standley DM. 2013. MAFFT multiple sequence alignment software version 7: Improvements in performance and usability. *Mol Biol Evol.* 30(4):772-780.

Leebens-Mack JH, Barker MS, Carpenter EJ, Deyholos MK, Gitzendanner MA, Graham SW, Grosse I, Li Z, Melkonian M, Mirarab S, et al. 2019. One thousand plant transcriptomes and the phylogenomics of green plants. *Nature* 574(7780):679-685.

Li L-F, Liu B, Olsen KM, Wendel JF. 2015a. A re-evaluation of the homoploid hybrid origin of *Aegilops tauschii*, the donor of the wheat D-subgenome. *New Phytol.* 208(1):4-8.

Li L, Stoeckert CJ, Roos DS. 2003. OrthoMCL: Identification of ortholog groups for eukaryotic genomes. *Genome Res.* 13(9):2178-2189.

Li Z, Baniaga AE, Sessa EB, Scascitelli M, Graham SW, Rieseberg LH, Barker MS. 2015b. Early genome duplications in conifers and other seed plants. *Sci Adv.* 1(10):e1501084.

Li Z, Barker MS. 2020. Inferring putative ancient whole-genome duplications in the 1000 Plants (1KP) initiative: Access to gene family phylogenies and age distributions. *GigaScience* 9(2):giaa004.

Maere S, De Bodt S, Raes J, Casneuf T, Van Montagu M, Kuiper M, Van de Peer Y. 2005. Modeling gene and genome duplications in eukaryotes. *Proc Natl Acad Sci U S A.* 102(15):5454-5459.

Magallón S, Gómez-Acevedo S, Sánchez-Reyes LL, Hernández-Hernández T. 2015. A metacalibrated time-tree documents the early rise of flowering plant phylogenetic diversity. *New Phytol.* 207(2):437-453.

Marcussen T, Sandve S, Heier L, Spannagl M, Pfeifer M, Jakobsen K, Steuernagel B, Mayer K, Olsen O-A, Rogers J, et al. 2014. Ancient hybridizations among the ancestral genomes of bread wheat. *Science* 345:1250092.

Mirarab S, Reaz R, Bayzid MS, Zimmermann T, Swenson MS, Warnow T. 2014. ASTRAL: Genome-scale coalescent-based species tree estimation. *Bioinformatics* 30(17):i541-i548.

Mistry J, Finn RD, Eddy SR, Bateman A, Punta M. 2013. Challenges in homology search: HMMER3 and convergent evolution of coiled-coil regions. *Nucleic Acids Res.* 41(12):e121-e121.

Moore BR, Höhna S, May MR, Rannala B, Huelsenbeck JP. 2016. Critically evaluating the theory and performance of Bayesian analysis of macroevolutionary mixtures. *Proc Natl Acad Sci U S A.* 113(34):9569.

Nguyen L-T, Schmidt HA, von Haeseler A, Minh BQ. 2015. IQ-TREE: A fast and effective stochastic algorithm for estimating maximum-likelihood phylogenies. *Mol Biol Evol.* 32(1):268-274.

Peng Z, Lu Y, Li L, Zhao Q, Feng Q, Gao Z, Lu H, Hu T, Yao N, Liu K, et al. 2013. The draft genome of the fast-growing non-timber forest species moso bamboo (*Phyllostachys heterocycla*). *Nat Genet.* 45:456-461.

Pimentel M, Escudero M, Sahuquillo E, Minaya MÁ, Catalán P. 2017. Are diversification rates and chromosome evolution in the temperate grasses (Pooideae) associated with major environmental changes in the Oligocene-Miocene? *PeerJ* 5:e3815.

Prasad V, Strömberg C, Leaché A, Samant B, Patnaik R, Tang L, Mohabey D, Ge S, Sahni A. 2011. Late Cretaceous origin of the rice tribe provides evidence for early diversification in Poaceae. *Nat Commun.* 2:480.

Price MN, Dehal PS, Arkin AP. 2009. FastTree: Computing large minimum evolution trees with profiles instead of a distance matrix. *Mol Biol Evol.* 26(7):1641-1650.

Rabosky DL, Grundler M, Anderson C, Title P, Shi JJ, Brown JW, Huang HT, Larson JG. 2014. BAMMtools: An R package for the analysis of evolutionary dynamics on phylogenetic trees. *Methods Ecol Evol.* 5(7):701-707.

Rambaut A, Drummond AJ, Xie D, Baele G, Suchard MA. 2018. Posterior summarization in bayesian phylogenetics using Tracer 1.7. *Syst Biol.* 67(5):901-904.

Ren R, Wang H, Guo C, Zhang N, Zeng L, Chen Y, Ma H, Qi J. 2018. Widespread whole genome duplications contribute to genome complexity and species diversity in angiosperms. *Mol Plant* 11(3):414-428.

Salamini F, Özkan H, Brandolini A, Schäfer-Pregl R, Martin W. 2002. Genetics and geography of wild cereal domestication in the near east. *Nat Rev Genet.* 3(6):429-441.

Sauquet H, Ho SYW, Gandolfo MA, Jordan GJ, Wilf P, Cantrill DJ, Bayly MJ, Bromham L, Brown GK, Carpenter RJ, et al. 2011. Testing the impact of calibration on molecular divergence times using a fossil-rich group: The case of *Nothofagus* (Fagales). *Syst Biol.* 61(2):289-313.

Schubert M, Marcussen T, Meseguer AS, Fjellheim S. 2019. The grass subfamily Pooideae: Cretaceous-Palaeocene origin and climate-driven Cenozoic diversification. *Glob Ecol Biogeogr.* 28(8):1168-1182.

Smith SA, Brown JW, Walker JF. 2018. So many genes, so little time: A practical approach to divergence-time estimation in the genomic era. *PloS One* 13(5):e0197433.

Smith SA, Dunn CW. 2008. Phyutility: A phyloinformatics tool for trees, alignments and molecular data. *Bioinformatics* 24(5):715-716.

Smith SA, O’Meara BC. 2012. treePL: Divergence time estimation using penalized likelihood for large phylogenies. *Bioinformatics* 28(20):2689-2690.

Soreng RJ, Davis JI. 2000. Phylogenetic structure in Poaceae subfamily Pooideae as inferred from molecular and morphological characters: misclassification versus reticulation. In. Grasses: systematics and evolution: CSIRO. p. 61-74.

Soreng RJ, Davis JI. 1998. Phylogenetics and character evolution in the grass family (Poaceae): Simultaneous analysis of morphological and chloroplast DNA restriction site character sets. *Bot Rev.* 64(1):1-85.

Soreng RJ, Peterson PM, Romaschenko K, Davidse G, Teisher JK, Clark LG, Barberá P, Gillespie LJ, Zuloaga FO. 2017. A worldwide phylogenetic classification of the Poaceae (Gramineae) II: An update and a comparison of two 2015 classifications. *J Syst Evol.* 55(4):259-290.

Soreng RJ, Peterson PM, Romaschenko K, Davidse G, Zuloaga FO, Judziewicz EJ, Filgueiras TS, Davis JI, Morrone O. 2015. A worldwide phylogenetic classification of the Poaceae (Gramineae). *J Syst Evol.* 53(2):117-137.

Spriggs EL, Christin P-A, Edwards EJ. 2014. C4 photosynthesis promoted species diversification during the Miocene grassland expansion. *PloS one* 9(5):e97722.

Stamatakis A. 2006. RAxML-VI-HPC: Maximum likelihood-based phylogenetic analyses with thousands of taxa and mixed models. *Bioinformatics* 22(21):2688-2690.

Struck TH. 2014. TreSpEx-Detection of misleading signal in phylogenetic reconstructions based on tree information. *Evol Bioinform.* 10:51-67.

Suyama M, Torrents D, Bork P. 2006. PAL2NAL: Robust conversion of protein sequence alignments into the corresponding codon alignments. *Nucleic Acids Res.* 34:W609-W612.

Tiley GP, Barker MS, Burleigh JG. 2018. Assessing the performance of Ks plots for detecting ancient whole genome duplications. *Genome Biol Evol.* 10(11):2882-2898.

Vanneste K, Van de Peer Y, Maere S. 2012. Inference of genome duplications from age distributions revisited. *Mol Biol Evol.* 30(1):177-190.

Wang Y, Tang H, DeBarry JD, Tan X, Li J, Wang X, Lee T-h, Jin H, Marler B, Guo H, et al. 2012. MCScanX: A toolkit for detection and evolutionary analysis of gene synteny and collinearity. *Nucleic Acids Res.* 40(7):e49-e49.

Xiang Y, Huang C-H, Hu Y, Wen J, Li S, Yi T, Chen H, Xiang J, Ma H. 2017. Evolution of Rosaceae fruit types based on nuclear phylogeny in the context of geological times and genome duplication. *Mol Biol Evol.* 34(2):262-281.

Yang Y, Moore MJ, Brockington SF, Mikenas J, Olivieri J, Walker JF, Smith SA. 2018. Improved transcriptome sampling pinpoints 26 ancient and more recent polyploidy events in Caryophyllales, including two allopolyploidy events. *New Phytol.* 217(2):855-870.

Yang Y, Moore MJ, Brockington SF, Soltis DE, Wong GK-S, Carpenter EJ, Zhang Y, Chen L, Yan ZX, Xie YL, et al. 2015. Dissecting molecular evolution in the highly diverse plant clade Caryophyllales using transcriptome sequencing. *Mol Biol Evol.* 32(8):2001-2014.

Zeng L, Zhang Q, Sun R, Kong H, Zhang N, Ma H. 2014. Resolution of deep angiosperm phylogeny using conserved nuclear genes and estimates of early divergence times. *Nat Commun.* 5:4956.

Zhang C, Scornavacca C, Molloy EK, Mirarab S. 2020a. ASTRAL-Pro: Quartet-based species-tree inference despite paralogy. *Mol Biol Evol.* 37(11):3292-3307.

Zhang C, Zhang T, Luebert F, Xiang Y, Huang C-H, Hu Y, Rees M, Frohlich MW, Qi J, Weigend M, et al. 2020b. Asterid phylogenomics/phylotranscriptomics uncover morphological evolutionary histories and support phylogenetic placement for numerous whole genome duplications. *Mol Biol Evol.* 37(11):3188-3210.

Zhang Z, Li J, Zhao X-Q, Wang J, Wong GK-S, Yu J. 2006. KaKs_Calculator: Calculating Ka and Ks through model selection and model averaging. *Genom Proteom Bioinf.* 4(4):259-263.
