# Supplementary material for: Phylotranscriptomics Resolves the Phylogeny of Pooideae and Uncovers Factors for Their Adaptive Evolution
Source: Mol Biol Evol. 2022 Feb 3;39(2):msac026. doi: 10.1093/molbev/msac026 (PMC8844509; doi:10.1093/molbev/msac026)
Supplement: msac026_Supplementary_Data [file msac026_supplementary_data.zip › Sumplementary_figures.pdf]

Zhang et al.

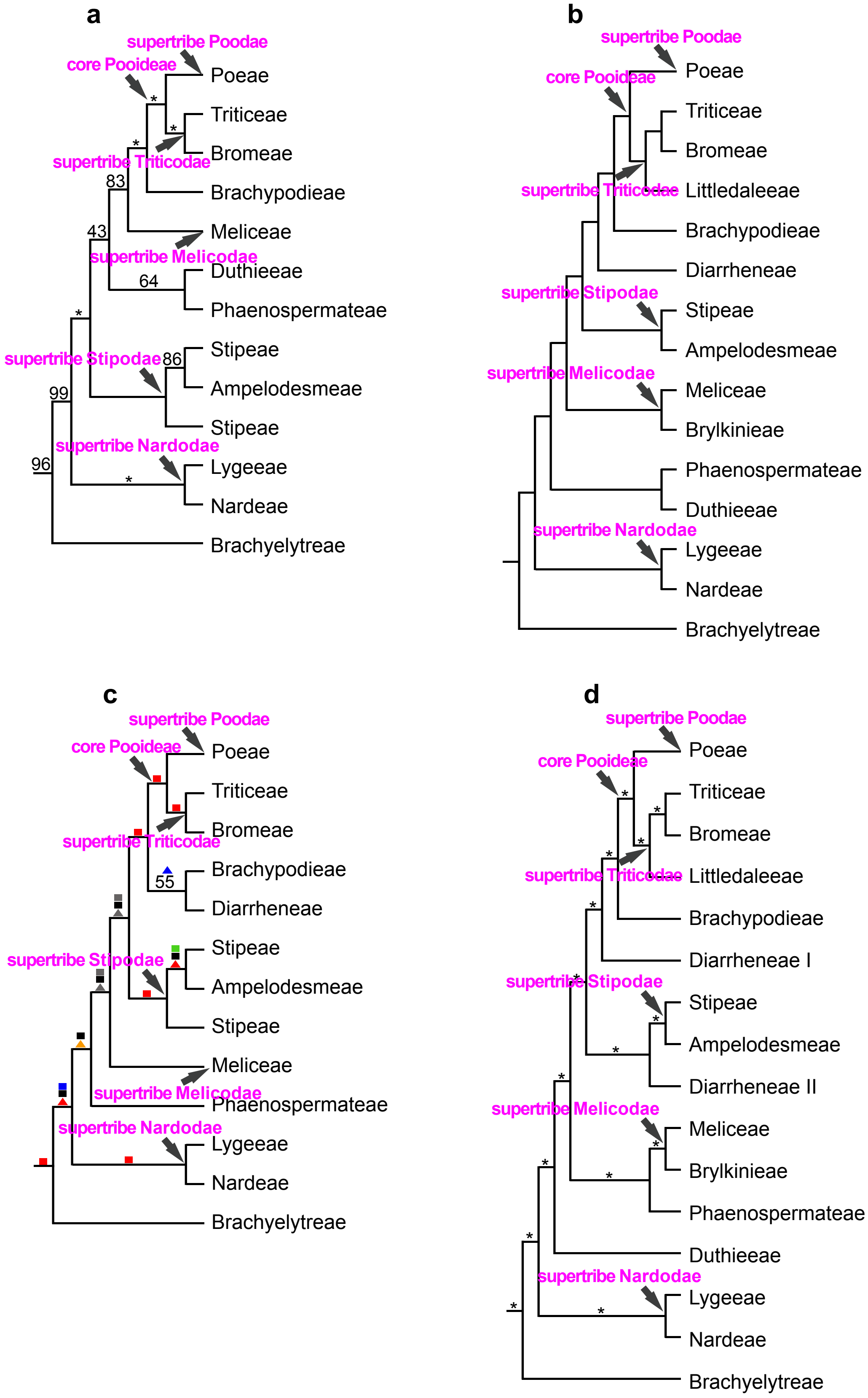

Figure S1 A comparison of Pooideae phylogenies at the tribe level from previous reports and this study.

(a) The topology is summarized from GPWGII (2012) (three chloroplast markers, 49 species). Bootstrap values (BS) are shown on the tree. The BS = 100% is marked with *. (b) The topology is summarized from Soreng et al. (2017) (two chloroplast markers) without BS. (c) The topology is summarized from Saarela et al. (2018) (all plastome, 64 species). The BS = 100% in this tree is marked as black square. The BS = 100% in all three-gene and complete plastome trees is marked as red square. The BS = 100% in all complete plastome trees is marked as blue square. The BS = 90-100% in all complete plastome trees is marked as green square. The BS = 80-100% in all complete plastome trees is marked as gray square. The colorful triangles represent BS ≥ 50% in all 14 trees (red); BS ≥ 50% in 12-13 trees (yellow); BS ≥ 50% in 10-11 trees (gray); BS ≥ 50% in 1-3 trees (blue). (d) The topology is summarized from this study based on the astral tree of five gene sets. Five trees with 100% BS of the same node is marked with asterisk.

Zhang et al.

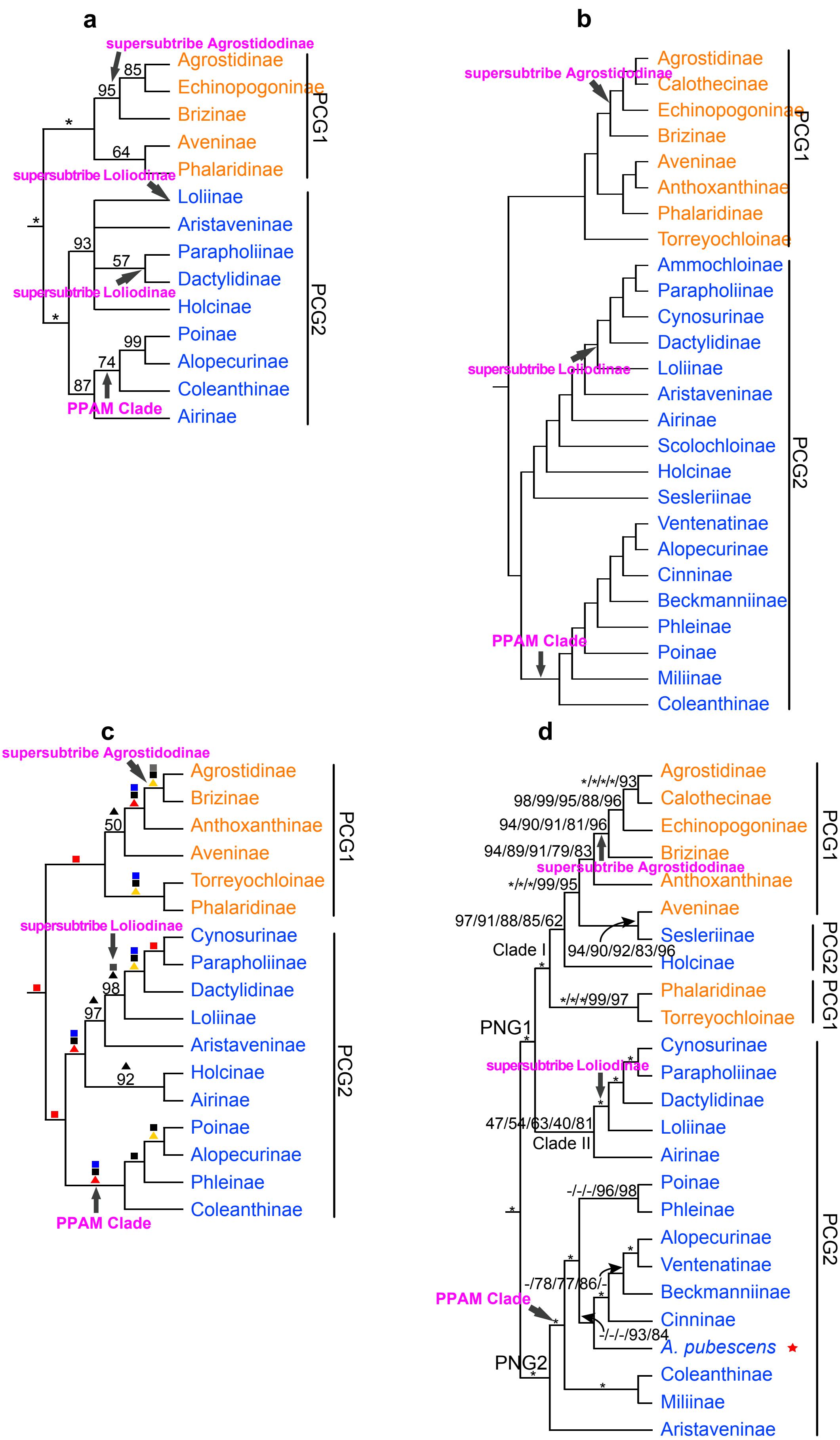

Figure S2 A comparison of Pooideae phylogenies at the subtribe level from previous reports and this study.

The genera of Poeae chloroplast group 1 (PCG1) and Poeae chloroplast group 2 (PCG2) are presented as orange and blue, respectively. PNG1 and PNG2 represent Poeae nuclear group 1 and Poeae nuclear group 2 in this study, respectively. The topologies (a-d) are from the references/study as the same as supplementary fig. S1. BS values are shown on the branches (missing support with a short dash) otherwise 100% BS is marked with asterisk. *Avenula pubescens* with unplaced position in previous studies is marked with red star.

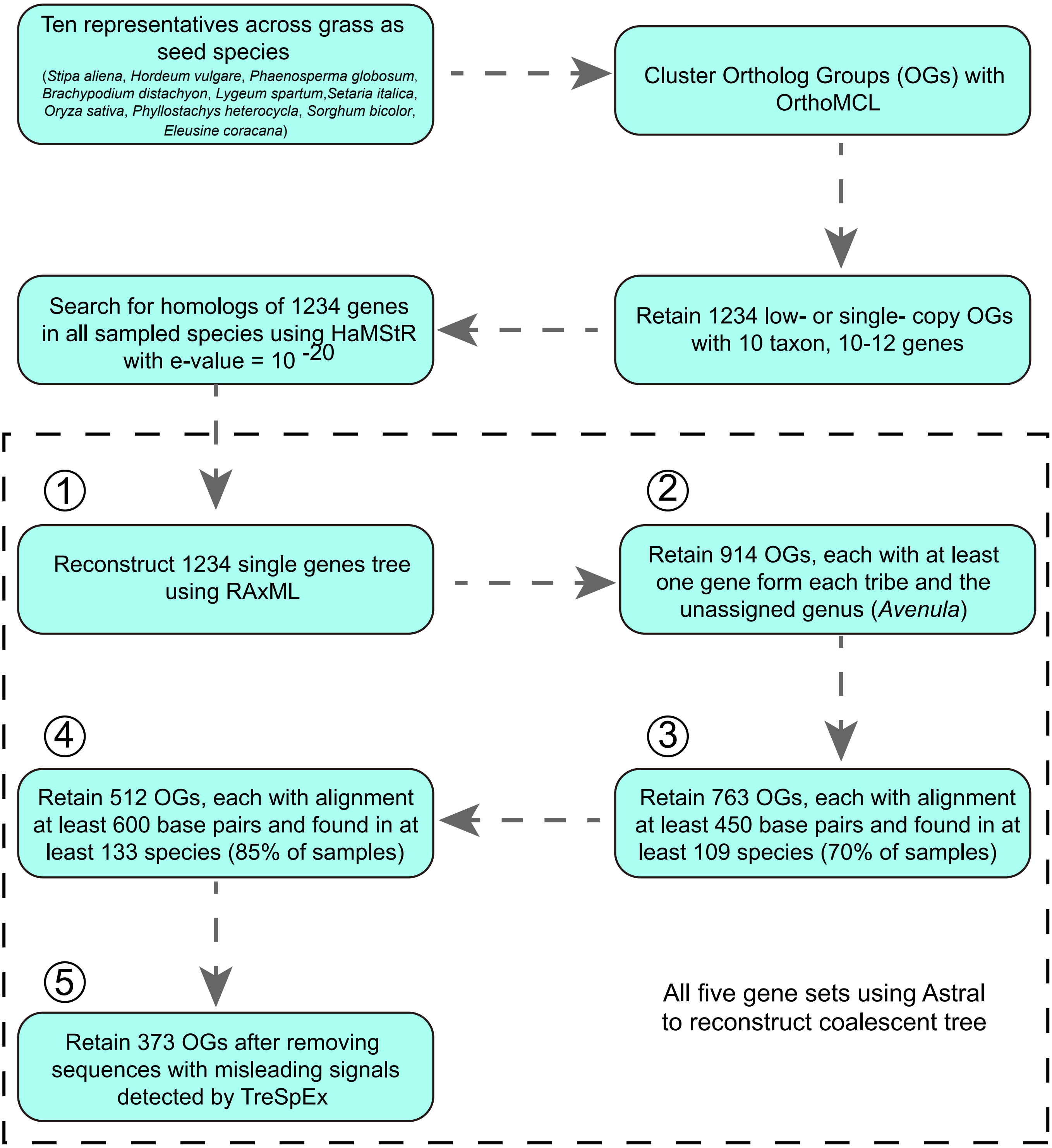

Figure S3 A flow chart of procedures for ortholog identification and gene-set selection. Within the dashed rectangle, the areas labeled with numbers 1-5 provide information of the 1234-, 914-, 763-, 512- and 373 OGs, respectively.

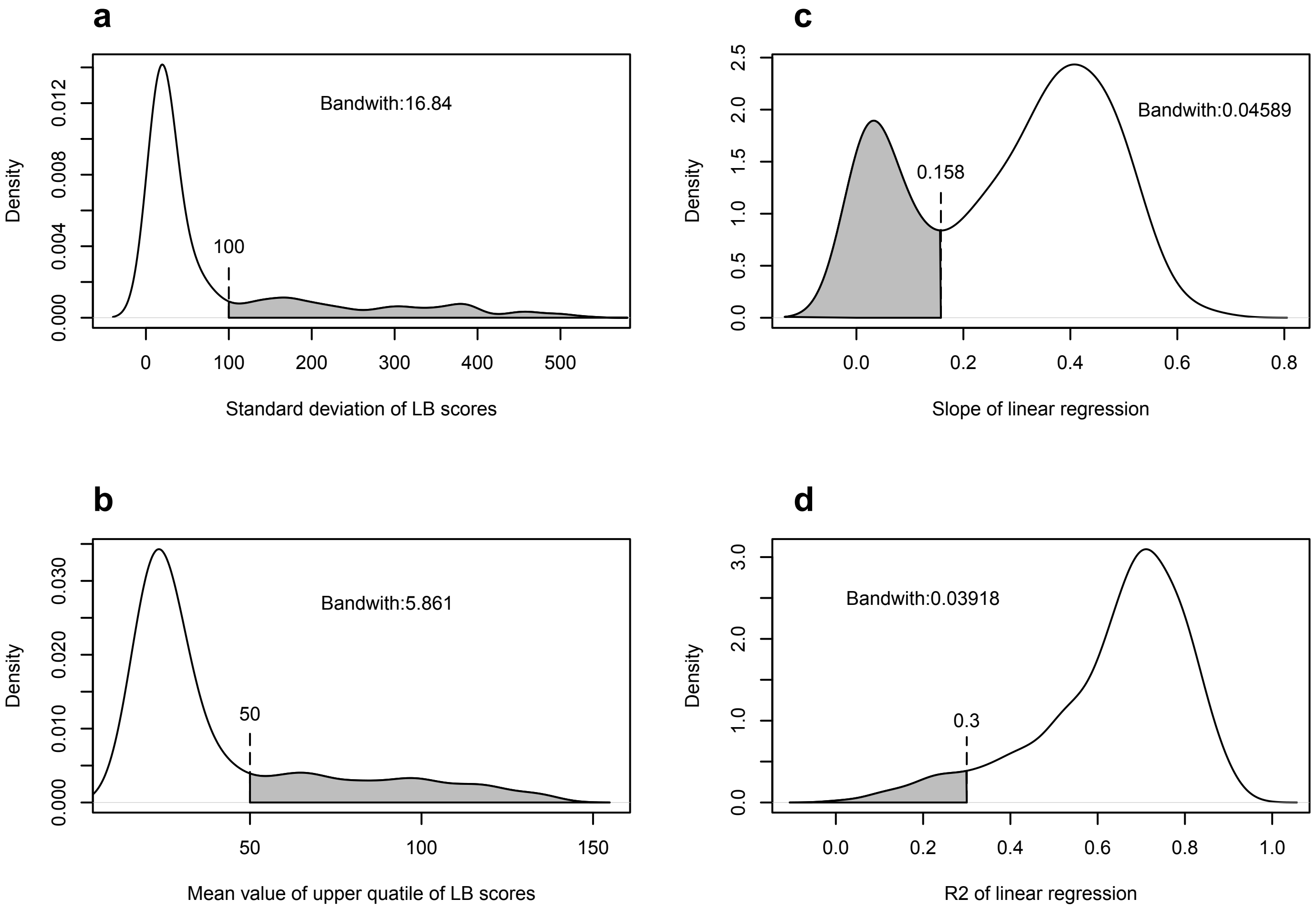

Figure S4 Density plots of long-branch (LB) and saturation indices of 373 OGs.
Long-branch attraction (a, b) and saturation of partitions (c, d) were detected by TrSpEx. Thresholds are marked by dashed lines and the corresponding values are shown above the lines. Gray regions contain genes with long branch (a and b) and saturation (c and d).

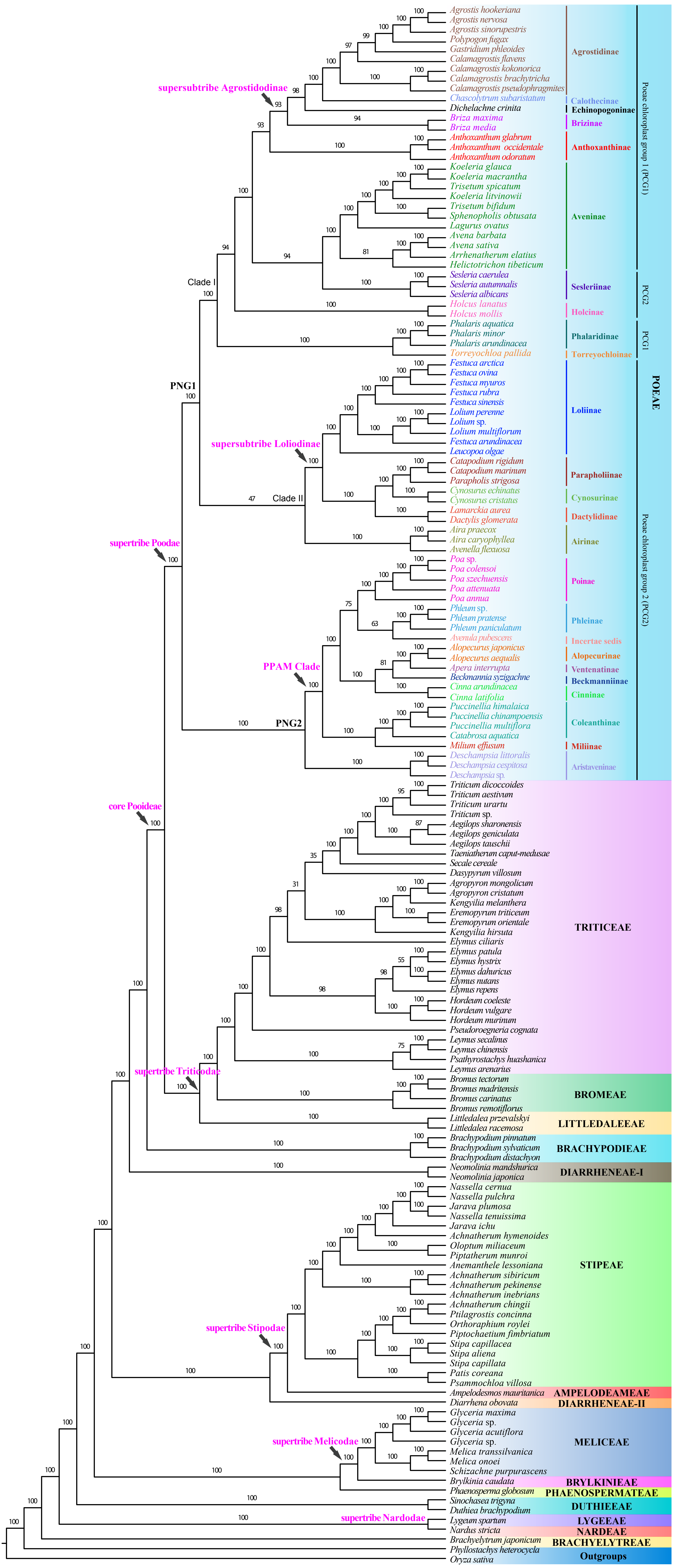

Figure S5 A phylogeny inferred from 1234 OGs by ASTRAL.
Numbers at nodes indicate the bootstrap support (BS). Tribes (uppercase) and subtribes of Poeae are highlighted with different colors, with their names shown on the right. Major lineages (supertribe, supersubtribe or the core Pooideae) are indicated with pink color beside the arrow.

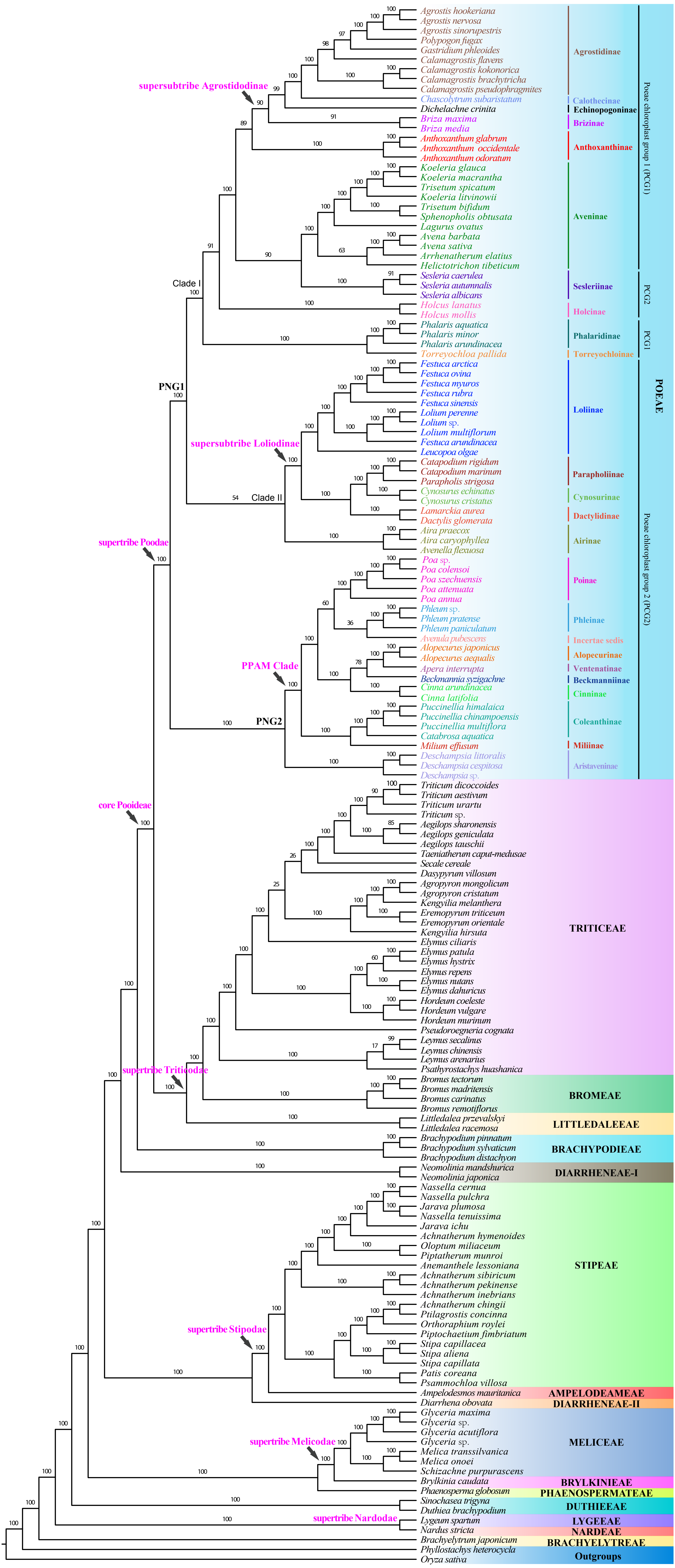

Figure S6 A phylogeny inferred from 914 OGs by ASTRAL.
Numbers at nodes indicate the bootstrap support (BS). Tribes (uppercase) and subtribes of Poeae are highlighted with different colors, with their names shown on the right. Major lineages (supertribe, supersubtribe or the core Pooideae) are indicated with pink color beside the arrow.

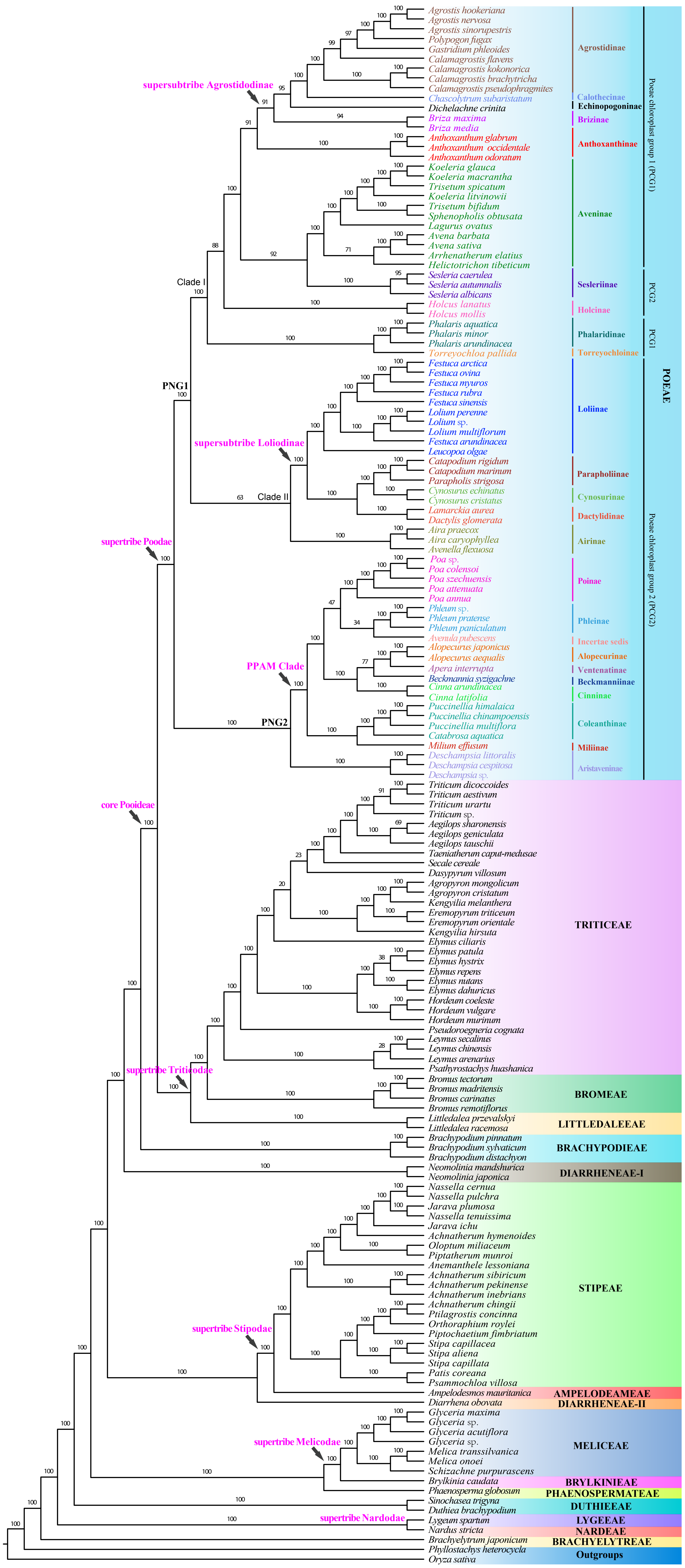

Figure S7 A phylogeny inferred from 763 OGs by ASTRAL.
Numbers at nodes indicate the bootstrap support (BS). Tribes (uppercase) and subtribes of Poeae are highlighted with different colors, with their names shown on the right. Major lineages (supertribe, supersubtribe or the core Pooideae) are indicated with pink color beside the arrow.

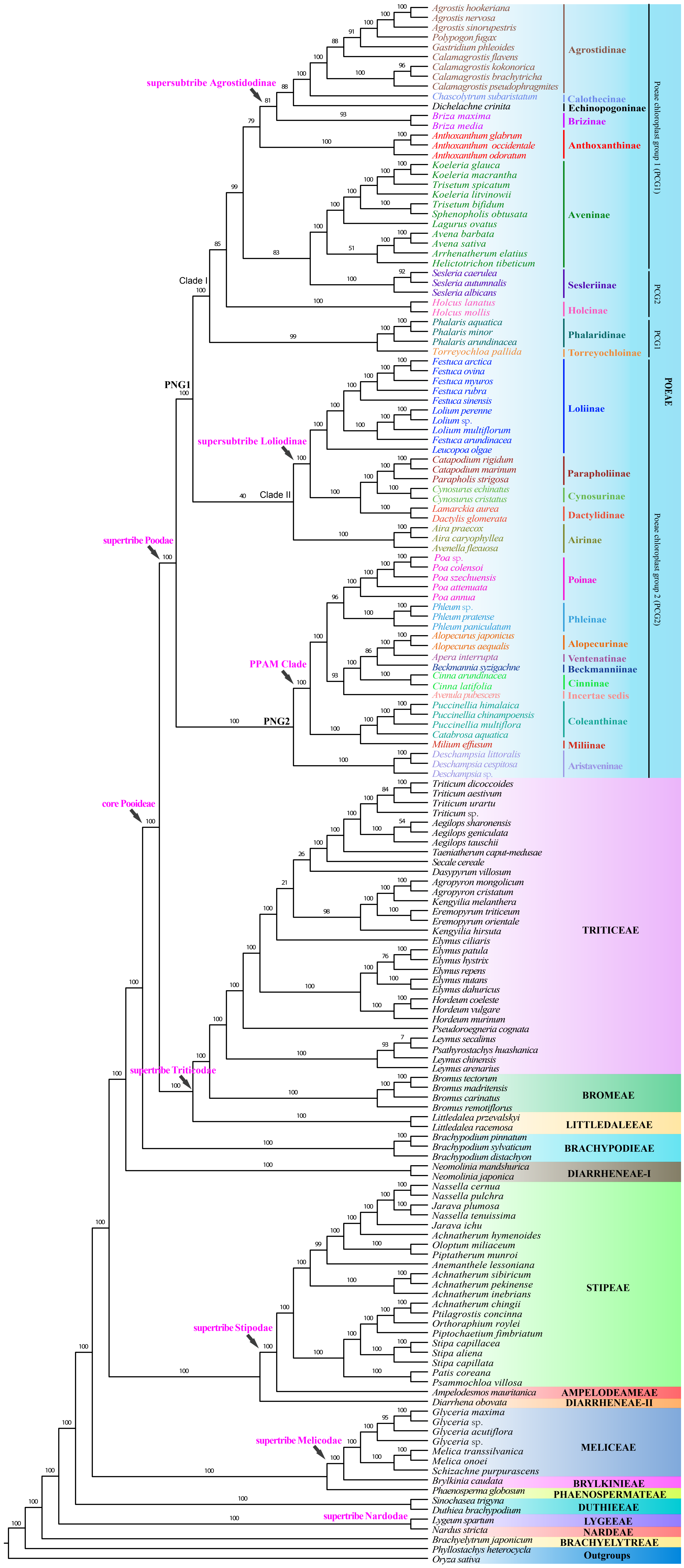

Figure S8 A phylogeny inferred from 512 OGs by ASTRAL.
Numbers at nodes indicate the bootstrap support (BS). Tribes (uppercase) and subtribes of Poeae are highlighted with different colors, with their names shown on the right. Major lineages (supertribe, supersubtribe or the core Pooideae) are indicated with pink color beside the arrow.

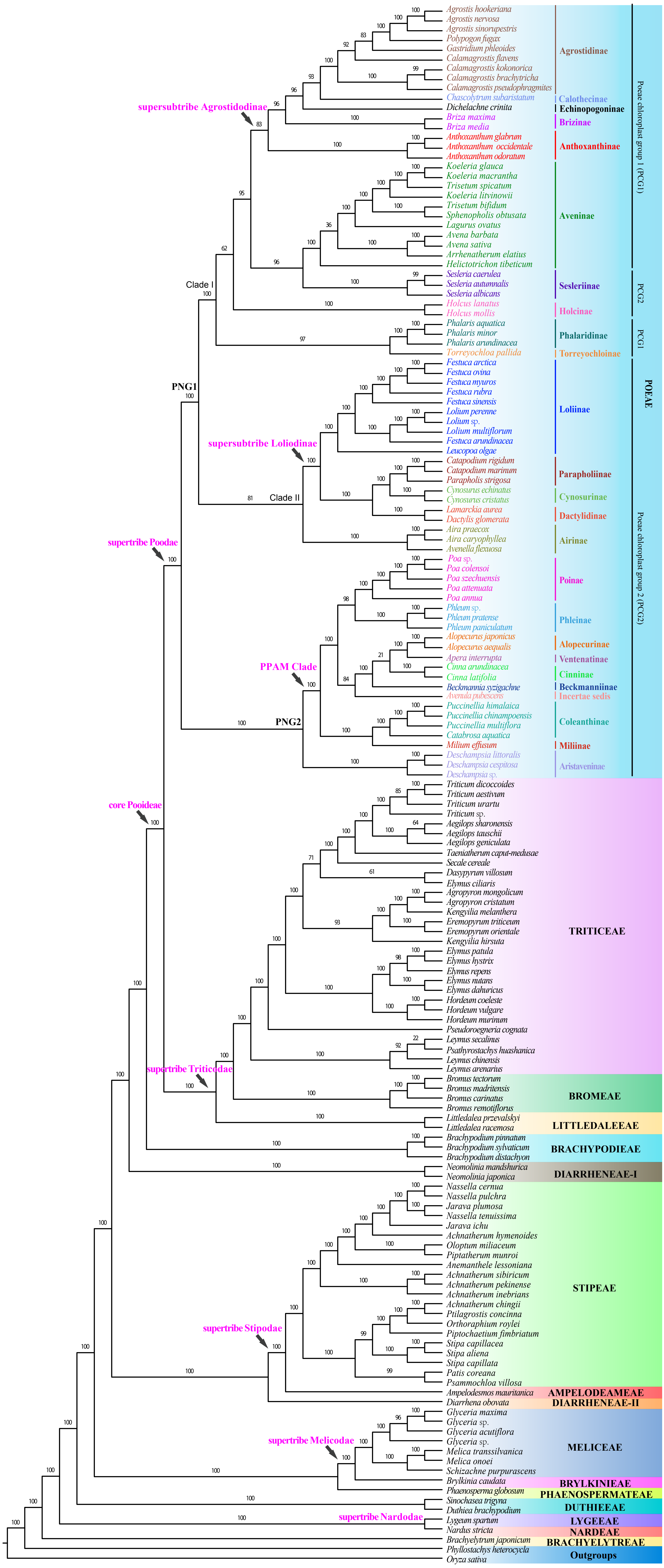

Figure S9 A phylogeny inferred from 373 OGs by ASTRAL.
Numbers at nodes indicate the bootstrap support (BS). Tribes (uppercase) and subtribes of Poeae are highlighted with different colors, with their names shown on the right. Major lineages (supertribe, supersubtribe or the core Pooideae) are indicated with pink color beside the arrow.

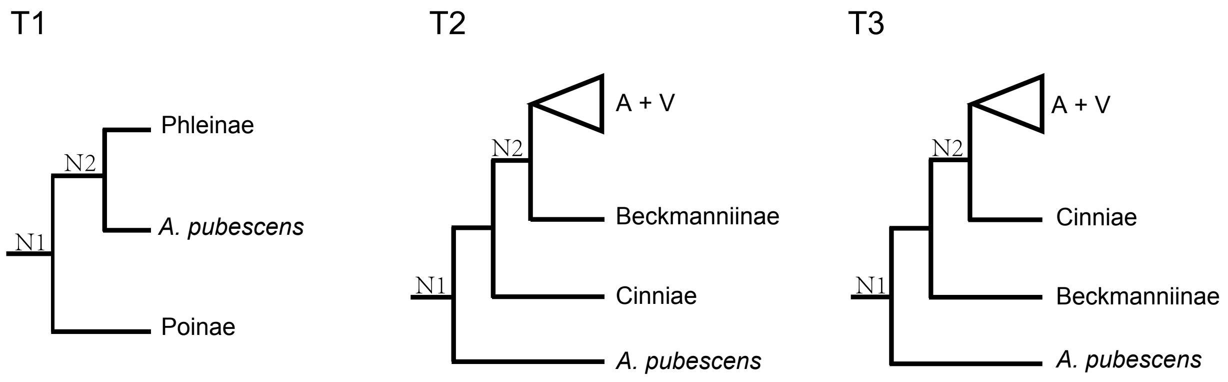

**Gene sets**	**1234OGs**	**914OGs**	**763OGs**	**512OGs**	**373OGs**
**Topology**	T1	T1	T1	T2	T3
N1	75	60	47	93	84
N2	63	36	34	86	21

BS (yellow) < 65 (magenta) > 80

A+V: Alopecurinae + Ventenatinae

Figure S10 Alternative topology of *Avenula pubescens* from five trees.

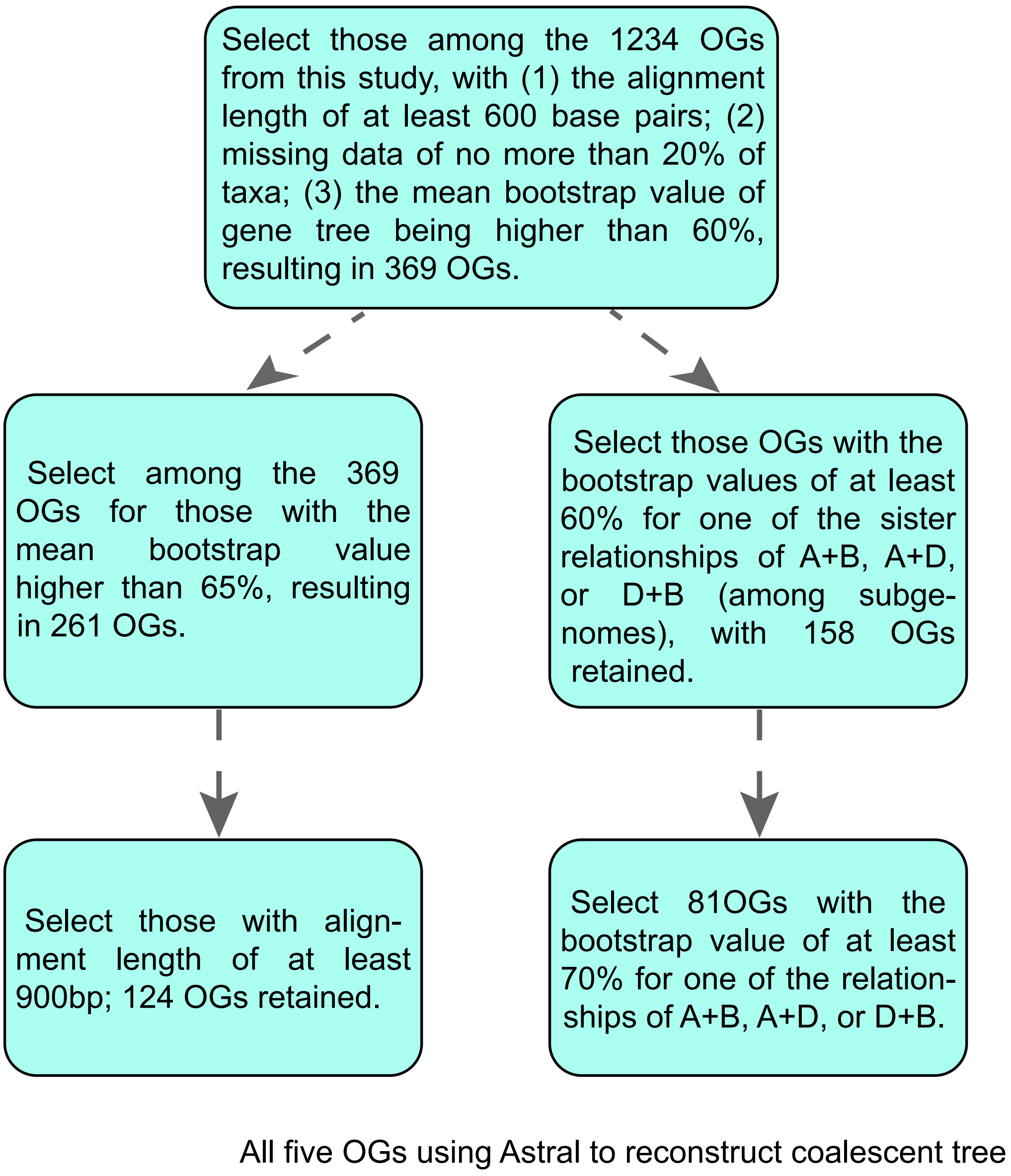

Figure S11 A flow chart of gene-set selection for analyses of the phylogenetic relationships of subgenomes of wheat and its relatives.

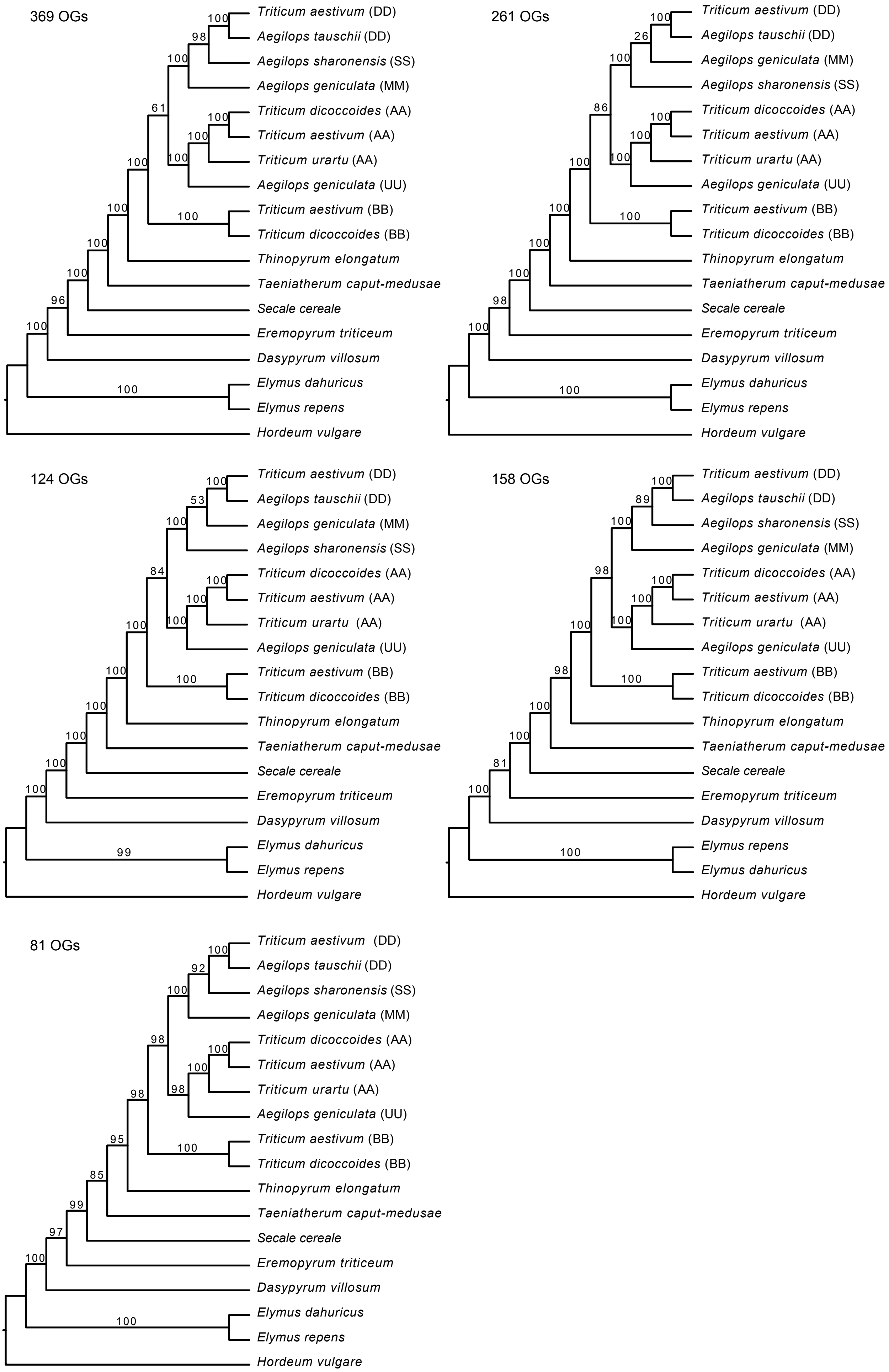

Figure S12 The phylogenetic trees of wheat relatives inferred from five OGs by ASTRAL. The flow chart of the selection of five OGs is indicated in supplementary fig. S11. Numbers at nodes indicate bootstrap support (BS) values. Subgenomes of *Triticum* and *Aegilops* are marked in the parentheses.

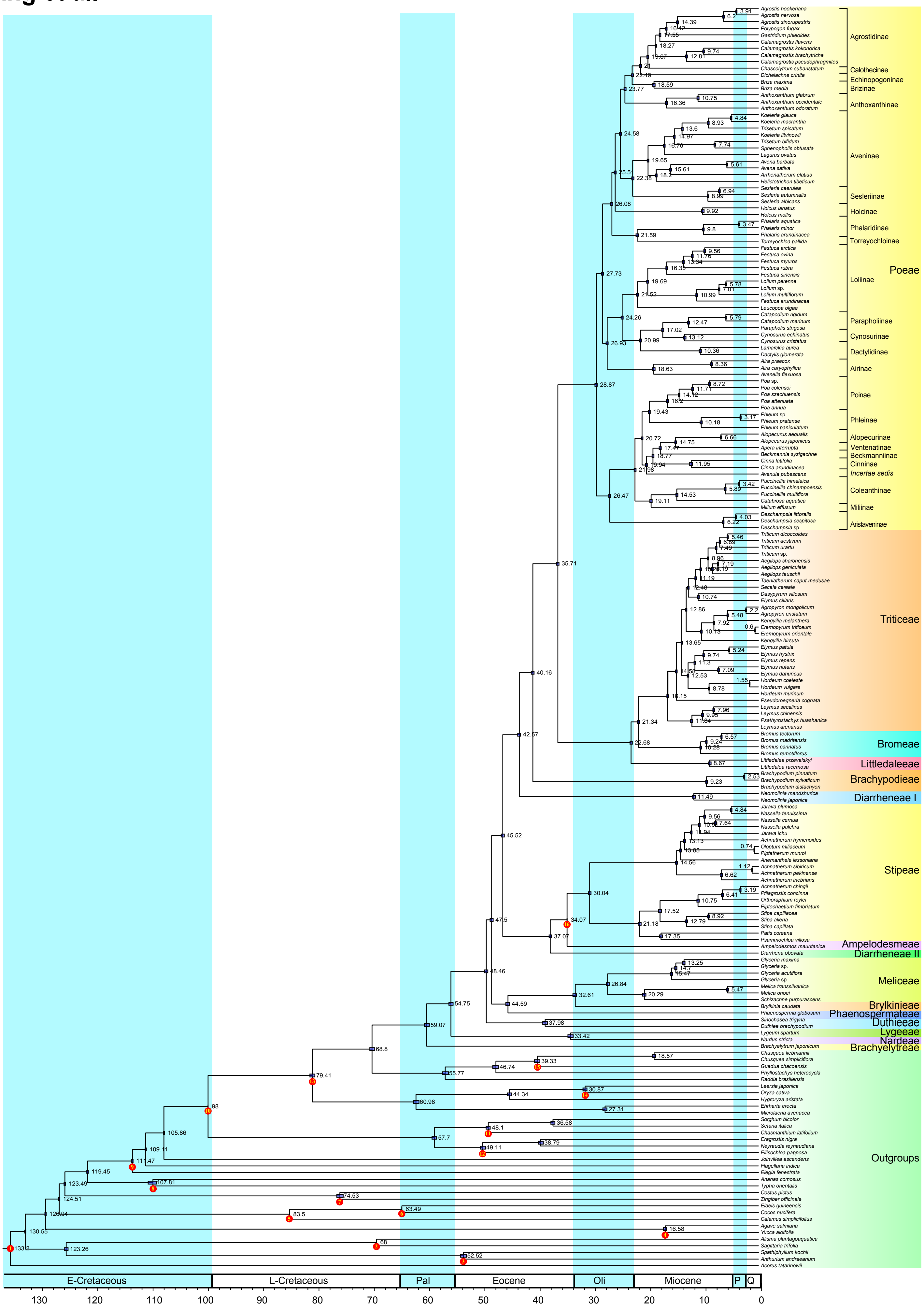

Figure S13 Divergence times of Pooideae inferred by treePL with calibration 1 and 3. The red circles with numbers indicate the fossil calibrations corresponding to those in supplementary table S4. Calibrations 1 and 3 indicate the strategies of exclusion and inclusion of fossil N13, respectively.

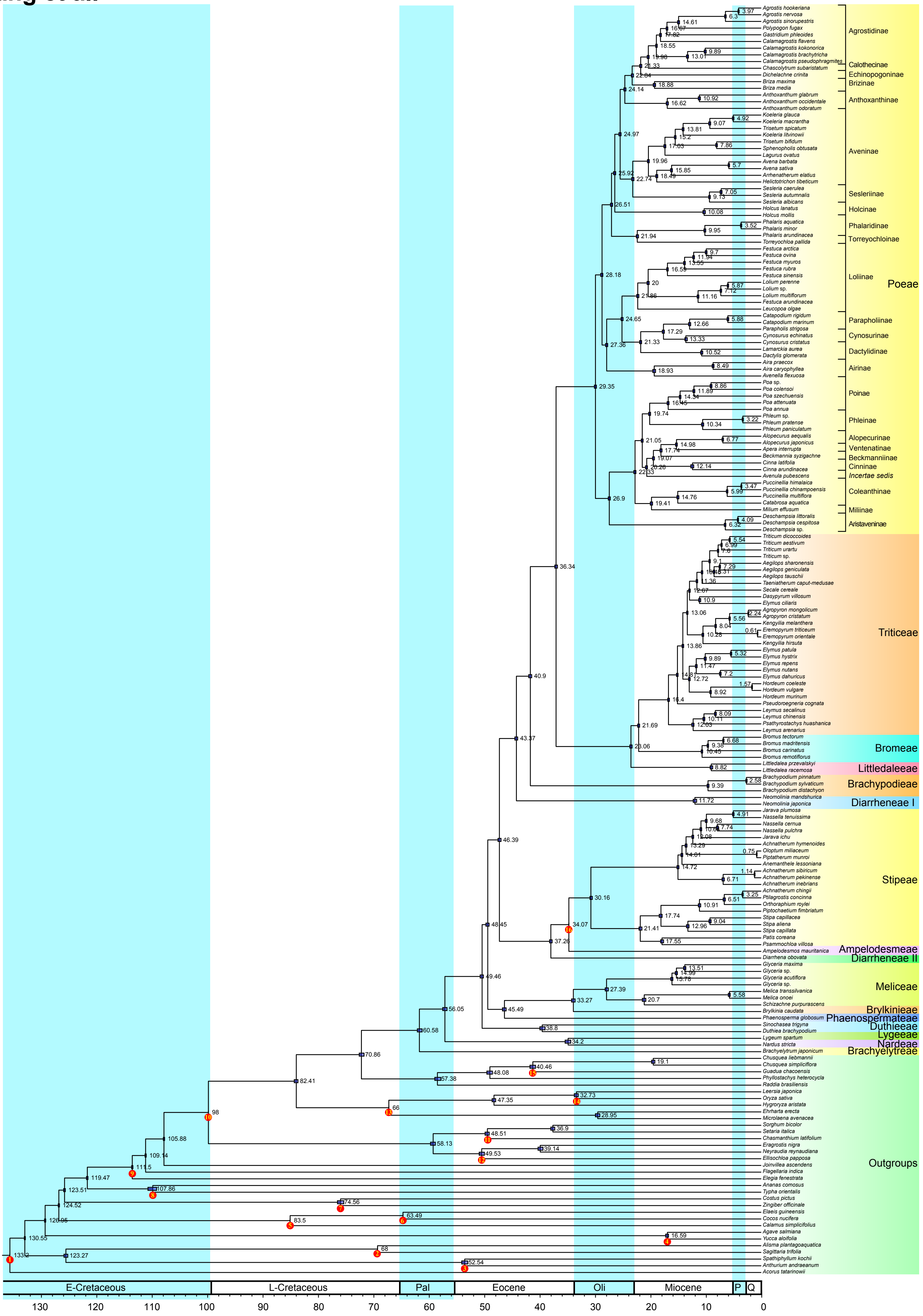

Figure S14 Divergence times of Pooideae inferred by treePL with calibration 2. The red circles with numbers indicate the fossil calibrations corresponding to those in supplementary table S4. Calibration 2 indicates the strategy of inclusion of fossil N13 assigned to stem group of Oryzeae.

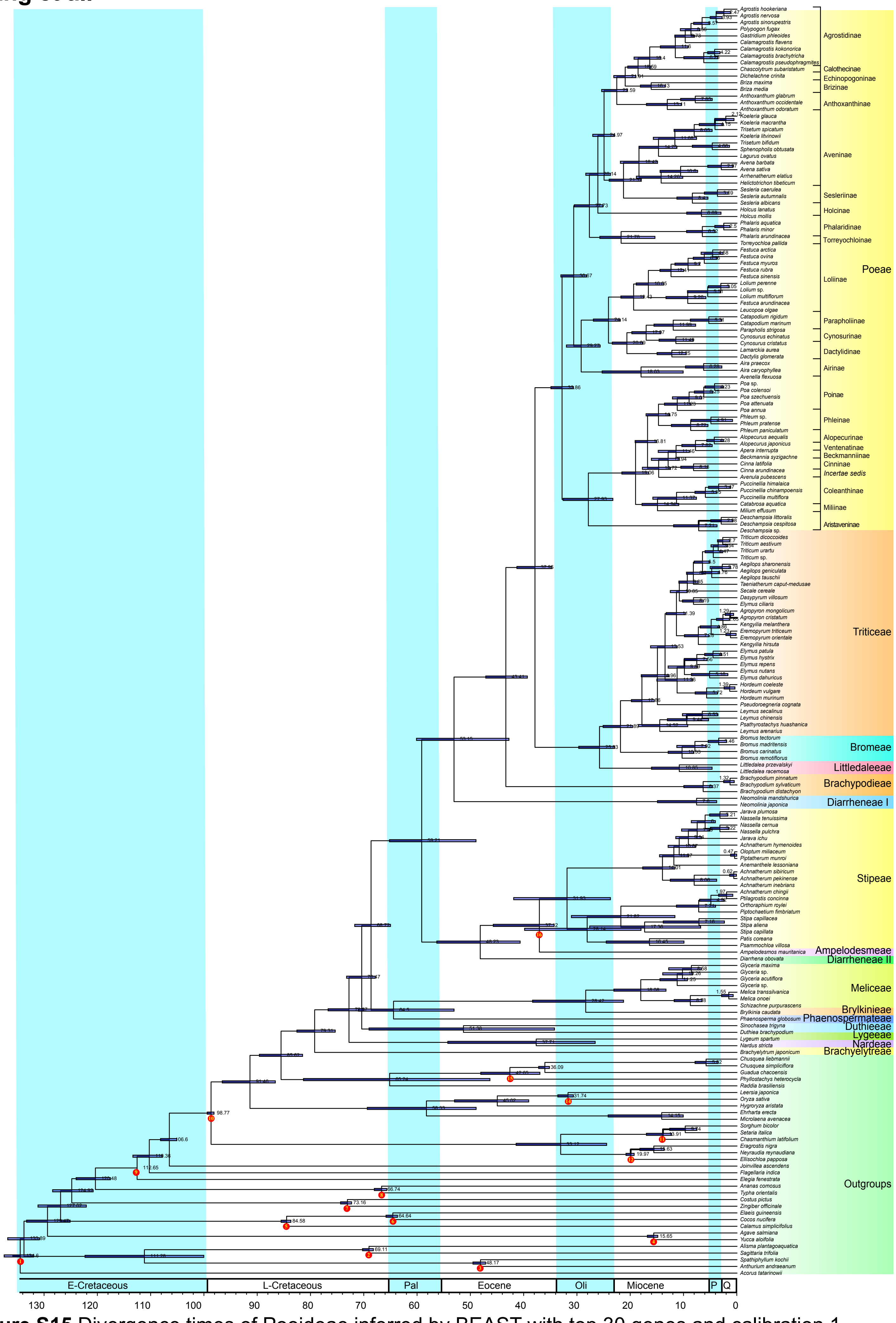

Figure S15 Divergence times of Pooideae inferred by BEAST with top 30 genes and calibration 1. The red circles with numbers indicate the fossil calibration corresponding to those in supplementary table S4. Information of the top 30 genes suggested by clocklikeness is shown in supplementary table S6.

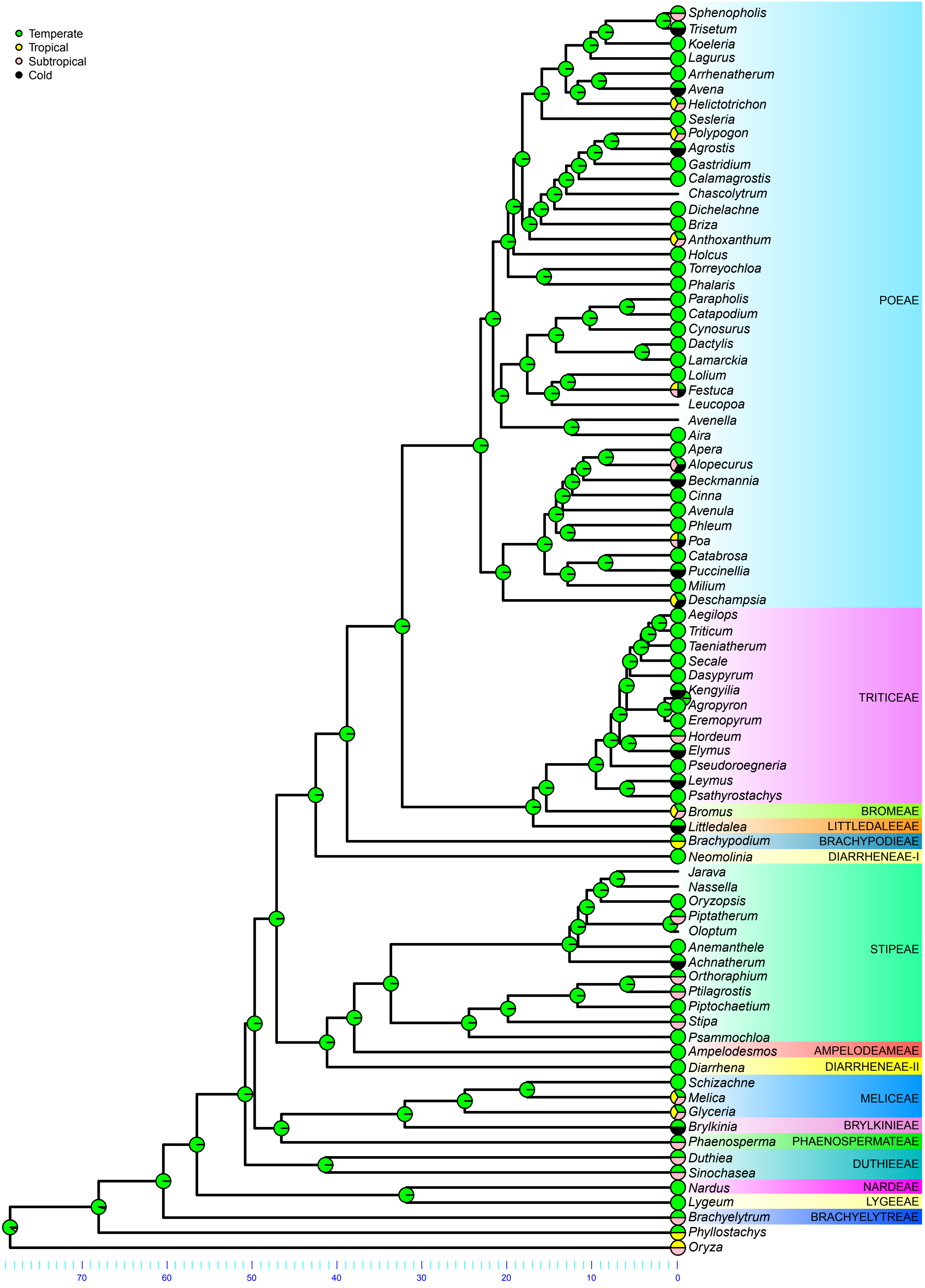

Figure S16 Ancestral character states of climate distribution by analysis using corHMM.
A simplified time tree (supplementary fig. S13) at the genus level was used to trace the ancestral states by corHMM. The colors in pie charts representing the character states are shown in the top-left. Genera (right to the tips) of the same tribe (far right; uppercase) are marked with a colored-background.

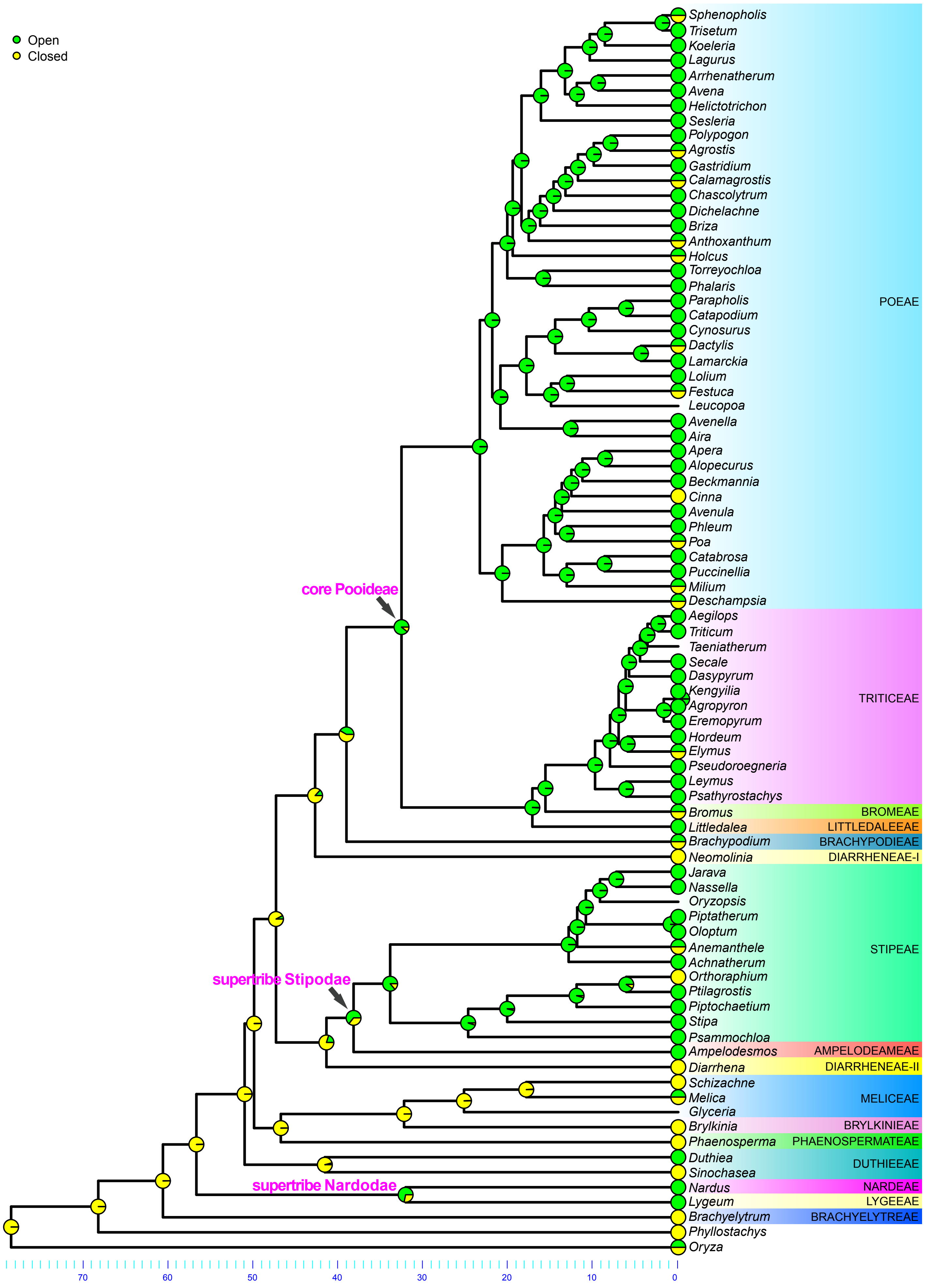

Figure S17 Ancestral character states of habitat by analysis using corHMM.
A simplified time tree (supplementary fig. S13) at the genus level was used to trace the ancestral states by corHMM. The colors in pie charts representing the character states are shown in the top-left. Genera (right to the tips) of the same tribe (far right; uppercase) are marked with a colored-background.

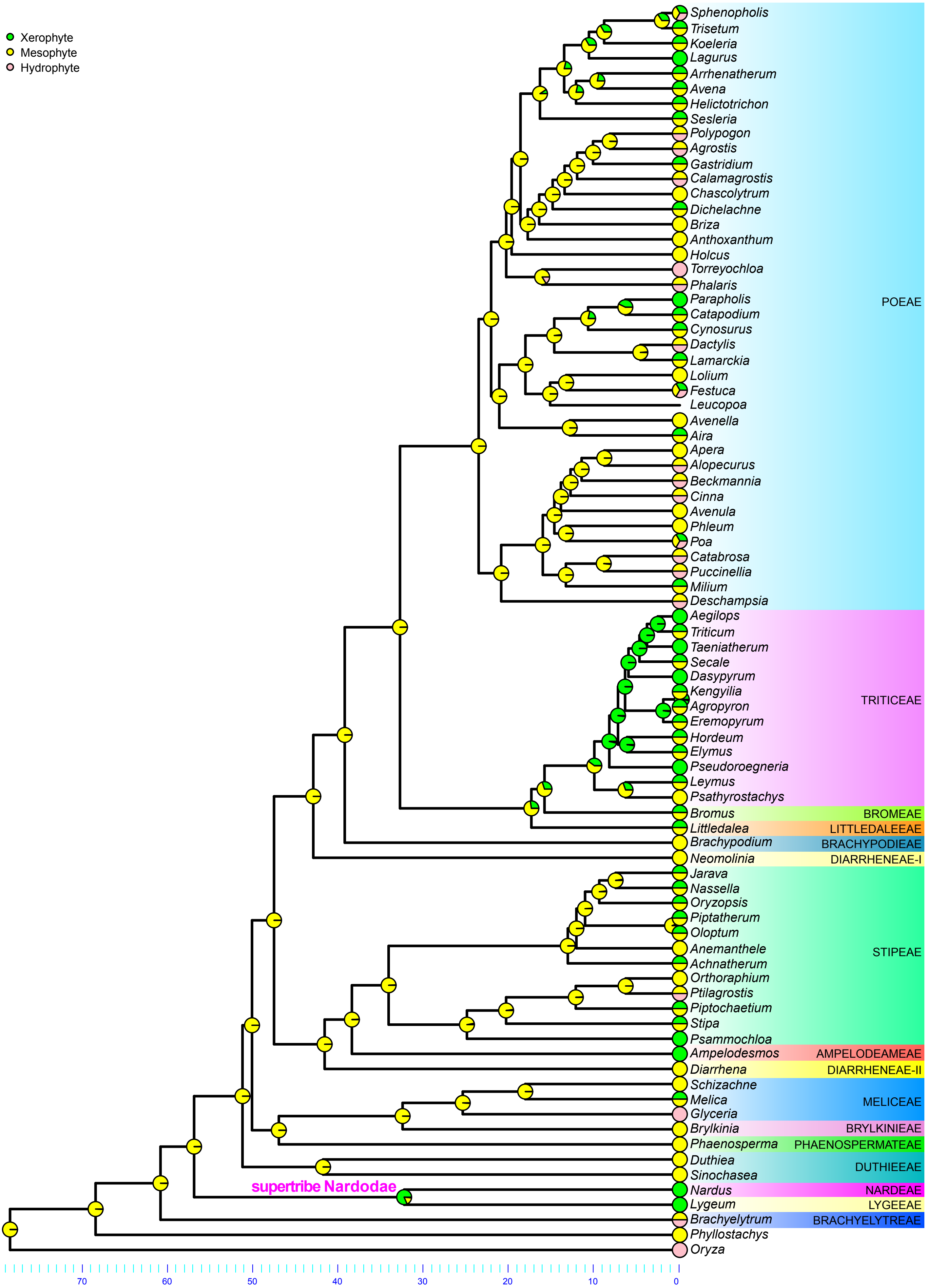

Figure S18 Ancestral character states of adaptation to water availability by analysis using corHMM. A simplified time tree (supplementary fig. S13) at the genus level was used to trace the ancestral states by corHMM. The colors in pie charts representing the character states are shown in the top-left. Genera (right to the tips) of the same tribe (far right; uppercase) are marked with a colored-background.

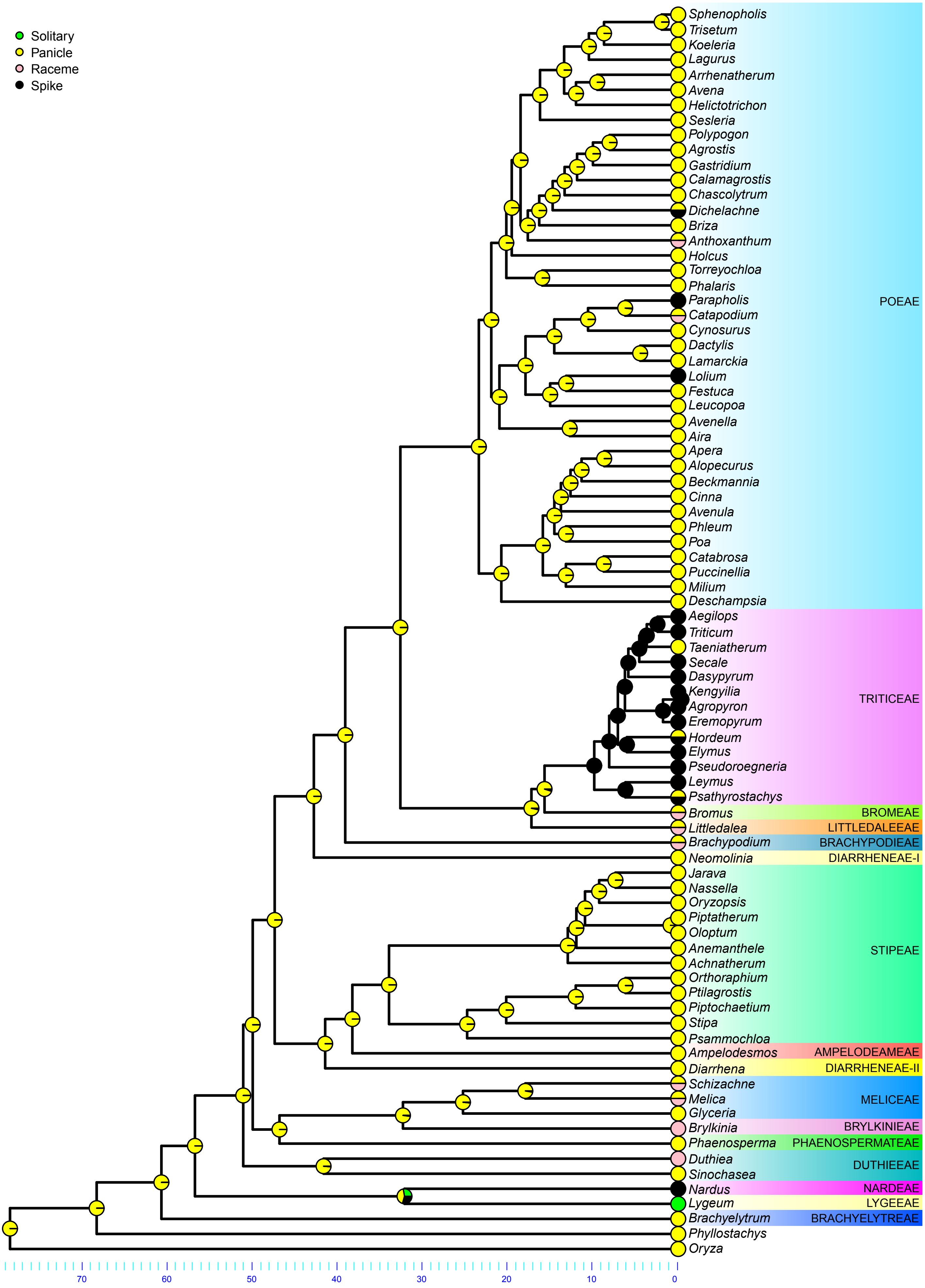

Figure S19 Ancestral character states of inflorescence by analysis using corHMM.
A simplified time tree (supplementary fig. S13) at the genus level was used to trace the ancestral states by corHMM. The colors in pie charts representing the character states are shown in the top-left. Genera (right to the tips) of the same tribe (far right; uppercase) are marked with a colored-background.

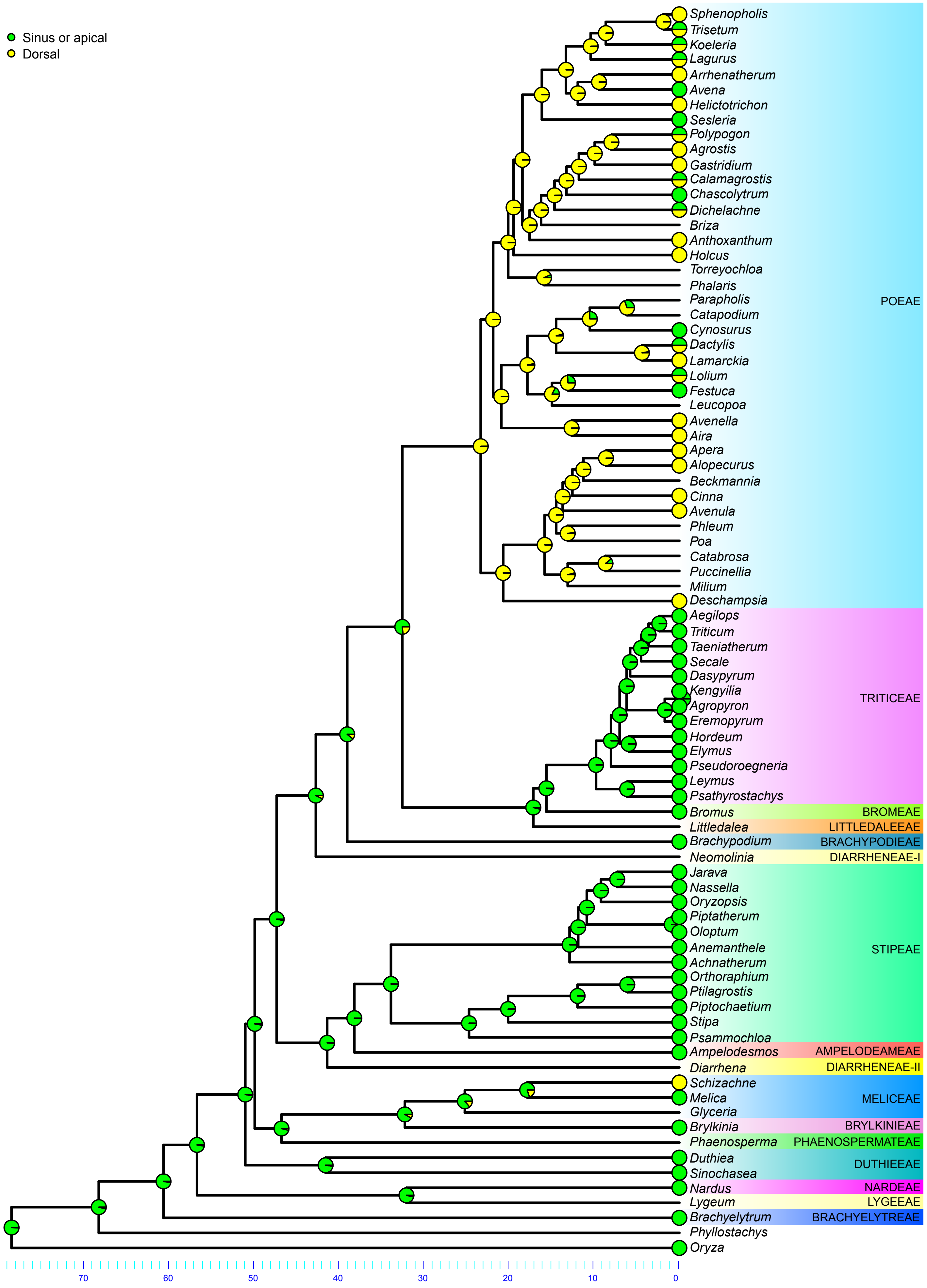

Figure S20 Ancestral character states of awn location by analysis using corHMM.
A simplified time tree (supplementary fig. S13) at the genus level was used to trace the ancestral states by corHMM. The colors in pie charts representing the character states are shown in the top-left. Genera (right to the tips) of the same tribe (far right; uppercase) are marked with a colored-background.

Zhang et al.

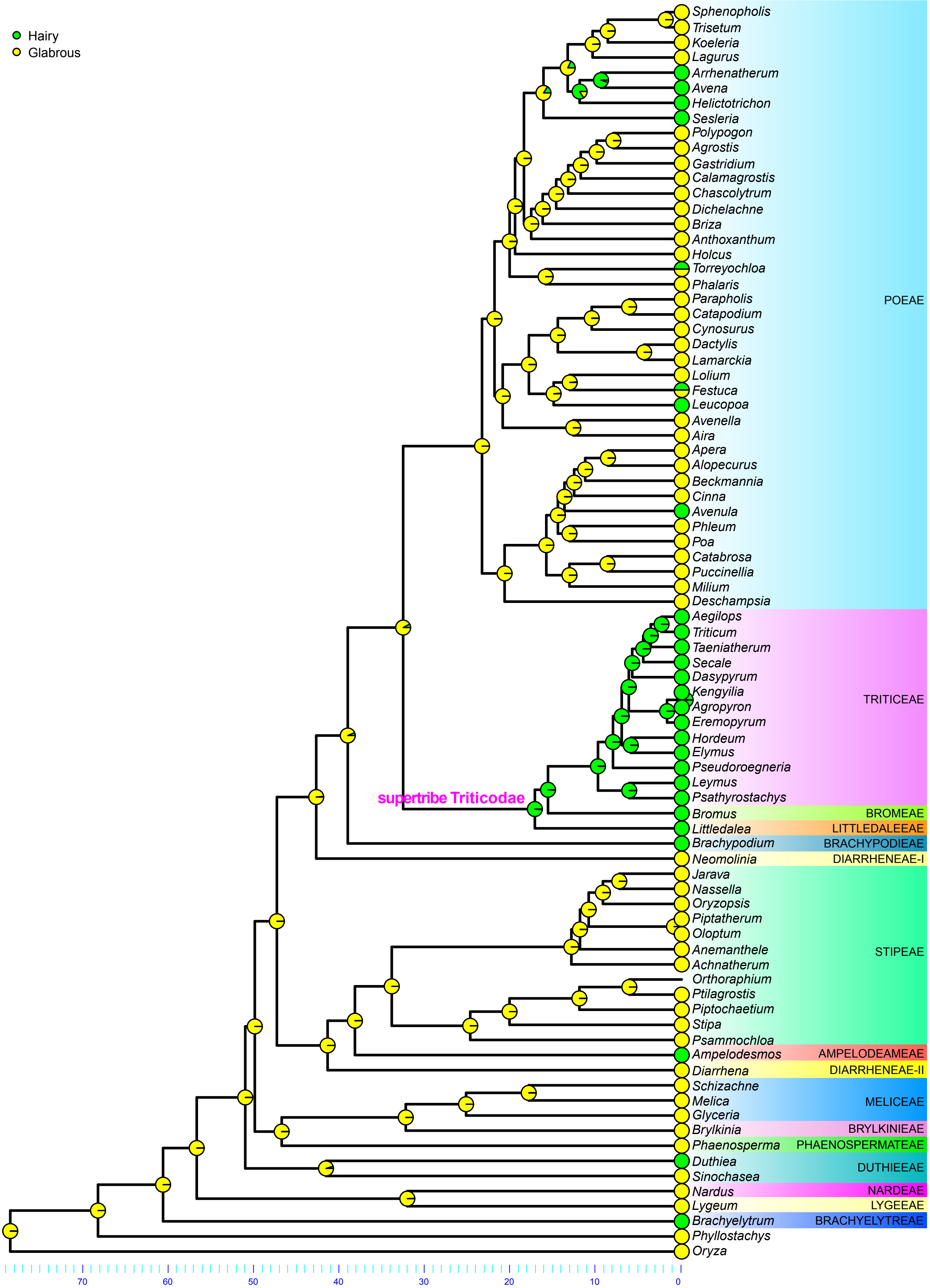

Figure S21 Ancestral character states of ovary indumentum by analysis using corHMM. A simplified time tree (supplementary fig. S13) at the genus level was used to trace the ancestral states by corHMM. The colors in pie charts representing the character states are shown in the top-left. Genera (right to the tips) of the same tribe (far right; uppercase) are marked with a colored-background.

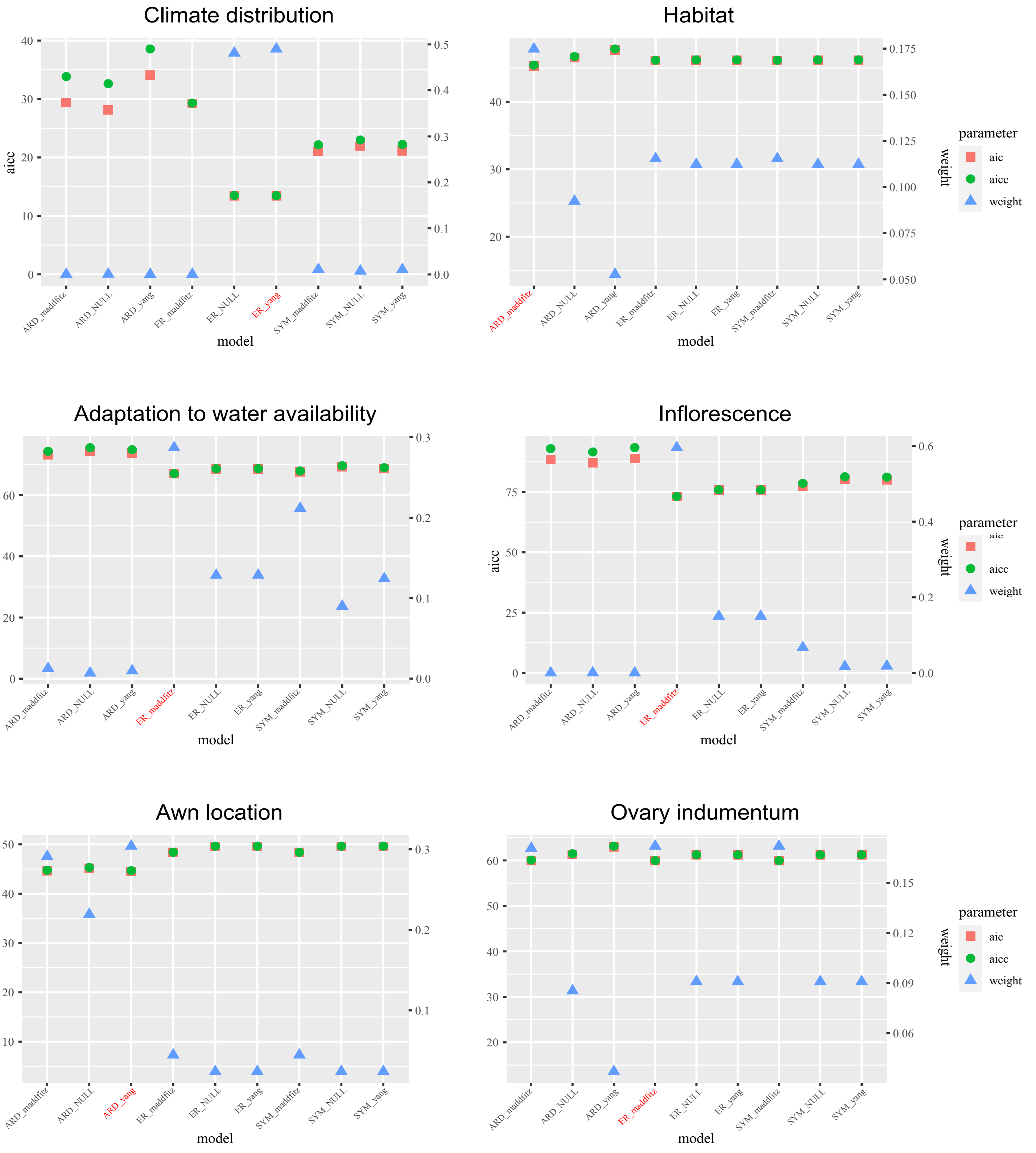

Figure S22 Parameters of ancestral character states inferred by using corHMM with different models. Different shapes in each panel represent different parameters are shown on the right. The X-axis shows models and the y-axis shows the aic(c) value (left) and the weight value (right). The model with red is the one chosen in this study.

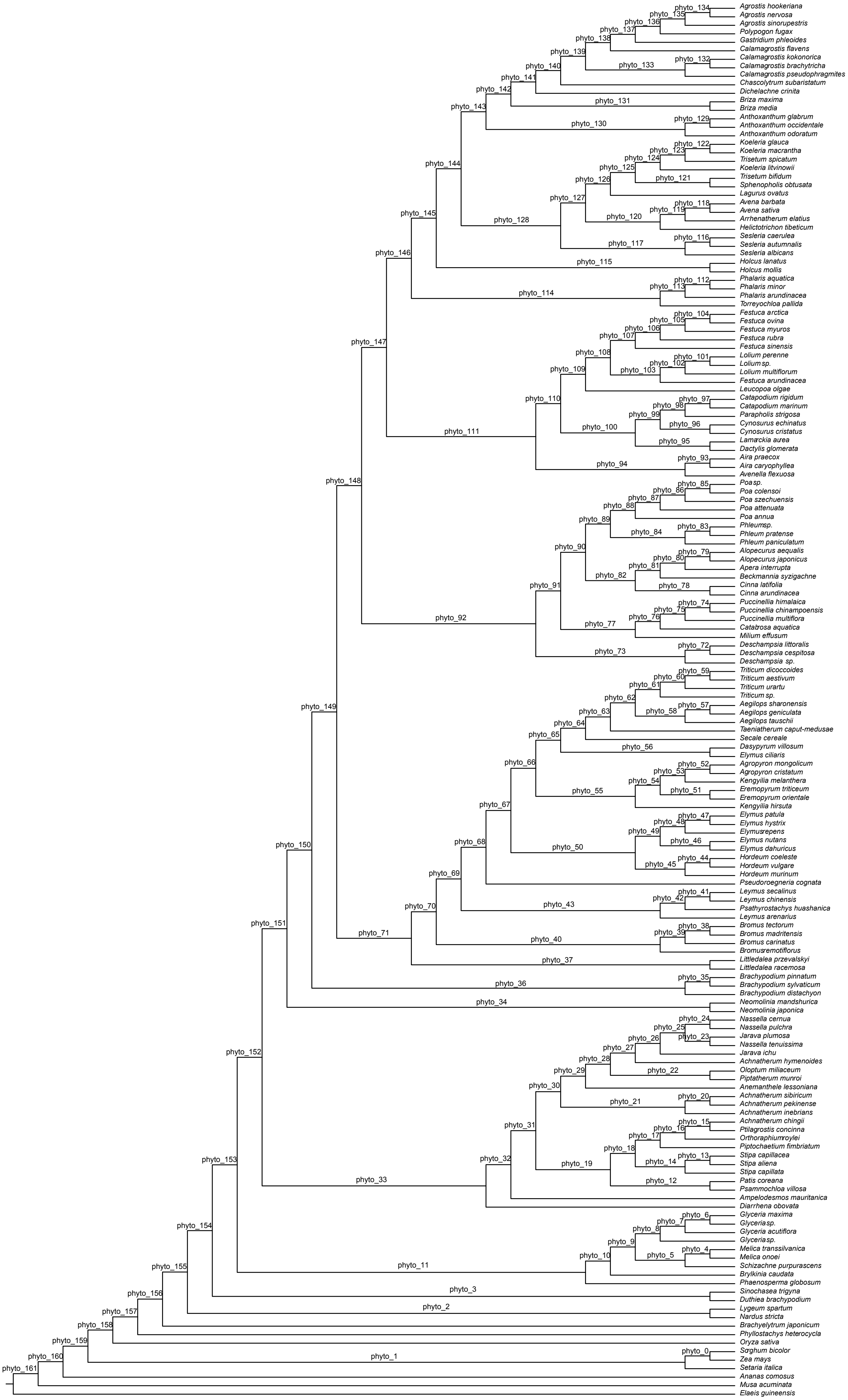

Figure S23 A species tree with the identity of nodes used in tree reconciliation.

Zhang et al.

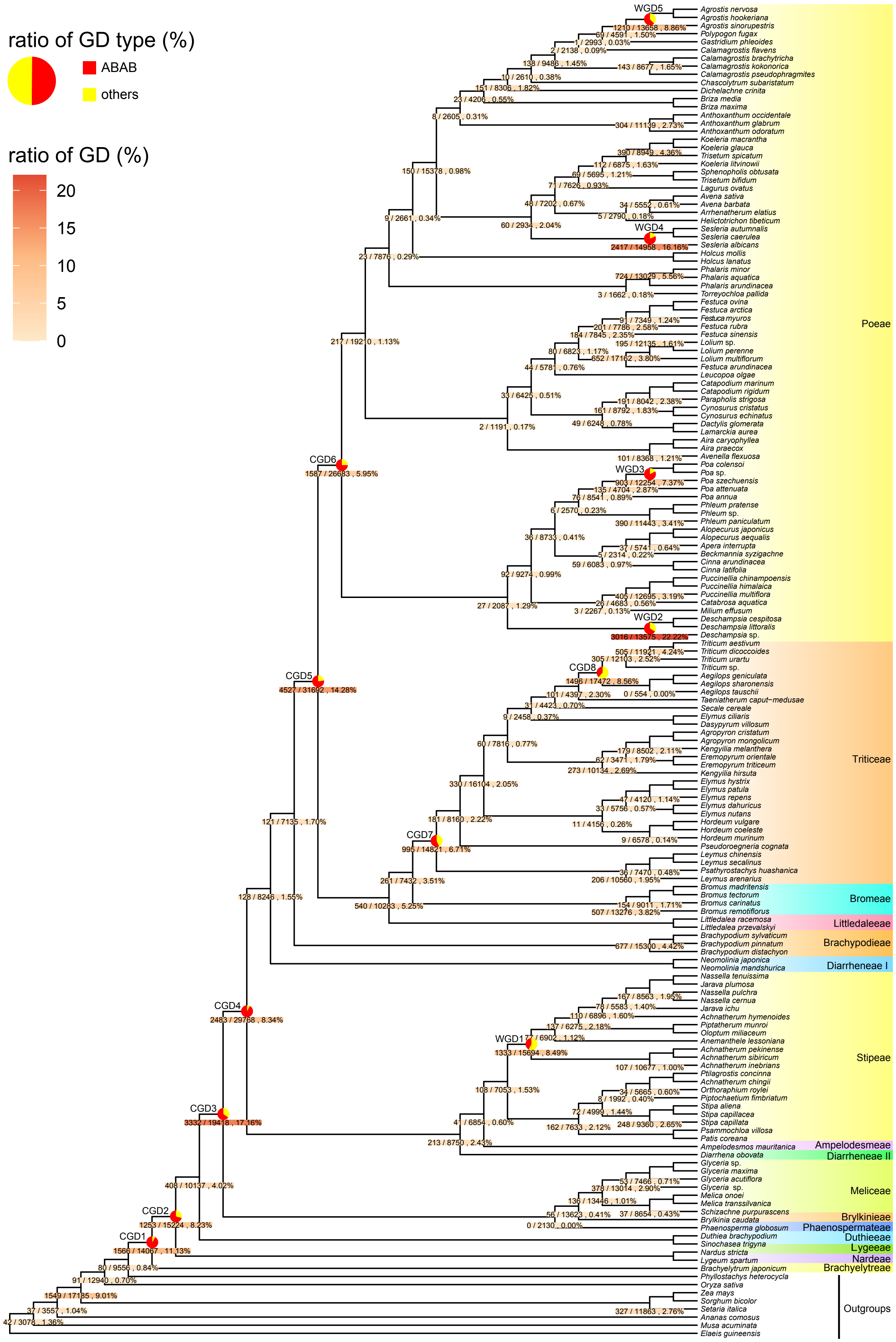

Figure S24 Information of gene duplications (GD) in each node.
The numbers of GD, GF and ratio (GD out GF) are shown in rectangle with red-gradient (corresponding to the GD ratio legend) beside each node. The nodes of 13 GD clusters are marked with pie and indicated the proportion of GD types (corresponding to the GD type legend with red and yellow). Species belonging to the same tribe are indicated by the same background color with the tribe name shown on the right.

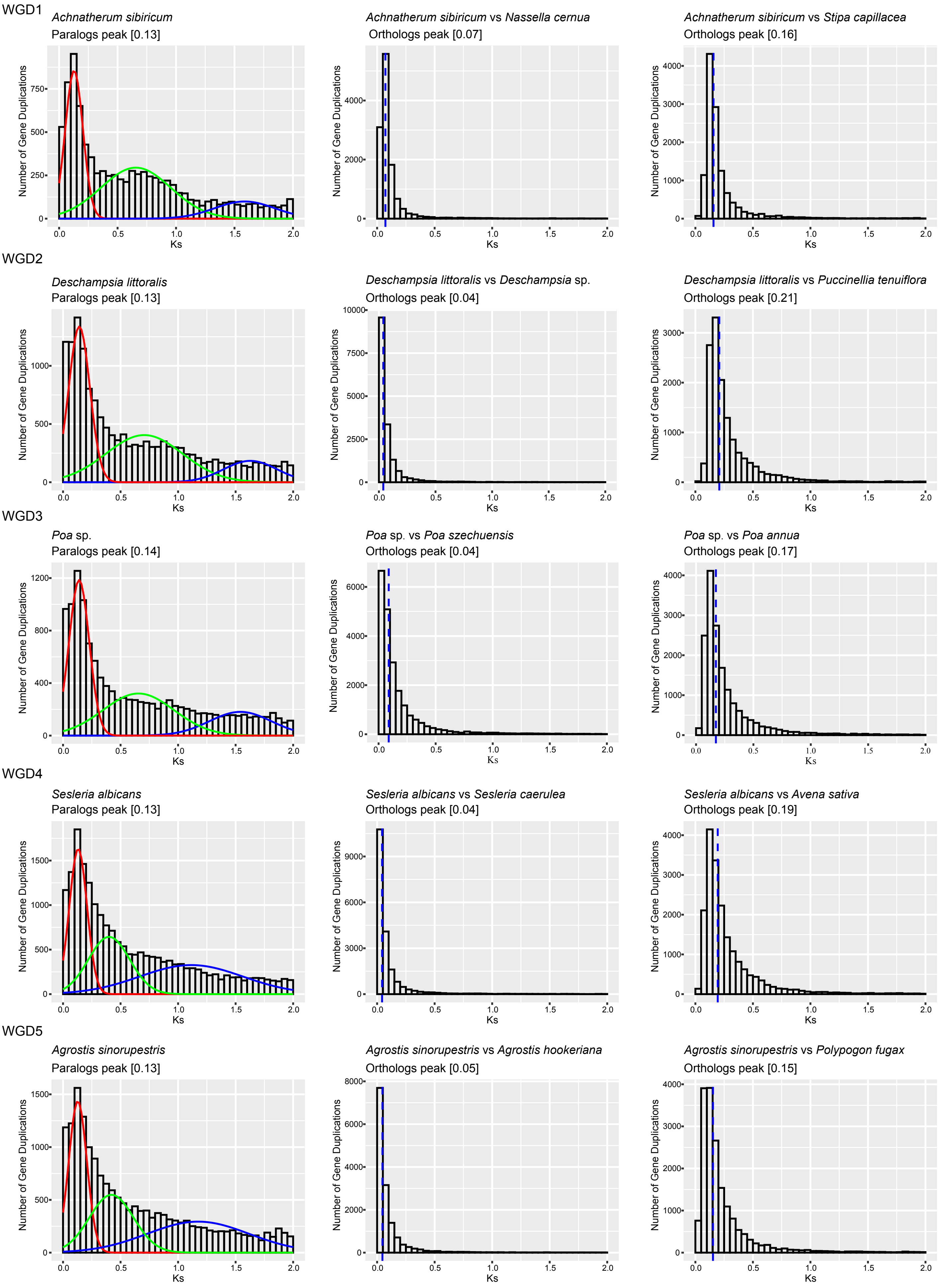

Figure S25 Estimation of Ks peak of WGDs (paralogs) and speciation (orthologs) before and after each WGD.
Histograms of the age distribution of gene duplications (Ks plots) with a binwidth of 0.05. The numbers within brackets represent the median Ks values of the paralogs or orthologs.

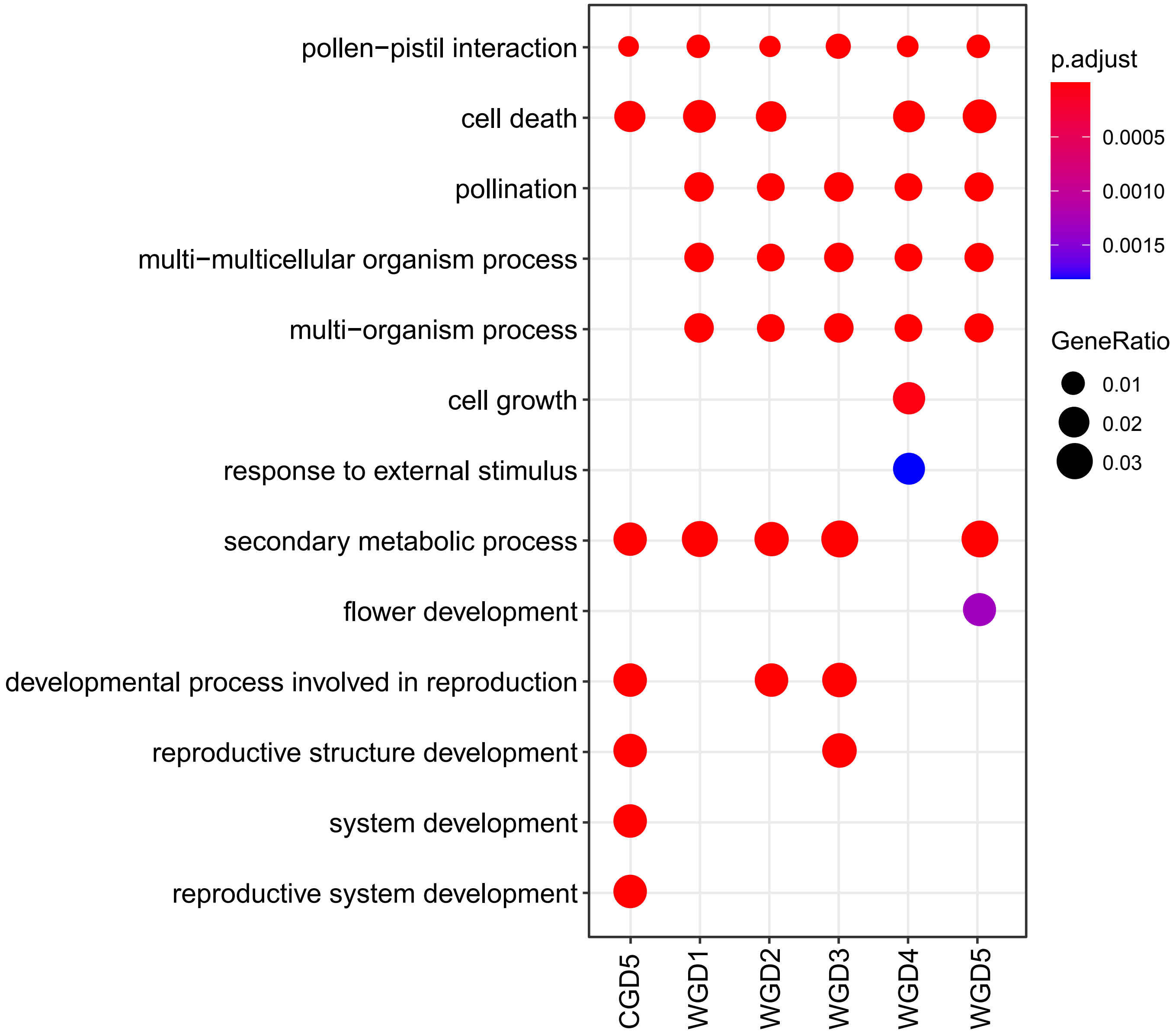

Figure S26 GO enrichment of duplicates derived from WGDs at GO level 4, using the *Oryza sativa* genome as a reference.

Sizes of the circles represent the proportion of genes belonging to the indicated category in all gene duplicates derived from the same cluster of gene duplications, and the color shows the significance level. The functional categories are from the slim version of GO categories.

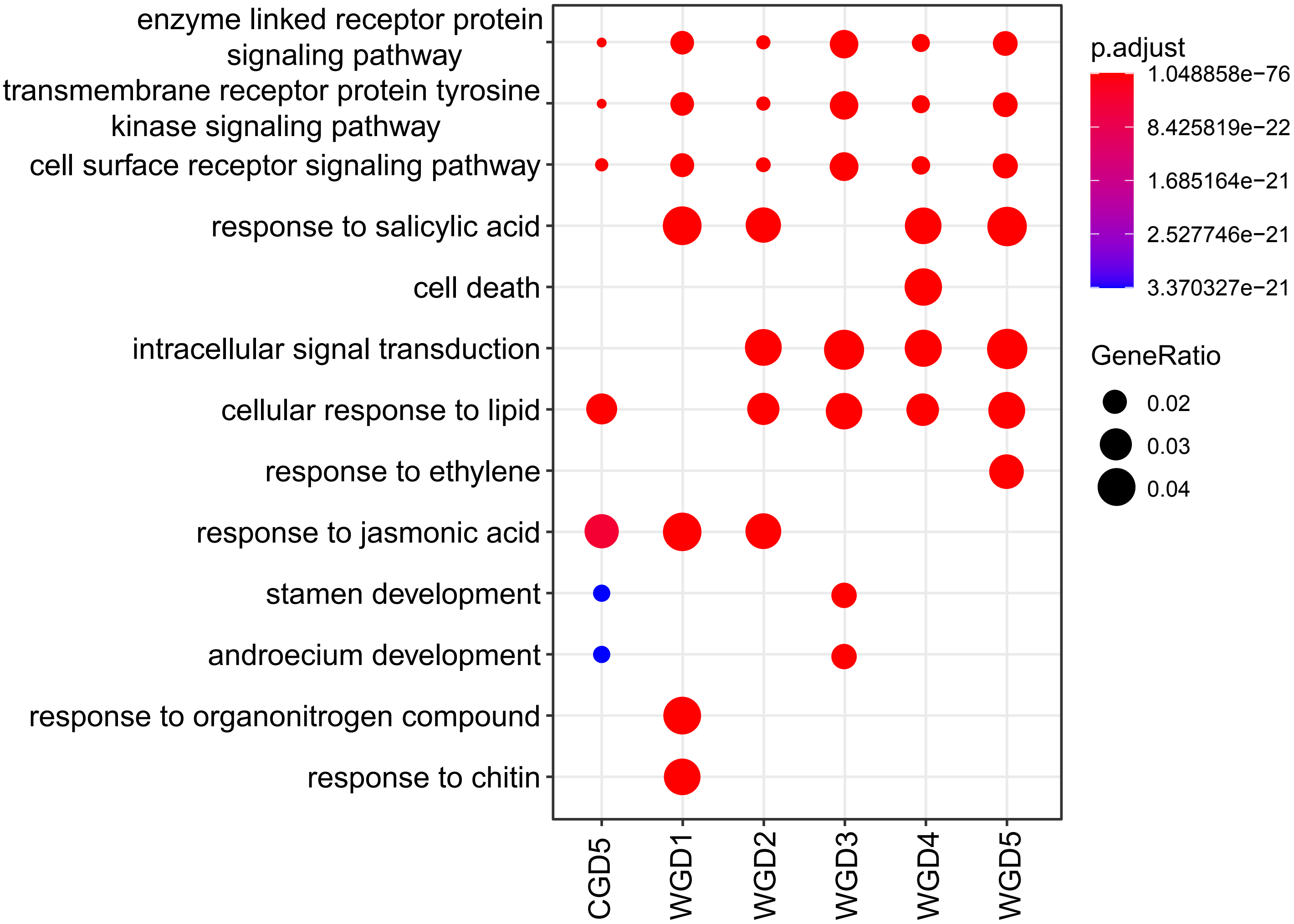

Figure S27 GO enrichment of duplicates derived from WGDs at GO level 4, using the *Arabidopsis thaliana* genome as a reference.

Sizes of the circles represent the proportion of genes belonging to the indicated category in all gene duplicates derived from the same cluster of gene duplications, and the color shows the significance level. The functional categories are from the slim version of GO categories.

Zhang et al.

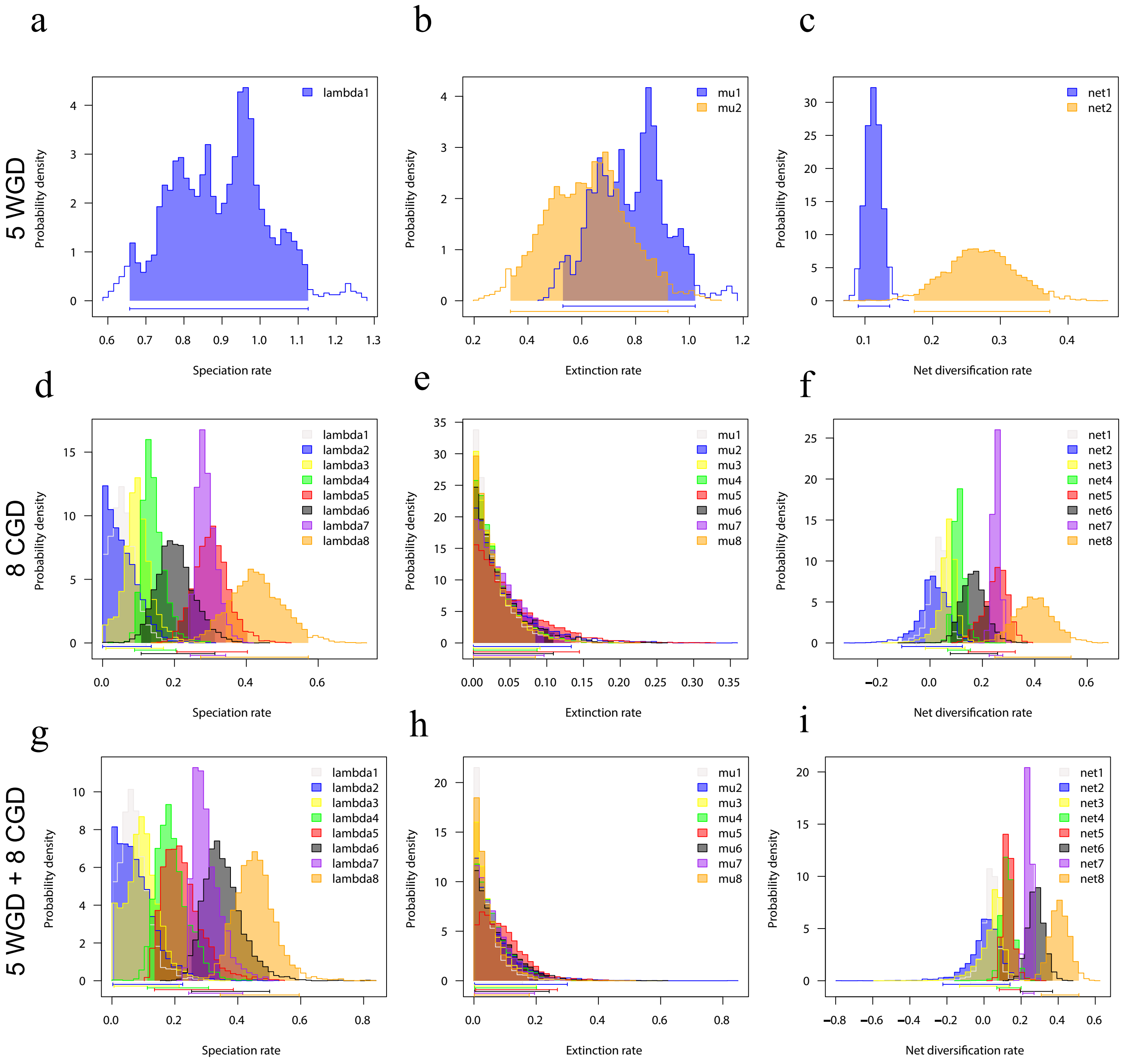

Figure S28 CGD/WGD-related diversification rates.
The state-dependent posterior distribution of speciation (lambda), extinction (mu) and net diversification rates (speciation minus extinction) were from MuSSE with 5,000 MCMC generations under the best-fitting models according to the AICc criterion. There are two character states for the five WGDs, eight for the eight CGDs and eight for the sum of WGDs and CGDs. Only the speciation rate of the WGDs is fixed in the best model. Lambda1 and mu1 are rates with 0 GD, lambda 2 and mu 2 are with 1 GD, and so on.

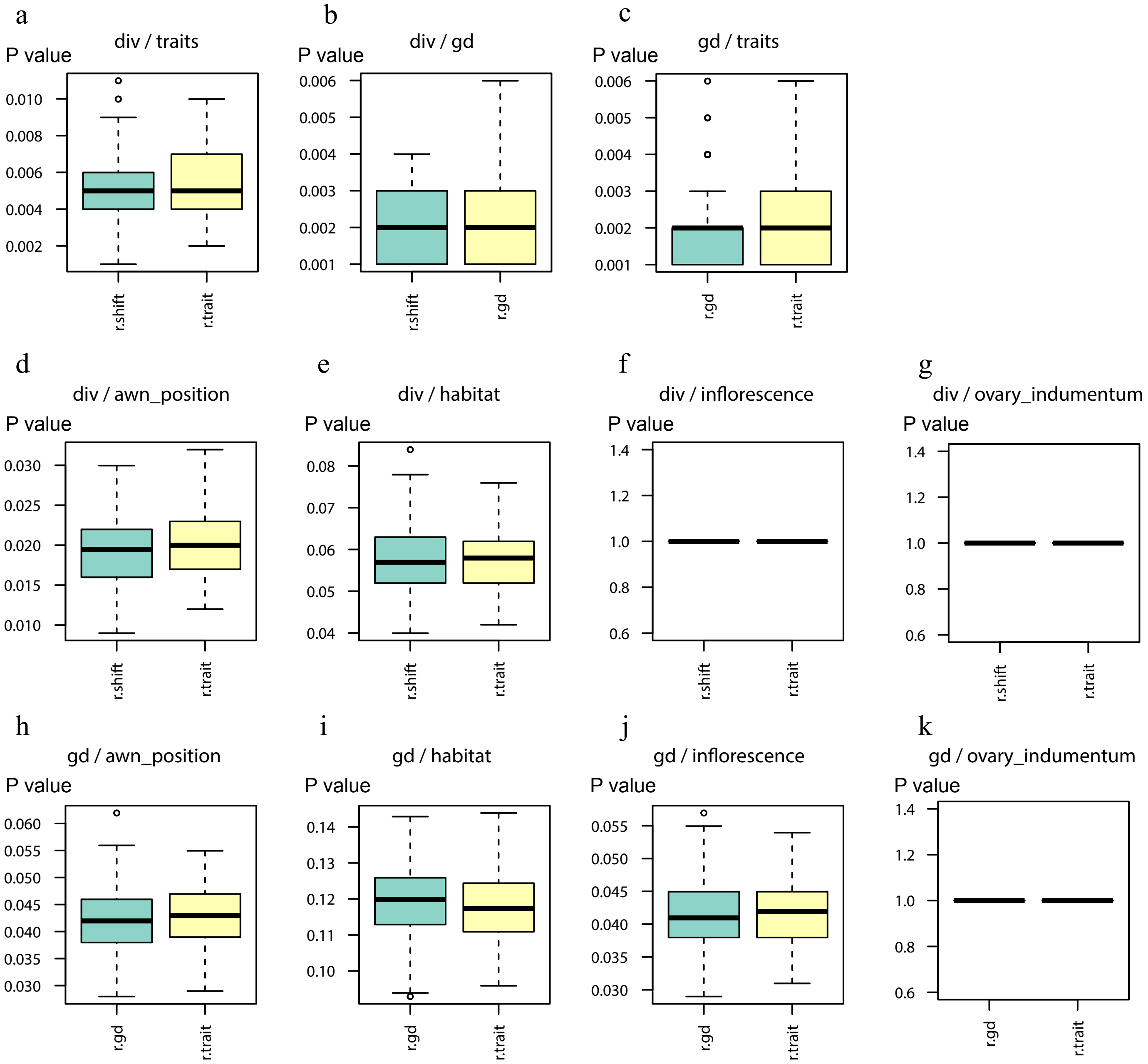

Figure S29 Statistical test for the correlation of GDs, diversification upshifts and transitions of traits. The two features in each analysis are noted at top of each panel. Boxplots show the quantiles of p values from 100 repeats (see Methods). r.shift, analysis compared with the null hypothesis using randomly placed nodes of rate shift; r.trait, analysis compared with the null hypothesis using randomly placed nodes of trait transition; r.gd, analysis compared with the null hypothesis using randomly placed GD nodes.

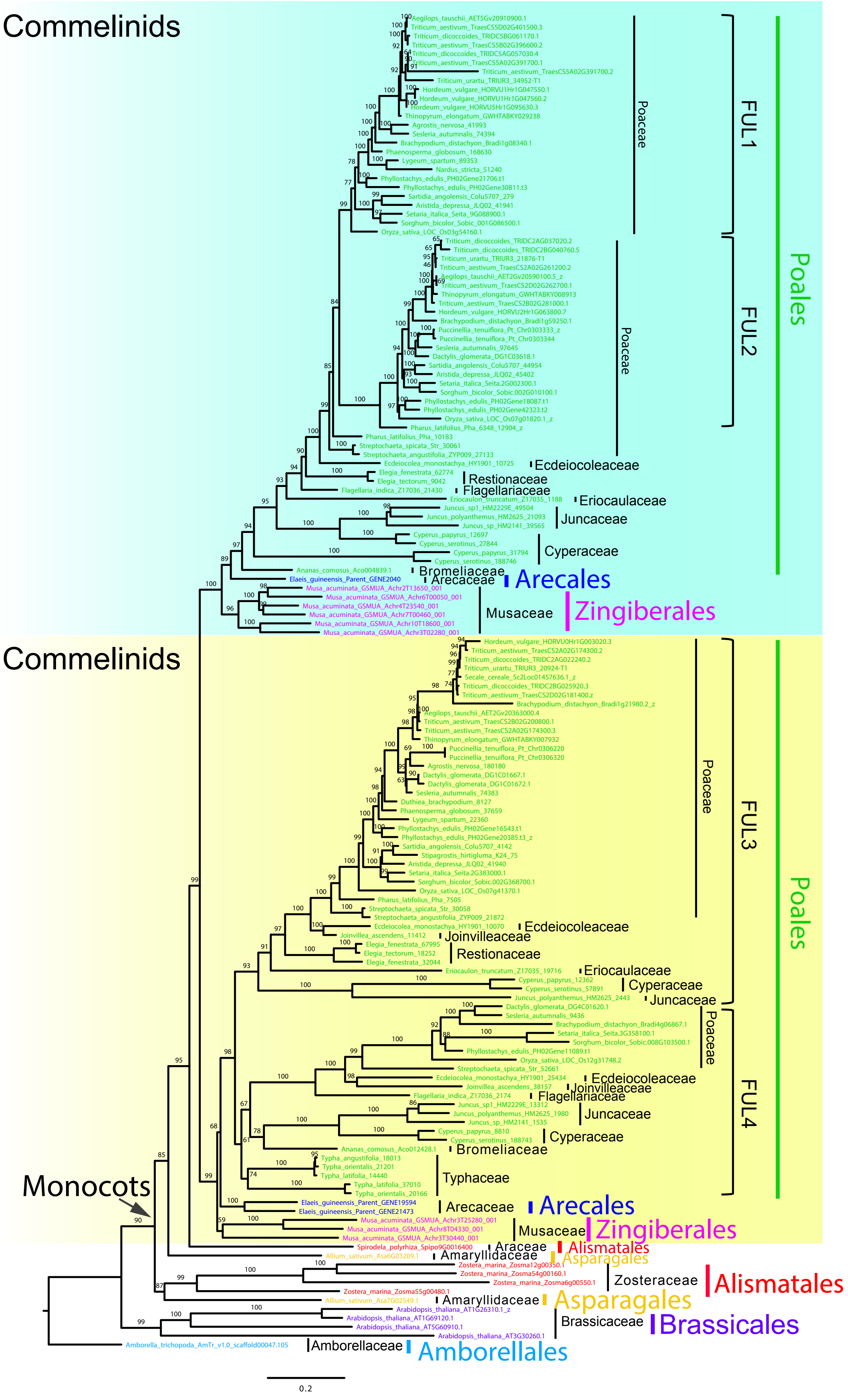

Figure S30 The phylogeny of *AP1/FUL*-like genes inferred by IQ-TREE.
TThe tips are named with species name plus gene id. The same color indicates those species are within the same order (right). Bootstrap values are shown on branches. The background colors (blue and yellow) show the early duplication of *AP1/FUL*-like. The clade of FUL3-FUL4 and FUL1-FUL2 show the other two duplications corresponding to fig. 5 (circles 1-3).

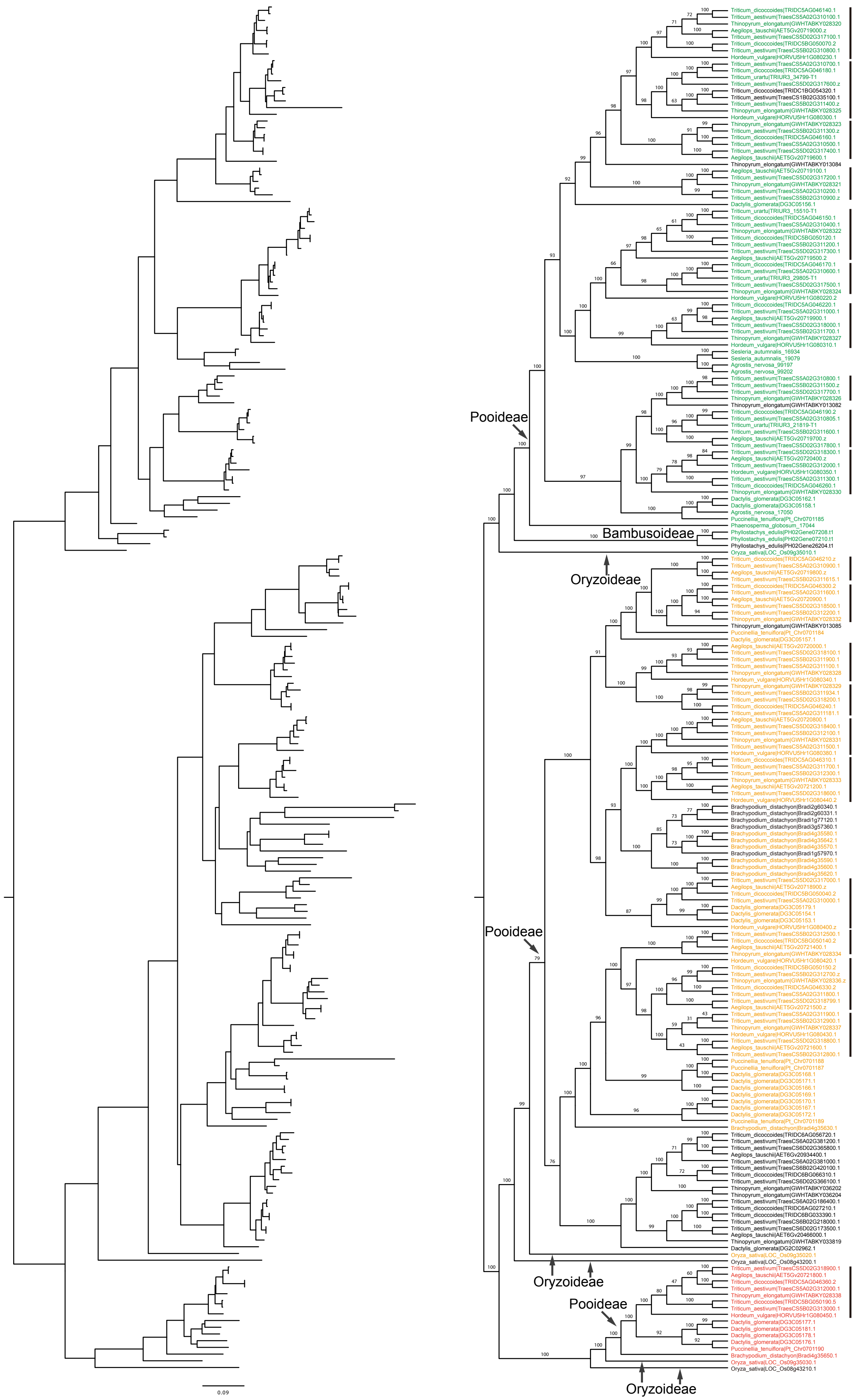

Figure S31 The phylogeny of *CBF* genes inferred by IQ-TREE.
The phylogram (left) and cladogram (right) of *CBF* tree are presented. The species demonstrated in green, yellow and red are correspond to the same background color of the tree in fig. 6a and the same colored box in fig. 6b. The species in black represent those that are not considered in fig. 6. The number 0-20 (corresponding to fig. 6) represents a clade (orthologs group) that derived from the last gene duplication. Bootstrap values are indicated beside the nodes.

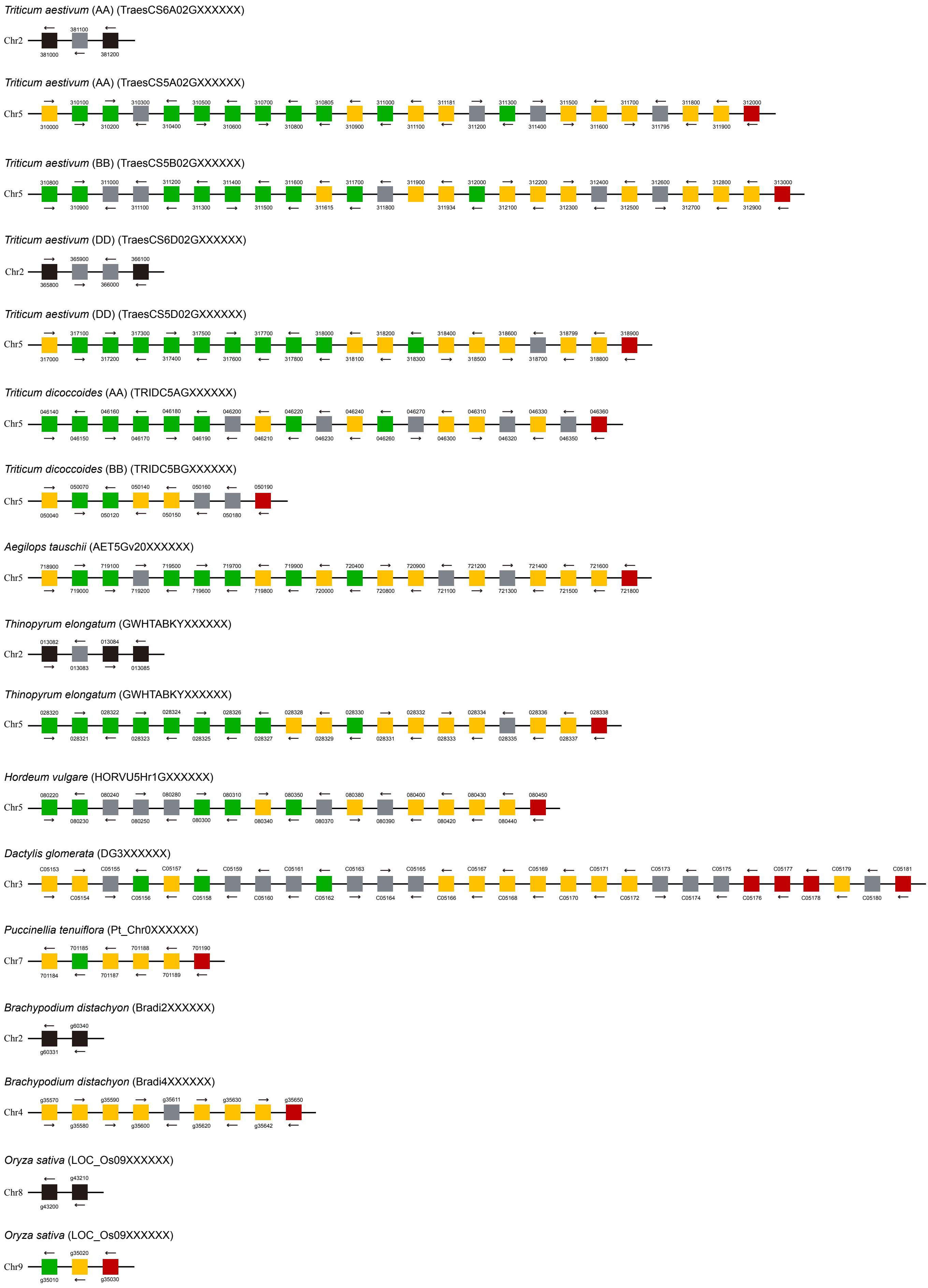

Figure S32 Gene location of *CBF* genes.

The boxes demonstrated in green, yellow and red are corresponded to fig. 6 and supplementary fig. S31. The gray boxes indicate the genes adjacent to CBF genes but not in the tree here. The black boxes indicate tandem genes but not shown in fig. 6. The arrows close to the boxs represent the gene directions of transcription.

a

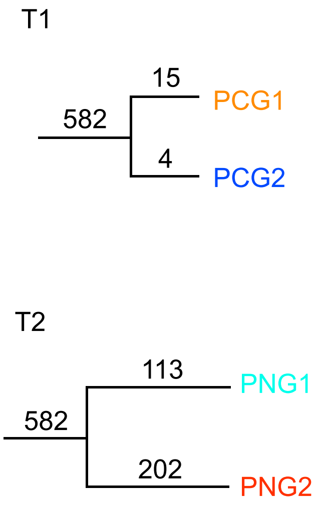

b

This study	Previous study
Agrostidinae	Agrostidinae
Calothecinae	Calothecinae
Echinopogoninae	Echinopogoninae
Brizinae	Brizinae
Aveninae	Aveninae
Anthoxanthinae	Anthoxanthinae
Phalaridinae	Phalaridinae
Torreyochloinae	Torreyochloinae
Holcinae	Holcinae
Sesleriinae	Sesleriinae
Parapholiinae	Parapholiinae
Cynosurinae	Cynosurinae
Dactylidinae	Dactylidinae
Loliinae	Loliinae
Airinae	Airinae
Aristaveninae	Aristaveninae
Ventenatinae	Ventenatinae
Alopecurinae	Alopecurinae
Cinninae	Cinninae
Beckmanniinae	Beckmanniinae
Phleinae	Phleinae
Poinae	Poinae
Miliinae	Miliinae
Coleanthinae	Coleanthinae

Figure S33 Concordance of 1234 trees with different topologies inferred by Phypart.
(a) Single-gene trees of the 1234 OGs were compared with the topologies proposed by previous (T1) and this study (T2). When a node of a gene tree covering the same group of species as any node on the compared topology, a concordance is counted on the node of the compared topology. We also required the bootstrap value of the node on the gene tree to be higher than 70. PCG1: Poeae chloroplast group 1; PCG2: Poeae chloroplast group 2. PNG1: Poeae nuclear group 1; PNG2: Poeae nuclear group 2. (b) Detailed topologies of clades (tips) are shown in (a).

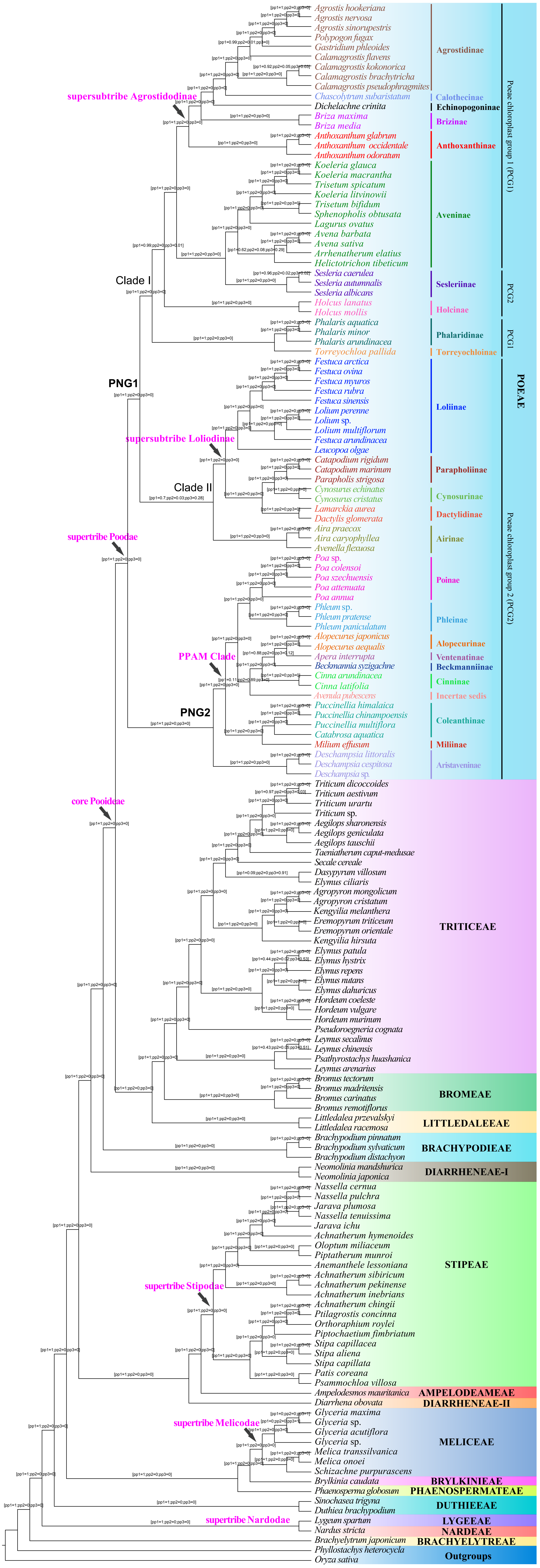

Figure S34 Branch annotation of local posterior probabilities inferred from 1234 OGs by ASTRAL. Local posterior probabilities are indicated in the following order: the main topology (pp1), one for each of the two alternatives (pp2 and pp3), with their sum being 1. Tribes, subtribes and major lineages are the same as indicated in supplementary figs. S5-S9.

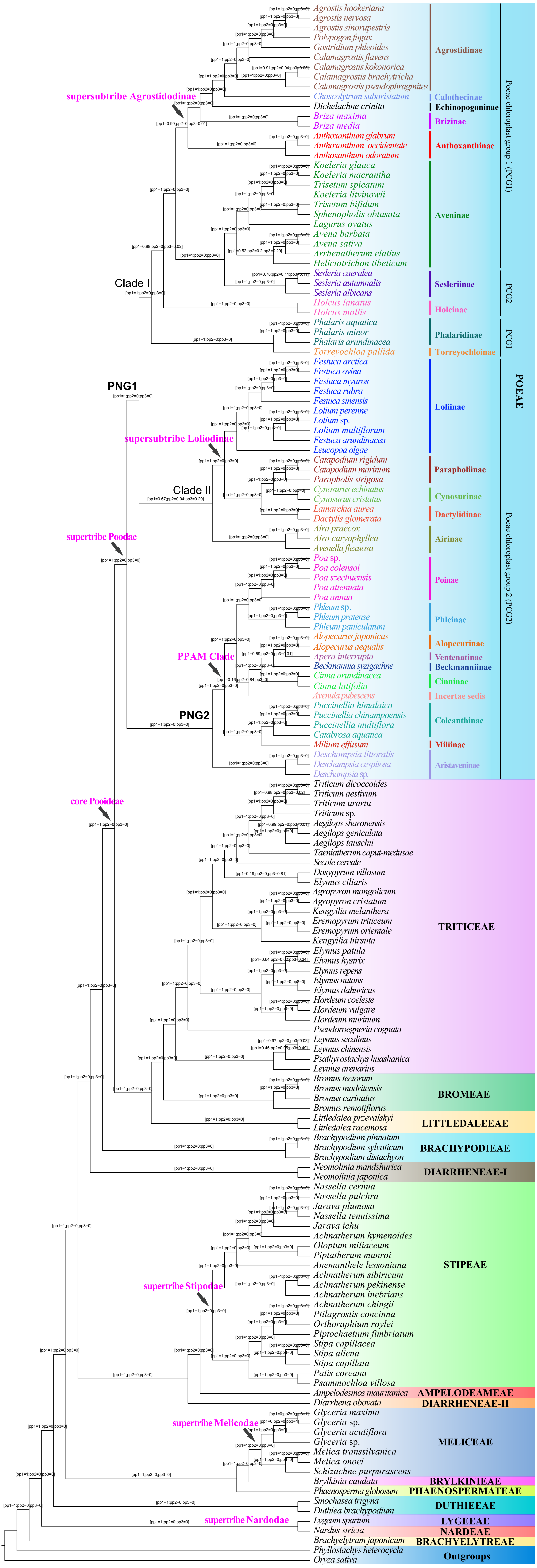

Figure S35 Branch annotation of local posterior probabilities inferred from 914 OGs by ASTRAL. Local posterior probabilities are indicated in the following order: the main topology (pp1), one for each of the two alternatives (pp2 and pp3), with their sum being 1. Tribes, subtribes and major lineages are the same as indicated in supplementary figs. S5-S9.

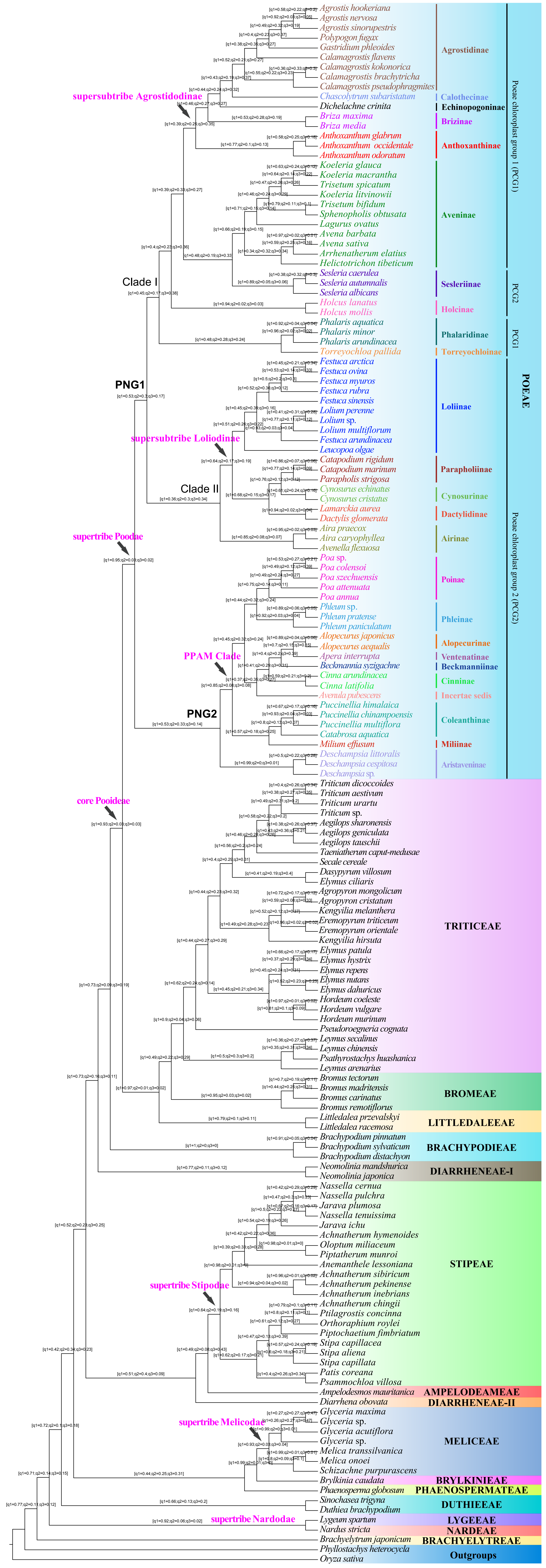

Figure S36 Branch annotation of local posterior probabilities inferred from 763 OGs by ASTRAL.
Local posterior probabilities are indicated in the following order: the main topology (pp1), one for each of the two alternatives (pp2 and pp3), with their sum being 1. Tribes, subtribes and major lineages are the same as indicated in supplementary figs. S5-S9.

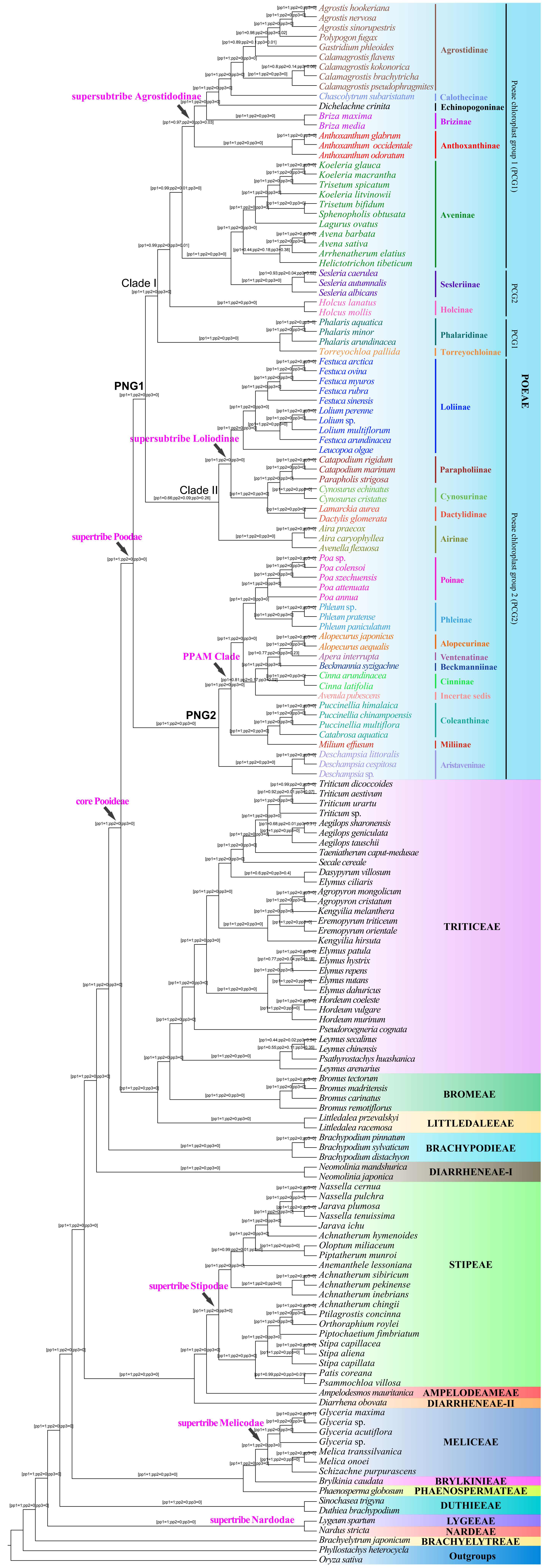

Figure S37 Branch annotation of local posterior probabilities inferred from 512 OGs by ASTRAL. Local posterior probabilities are indicated in the following order: the main topology (pp1), one for each of the two alternatives (pp2 and pp3), with their sum being 1. Tribes, subtribes and major lineages are the same as indicated in supplementary figs. S5-S9.

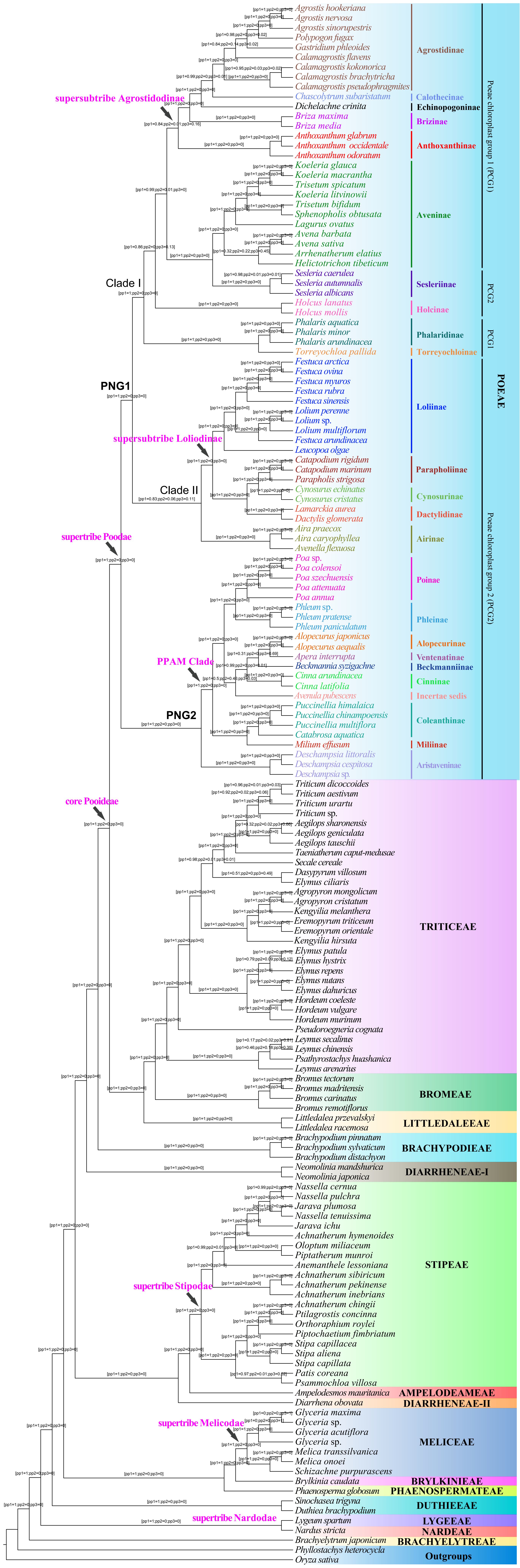

Figure S38 Branch annotation of local posterior probabilities inferred from 373 OGs by ASTRAL. Local posterior probabilities are indicated in the following order: the main topology (pp1), one for each of the two alternatives (pp2 and pp3), with their sum being 1. Tribes, subtribes and major lineages are the same as indicated in supplementary figs. S5-S9.

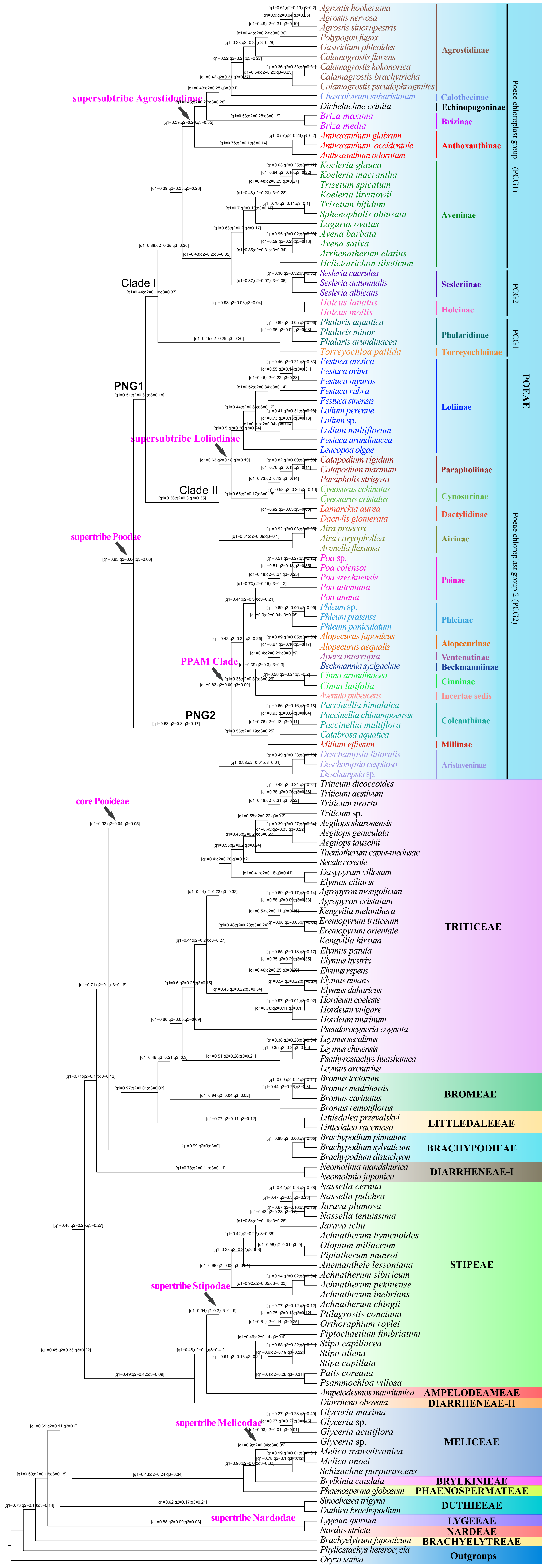

Figure S39 Branch annotation of quartet support values inferred from 1234 OGs by ASTRAL.
Quartet support values are indicated in the following order: the main topology (q1), one for each of the two alternatives (q2 and q3). Tribes, subtribes and major lineages are the same as supplementary figs. S5-S9.

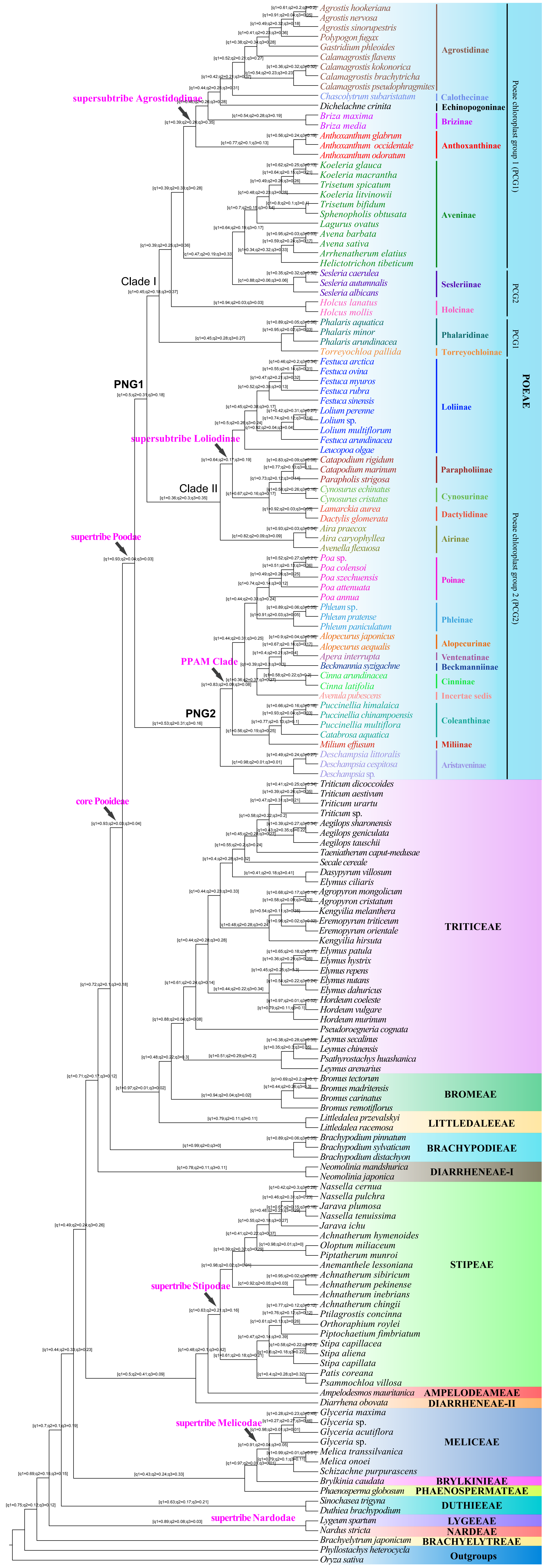

Figure S40 Branch annotation of quartet support values inferred from 914 OGs by ASTRAL.
Quartet support values are indicated in the following order: the main topology (q1), one for each of the two alternatives (q2 and q3). Tribes, subtribes and major lineages are the same as supplementary figs. S5-S9.

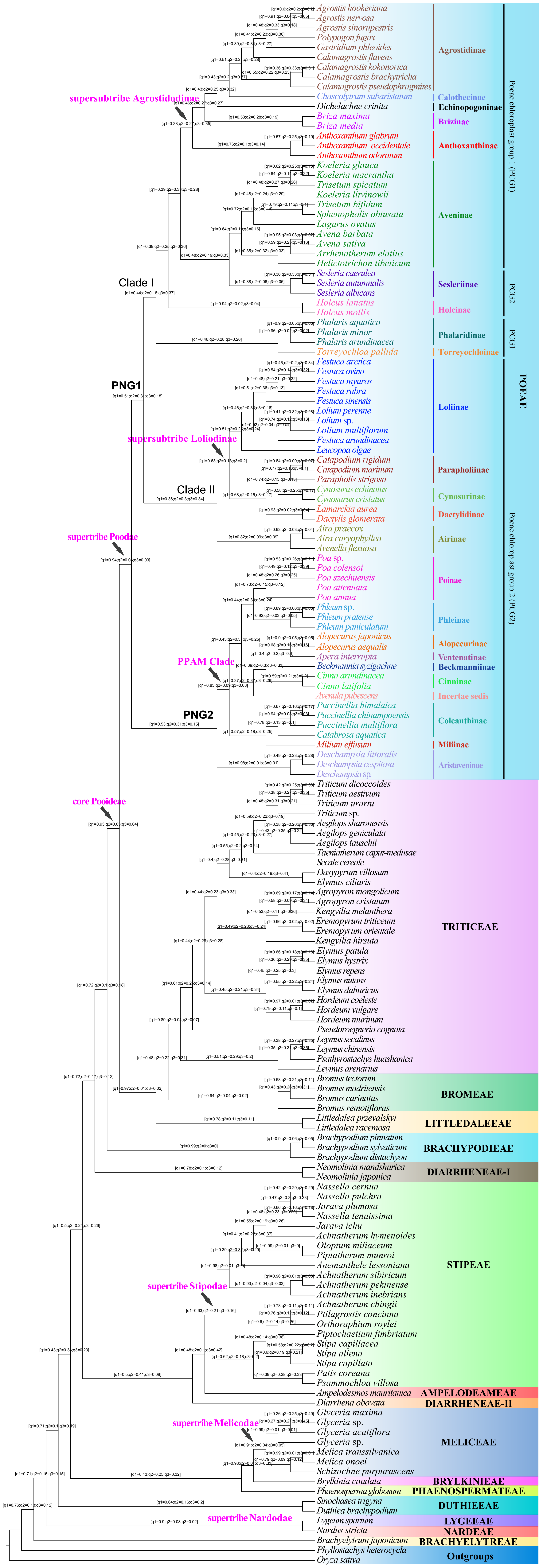

Figure S41 Branch annotation of quartet support values inferred from 763 OGs by ASTRAL.
Quartet support values are indicated in the following order: the main topology (q1), one for each of the two alternatives (q2 and q3). Tribes, subtribes and major lineages are the same as supplementary figs. S5-S9.

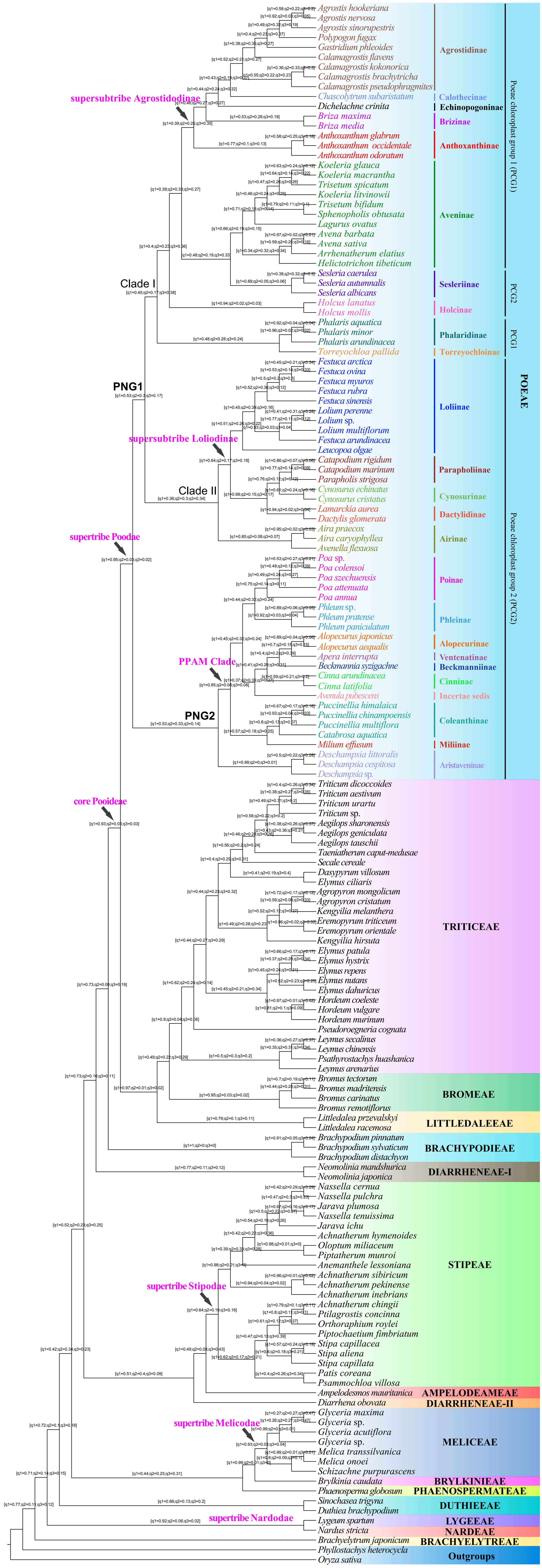

Figure S42 Branch annotation of quartet support values inferred from 512 OGs by ASTRAL. Quartet support values are indicated in the following order: the main topology (q1), one for each of the two alternatives (q2 and q3). Tribes, subtribes and major lineages are the same as supplementary figs. S5-S9.

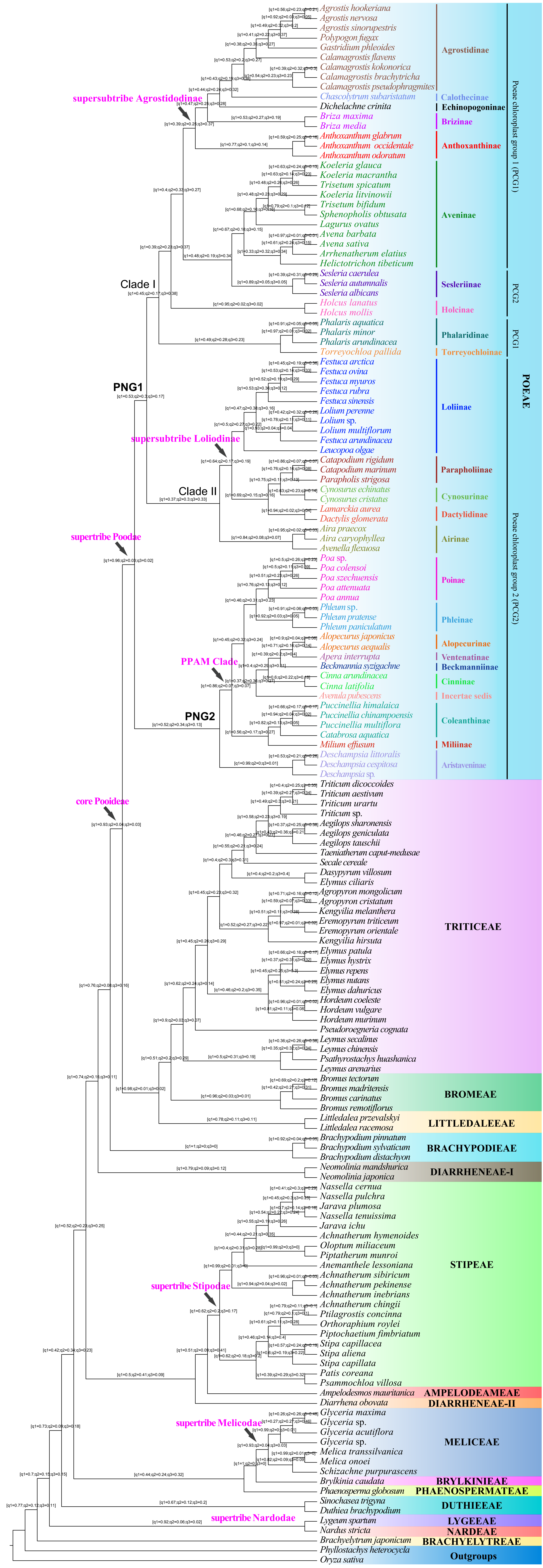

Figure S43 Branch annotation of quartet support values inferred from 373 OGs by ASTRAL.
Quartet support values are indicated in the following order: the main topology (q1), one for each of the two alternatives (q2 and q3). Tribes, subtribes and major lineages are the same as supplementary figs. S5-S9.

a

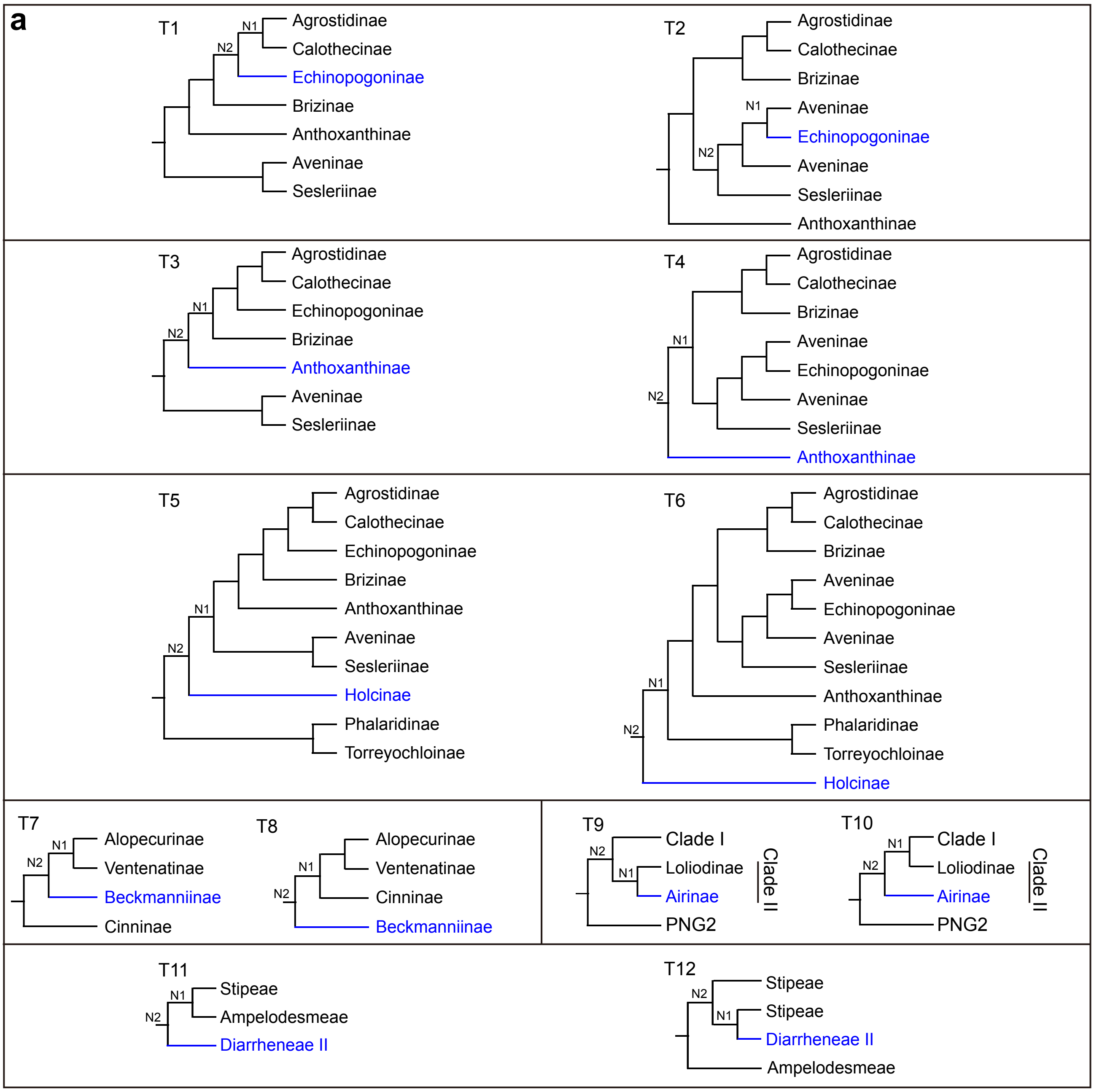

b

			Sigle copy					Multi-copy		
			1234	914	763	512	373	802	480	181
Echinopogoninae	Topology		T1	T1	T1	T1	T1	T2	T2	T2
	Support	N1	100	100	100	100	93	1	1	1
		N2	98	99	95	88	96	0.71	0.61	0.54
Anthoxanthinae	Topology		T3	T3	T3	T3	T3	T4	T4	T4
	Support	N1	93	90	91	81	96	0.76	0.69	0.65
		N2	93	89	91	79	83	0.68	0.6	0.5
Holcinae	Topology		T5	T5	T5	T5	T5	T6	T6	T6
	Support	N1	100	100	100	99	95	0.68	0.6	0.47
		N2	94	91	88	85	62	0.95	0.95	0.81
Beckmanniinae	Topology		T7	T7	T7	T7	T8	T8	T8	T8
	Support	N1	100	100	100	100	21	0.99	0.92	0.47
		N2	81	78	77	86	100	1	0.99	0.99
Airinae	Topology		T9	T9	T9	T9	T9	T10	T10	T10
	Support	N1	100	100	100	100	100	0.7	0.59	0.51
		N2	47	54	63	40	81	0.98	0.94	0.66
Diarrheneae II	Topology		T11	T11	T11	T11	T11	T12	T12	T12
	Support	N1	100	100	100	100	100	1	0.94	0.98
		N2	100	100	100	100	100	1	1	1

Figure S44 A comparison of topologies from single copy and multi-copy genes inferred by ASTRAL. (a) Clades with alternative placement of specific lineages, which are represented by blue branches. (b) Support values for alternative relationships from single copy (five OGs, bootstrap values) and multi-copy (three OGs, local posterior probabilities) analyses. The topology ID is as the same as (a).

Zhang et al.

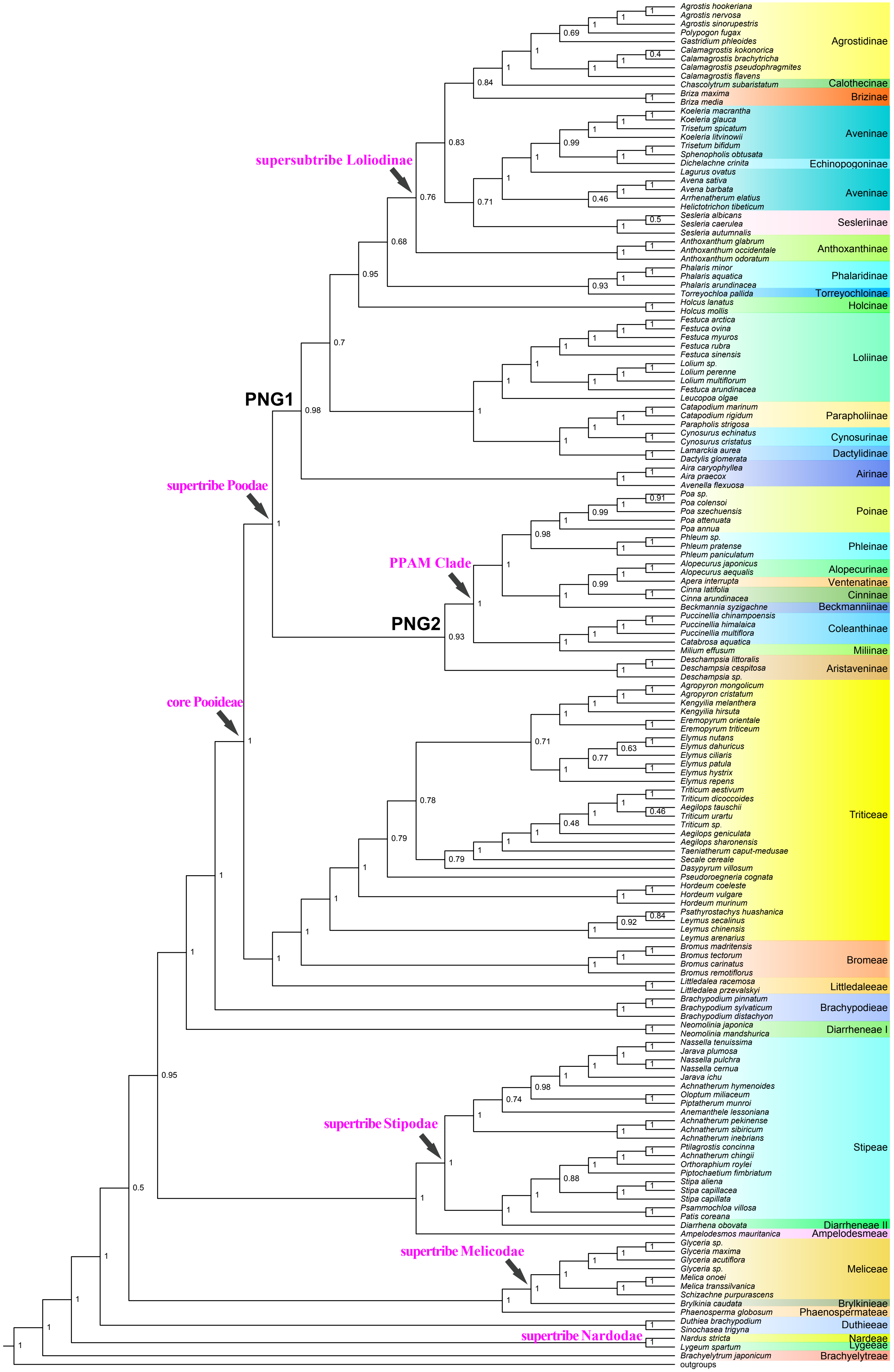

Figure S45 A phylogeny inferred from 802 OGs (multi-copy genes) by ASTRAL. Numbers at nodes indicate the local posterior probabilities. Tribes, subtribes and major lineages are the same as figs. 1-2.

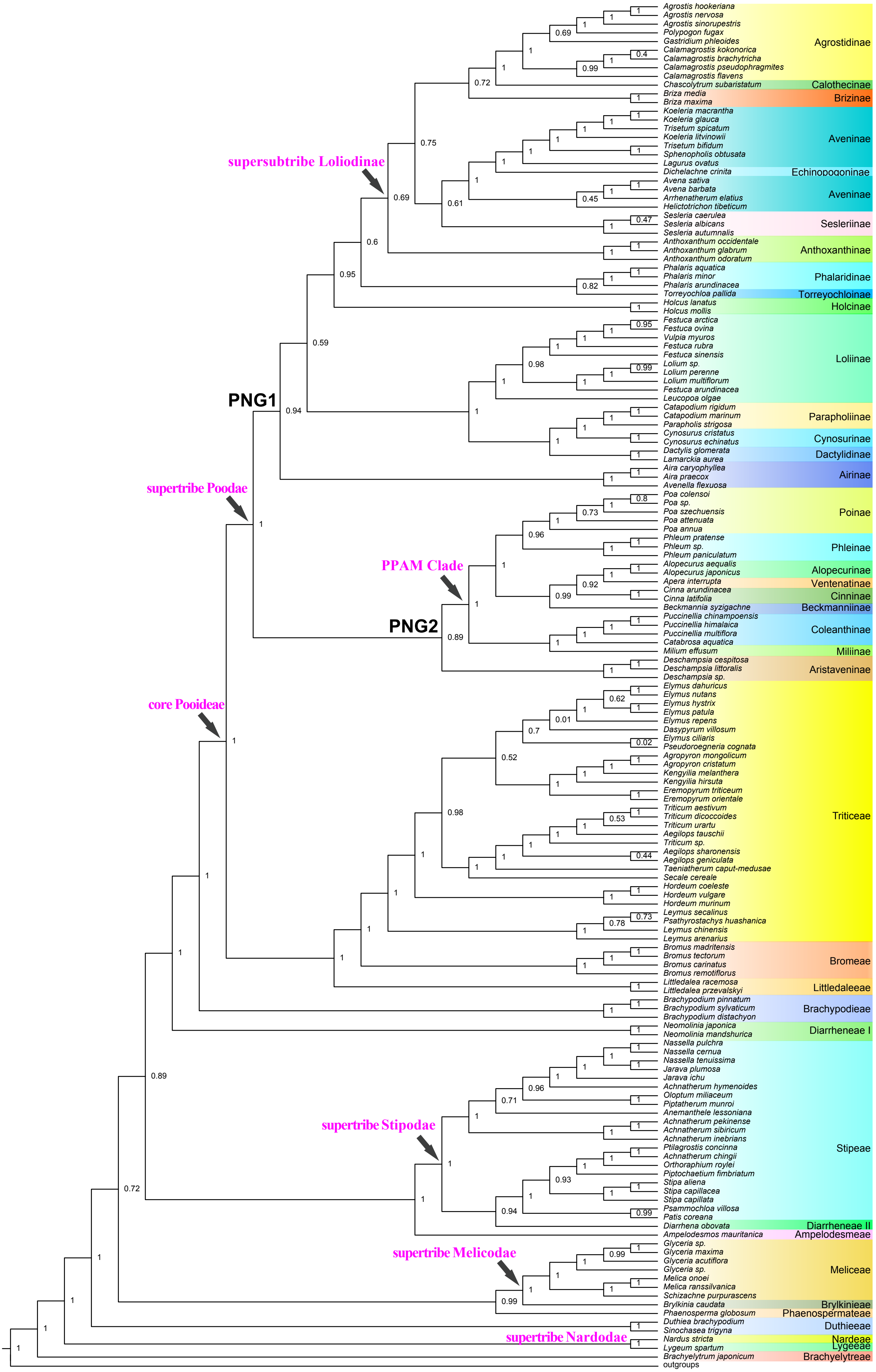

Figure S46 A phylogeny inferred from 480 OGs (multi-copy genes) by ASTRAL.
Numbers at nodes indicate the local posterior probabilities. Tribes, subtribes and major lineages are the same as figs. 1-2.

Zhang et al.

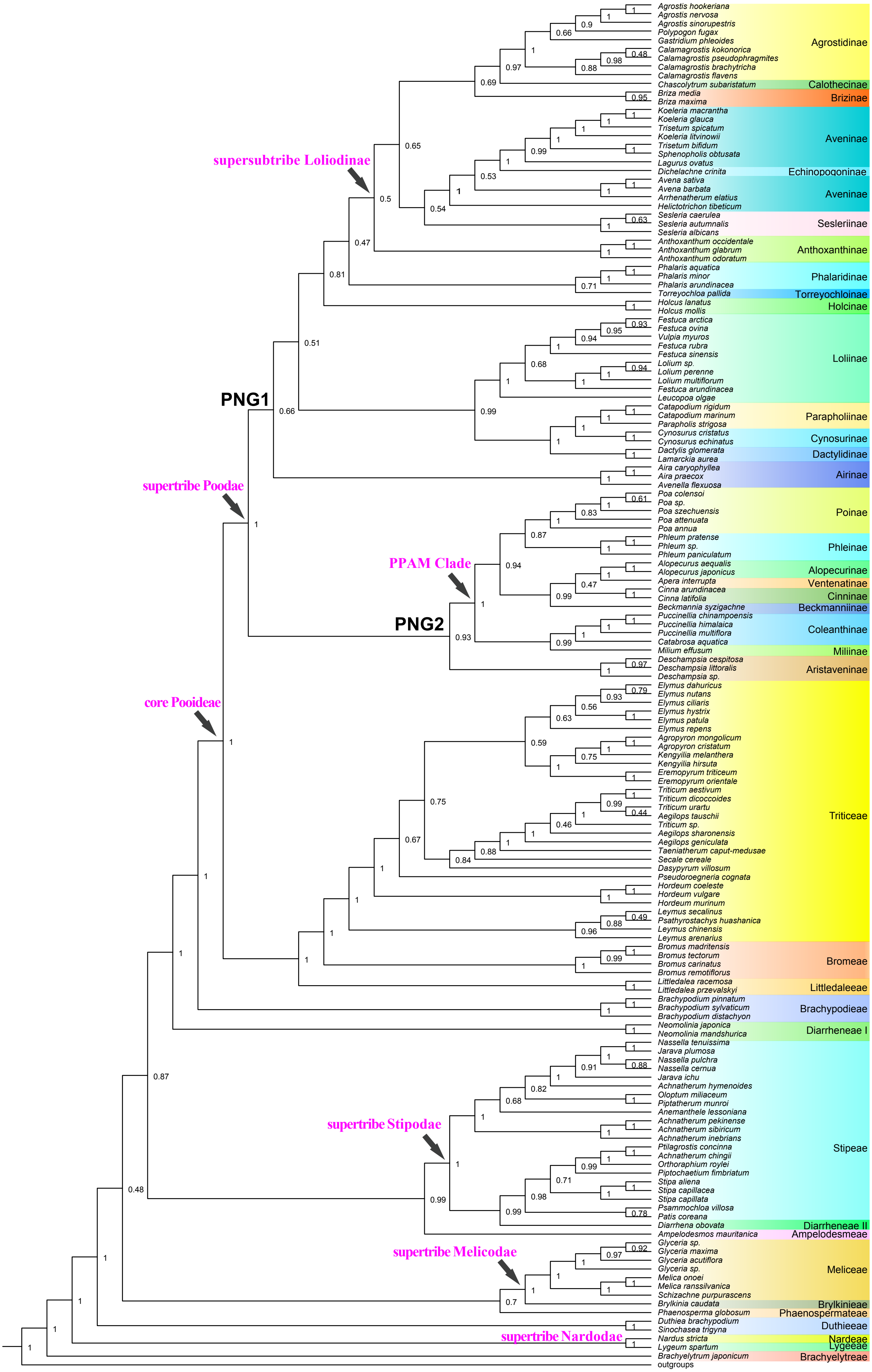

Figure S47 A phylogeny inferred from 181 OGs (multi-copy genes) by ASTRAL. Numbers at nodes indicate the local posterior probabilities. Tribes, subtribes and major lineages are the same as figs. 1-2.
